# Long range gene flow beyond predictions from oceanographic transport in a tropical marine foundation species

**DOI:** 10.1038/s41598-023-36367-y

**Published:** 2023-06-05

**Authors:** Ana I. Tavares, Jorge Assis, Patrick D. Larkin, Joel C. Creed, Karine Magalhães, Paulo Horta, Aschwin Engelen, Noelo Cardoso, Castro Barbosa, Samuel Pontes, Aissa Regalla, Carmen Almada, Rogério Ferreira, Ba Mamadou Abdoul, Sidina Ebaye, Mohammed Bourweiss, Carmen Van-Dúnem dos Santos, Ana R. Patrício, Alexandra Teodósio, Rui Santos, Gareth A. Pearson, Ester A. Serrao

**Affiliations:** 1 Center of Marine Sciences (CCMAR-CIMAR), Universidade do Algarve, Faro, Portugal; 2 Faculty of Bioscience and Aquaculture, Nord Universitet, Postboks 1490, 8049 Bodø, Norway; 3 Texas A&M University-Corpus Christi, Corpus Christi, TX USA; 4 Departamento de Ecologia, Universidade do Estado do Rio de Janeiro, Rio de Janeiro, RJ Brazil; 5 Área de Ecologia, Departamento de Biologia, Universidade Federal Rural de Pernambuco, R. Dom Manoel de Medeiros, s/n-Dois Irmãos, Recife, PE CEP 52171-900 Brazil; 6 Laboratório de Ficologia, Departamento de Botânica, Universidade Federal de Santa Catarina, Florianópolis, SC 88040-970 Brazil; 7 CARMABI Foundation, Piscaderabaai z/n, P.O. Box 2090, Willemstad, Curaçao The Netherlands; 8 CIPA, Centro de Investigação Pesqueira Aplicada, Bissau, Guinea-Bissau; 9 IBAP-Instituto da Biodiversidade e Áreas Protegidas, Bissau, Guinea-Bissau; 10 Faculdade de Ciências e Tecnologia, Universidade de Cabo Verde, Praia, Cabo Verde; 11 Dragões do Mar, Nova Estrela, Ilha do Príncipe, São Tomé and Príncipe; 12 Parc Nationale du Banc d’Arguin (PNBA), Chami, Mauritania; 13 Institut Mauritanien de Recherche Oceanographique et des Peches (IMROP), Nouadhibou, Mauritania; 14 Universidade do Namibe, Namibe, Angola; 15 MARE-Marine and Environmental Sciences Centre, ISPA-Instituto Universitário, Lisbon, Portugal; 16 Centre for Ecology and Conservation, University of Exete, Penryn, UK; 17 CIBIO-InBIO, Centro de Investigação em Biodiversidade e Recursos Genéticos, Vairão, Portugal

**Keywords:** Biodiversity, Marine biology, Biogeography, Population genetics

## Abstract

The transport of passively dispersed organisms across tropical margins remains poorly understood. Hypotheses of oceanographic transportation potential lack testing with large scale empirical data. To address this gap, we used the seagrass species, *Halodule wrightii*, which is unique in spanning the entire tropical Atlantic. We tested the hypothesis that genetic differentiation estimated across its large-scale biogeographic range can be predicted by simulated oceanographic transport. The alternative hypothesis posits that dispersal is independent of ocean currents, such as transport by grazers. We compared empirical genetic estimates and modelled predictions of dispersal along the distribution of *H. wrightii*. We genotyped eight microsatellite loci on 19 populations distributed across Atlantic Africa, Gulf of Mexico, Caribbean, Brazil and developed a biophysical model with high-resolution ocean currents. Genetic data revealed low gene flow and highest differentiation between (1) the Gulf of Mexico and two other regions: (2) Caribbean-Brazil and (3) Atlantic Africa. These two were more genetically similar despite separation by an ocean. The biophysical model indicated low or no probability of passive dispersal among populations and did not match the empirical genetic data. The results support the alternative hypothesis of a role for active dispersal vectors like grazers.

## Introduction

Long-range dispersal of marine organisms with passively transported propagules is often hypothesized to be mediated by ocean currents, a hypothesis that lacks much empirical testing despite its major ecological and evolutionary implications^1,2^. Challenges related with empirically tracking the movement and outcome of passive dispersal units have narrowed our knowledge on population connectivity across most marine biodiversity^3^. Indeed, most marine species, particularly habitat-forming species (macroalgae, corals, or seagrasses), migrate via passively-dispersed propagules^4,5^. Thus, these species, are expected to form metapopulations that are passively connected by the transportation of such dispersal stages^6^, but the processes mediating such transportation are poorly understood. Many such marine species, including seagrasses, have wide geographical distribution ranges, which contrasts with their predicted dispersal capacity or ability to maintain regular long-distance dispersal throughout their range^7,8^. Biological features of the dispersal phase, its interaction with a range of abiotic, historical, and biotic factors, determine species range limits and gene flow among populations^9^, which significantly impact a species’ distribution and persistence^10^.

Large-scale studies focused on seagrass population connectivity have been developed^11–13^ but none have addressed the long distance cross-ocean dispersal in the tropical Atlantic region^14^. Along this wide geographical region, seagrass populations might be isolated by biogeographic barriers such as vast oceanic distances (e.g., the thousands of kilometres that separate east and west Atlantic), lack of suitable habitat (e.g., freshwater discharge near the Amazon or Congo rivers), or main oceanographic currents (e.g. Caribbean Current and South Equatorial Current^15,16^). Leading hypotheses predict that oceanographic currents determine most connectivity of passively dispersed stages, functioning as both barriers and promoters of gene flow. The tropical Atlantic region, rich in seagrass diversity^14^, has only local-scale assessments focused on seagrass genetic diversity, dispersal and connectivity^16–18^. Four seagrass genera are known to dominate the tropical Atlantic—*Thalassia*, *Syringodium*, *Halophila,* and *Halodule*—which either occur as single species or intermixed^19^. However, only *Halodule wrightii* is distributed on both east and west tropical Atlantic coastal shores, serving as an optimal model for range-wide connectivity studies across pan-Atlantic spatial scales.

Seagrasses form one of the most productive and important ecological groups along with mangroves and corals. They are flowering marine angiosperms that can form dense meadows in shallow coastal waters around the world^20,21^. Seagrasses play a strong role in structuring communities and have received much attention due to their large ecological and social significance^22^. However, they are under increasing pressure from anthropogenic activities and climate change^23,24^. As a result, seagrass habitats are declining globally^23^, becoming lost or fragmented, and further extinctions are forecasted under future climate change scenarios^25^.

Seagrasses can reproduce sexually by seed production and asexually by vegetative clonal development^26^ and each reproductive mode produces different types of propagules with diverse dispersal abilities^4,9^. There are several circumstances that can drive seagrass propagule dispersal routes over time. Abiotic factors such as wind, waves, tides or currents can lead seagrass-detached fragments to be easily transported away from the source location^27^. In contrast, seabirds, fish, sea turtles, and dugongs are examples of biotic vectors that may mediate seagrass migration and gene flow by transporting clonal propagules or dispersing seeds after passing through their digestive system^28,29^, increasing the chances, or even boosting, their germination success^30^. Moreover, propagule features such as seed buoyancy can mediate dispersal scenarios^4^. These biophysical interactions (between propagule features and transport mechanisms) are predicted to shape the extent of connectivity among seagrass meadows over large geographical scales and to determine population longevity^4,8^.

To address this knowledge gap, this study aims to compare predictions and empirical data on large scale connectivity for the seagrass species *Halodule wrightii*, the tropical species with the most extensive geographic range across the Eastern and Western Tropical Atlantic Ocean^31^. As for all in most other seagrass species, *H. wrightii* reproduces asexually and sexually, and the extent of each reproduction mode influences dispersal, genetic diversity, and biogeography. Despite its widespread distribution, the species produces seeds that are neutrally to negatively buoyant, with an apparent lack of long distance dispersal means^32^. Seeds of *H. wrightii* are able to form persistent seed banks that can remain dormant up to 4 years^33^. Therefore, seeds play an important role in the persistence of the species. *H. wrightii* can also colonize new areas through by asexual clonal growth of detached fragments that can survive in the water column for up to 1 month^34^. Previous studies have explored the genetic structure and connectivity of *H. wrightii*^8,18,35^ but only focused on specific areas of its distribution. To date, there is not a comprehensive study that encompasses this tropical amphi-Atlantic seagrass, in contrast with other seagrass species located on a single side of this ocean, such as *Cymodocea nodosa*^36^.

Here, we aim to test the hypothesis of ocean currents as the main vectors for dispersal of *H. wrightii* in the Atlantic Ocean by comparing oceanographic predictors with genetic information. This is the first study that considers the distributional range of *H. wrightii*. The main goal of the present study was to uncover the population genetic structure and connectivity of this habitat-forming seagrass species in the tropical Atlantic region. This species is an ideal model for range-wide connectivity studies due to its vast geographical distribution. Here, we ask if: (1) there is genetic structure among *H. wrightii* populations across distant biogeographic regions along the tropical Atlantic; (2) levels of genetic and genotypic diversity are similar among populations; (3) the main ocean currents explain the present genetic structure; or (4) if the latter could be explained by biotic transportation. These questions and hypotheses were approached by using microsatellite markers and biophysical modelling based on oceanographic transport to model connectivity along the vast *H. wrightii* distributional range.

## Results

**Figure 1:**
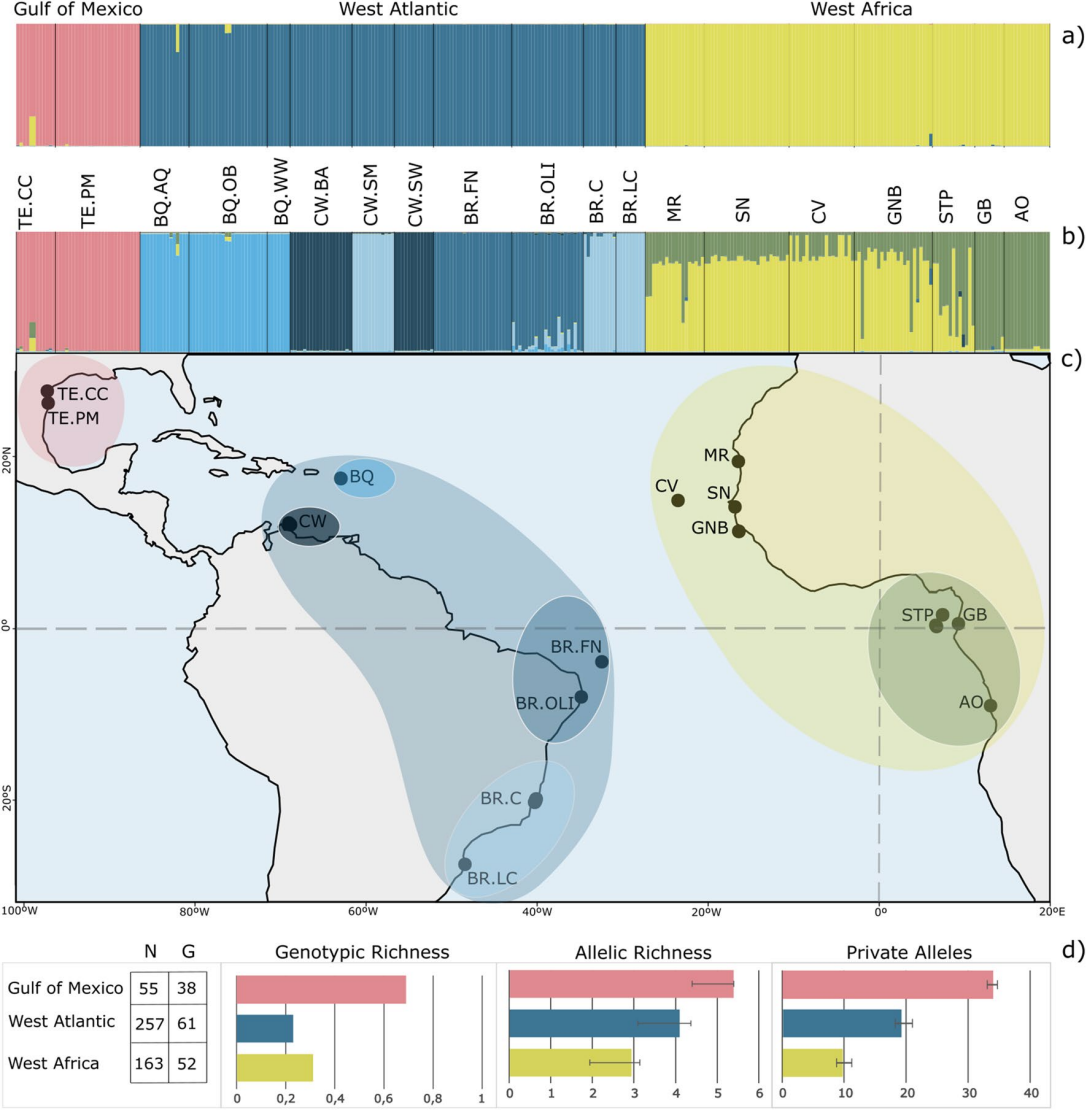
(**a, b**) Assignment of individuals to genetic groups that minimize Hardy–Weinberg and linkage disequilibria, estimated by STRUCTURE; colors depict the genetic subdivision based on K = 3 and K = 7 levels of subdivisions; Site names and coordinates are listed in Tables S1 and S4; (**c**) Sampling sites of *Halodule wriightii* with colors depicting the main highest genetic differentiation inferred with STRUCTURE (K = 3 groups); (**d**) Sample size (N); number of unique genotypes (G); genotypic richness, standardized allelic richness and standardized number of private alleles for the smallest common sample size. The figure was generated using R (version 4.2.2) and Inkscape 1.2.1 (https://inkscape.org/).

No evidence of null alleles nor linkage disequilibrium was found. For the *H. wrightii* ramet dataset, we obtained a total of 475 sampling units (ramets) successfully genotyped for eight microsatellite loci across 19 populations. For the genet-level dataset, after the removal of repeated copies of each genet, 176 individual genotypes remained for analysis. Measures of genetic diversity such as allelic richness (i.e., the mean number of alleles per locus within a population) and gene diversity, or expected heterozygosity (i.e., the probability that two randomly selected alleles at a particular locus will be different in a population) were generally low for ramet and genet level datasets within all populations (Fig. 1D; Tables S1 and S2), except for both populations in the Gulf of Mexico. Likewise, private alleles ranged from high values in the same region to nearly no private diversity in several sites of the Caribbean region but also in West Africa (see Fig. 1D; Tables S1, S2). All the genetic diversity parameters when analyzed for the first hierarchical level of genetic structure (k = 3) revealed high estimates higher in Gulf of Mexico than the other clusters (Table S4).

Most of the inbreeding coefficients (F_IS_) estimates were significantly negative for both datasets (Tables S1–S3), showing heterozygotes to be more prevalent than expected under Hardy–Weinberg equilibrium. The opposite scenario was observed for Gulf of Mexico, where heterozygote deficiency was prevalent (Tables S1 and S2). Also, F_IS_ values across the different locations in both datasets exhibit a wide range of negative values dispersion (Fig. S2)**.** Genotypic richness (R) was highly variable among populations on both sides of the Atlantic, ranging across both extremes (Tables S1 and S2), from close to maximal contribution of sexual propagation (in the east Atlantic 88% of sampled shoots were distinct genets in Santana and Gabon, and in the west 93% were distinct in the Gulf of Mexico) to predominance of a single clone (only 8% were distinct genets in Angola in the east and in Curaçao in the west).

Pairwise differentiation (FST) between groups showed higher differentiation between West Africa against the other groups but differentiation was lower among West Africa populations (Fig. S3).

**Figure 2:**
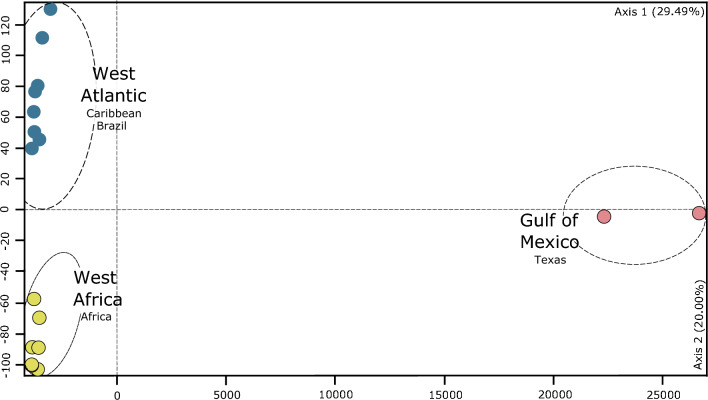
Genetic differentiation of populations of *Halodule wrightii* illustrated by factorial correspondence analysis (FCA). Distances in the plot are proportional to genetic divergence, illustrating that the divergence between the Gulf of Mexico and the other populations is much higher than that among any other populations.

The factorial correspondence analysis (FCA) revealed a first major genetic differentiation between the Gulf of Mexico and the remaining sites, and these latter also split into east and west, resulting in three genetic clusters: Gulf of Mexico, east Atlantic (West Africa), remaining west Atlantic (Brazil and Caribbean) (Fig. 2). The STRUCTURE analysis, along with DAPC and PCA indicated the same three genetic clusters and at a more fine-scale differentiation these were subdivided into seven clusters (Fig. 1A and Fig. S4). These revealed a differentiation between southern and northern populations in Africa, admixed in Sao Tome and Principe, and a differentiation into four clusters in the Caribbean-Brazil region, admixed in Curaçao (Fig. 1B, C). Also, STRUCTURE plot for K = 7, revealed more similarities between one of the Curacau populations (CW.SM) and the ones on south Brazil (Fig. 1).

**Figure 3:**
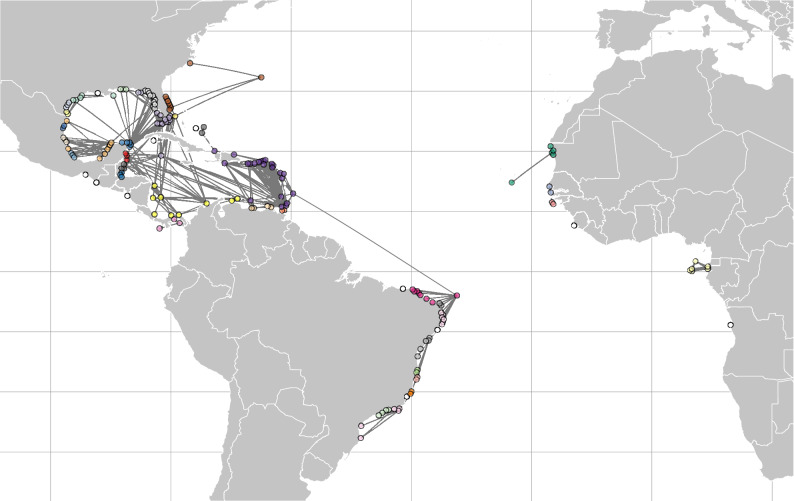
Potential connectivity among *Halodule wrightii* populations that have been reported in literature and databases (see Table S5), estimated from simulations of transport by ocean currents data. The figure was generated using R (version 4.2.2).

The data compiled from literature (Table S5, supplemental material) and biodiversity databases, yielded 1815 known locations for the species. This resulted in 183 distinct source/sink sites aggregated at 1 km, which were included in the biophysical modeling*.* The particle simulations delivered 668,499 particles over a 10-year period. These revealed a sharp decline in potential connectivity with distance (Fig. S1), with high probabilities for propagule retention near the source locations (Fig. S1). Most connectivity events were predicted to take place only at regional scales (average distance of connectivity events: 330.42 ± 450.11 km; maximum: 3766.80 km), with low probabilities of connectivity (average probability of connectivity: 0.10 ± 0.19) due to few pair-wise site connectivity events (average number of events: 36.67 ± 70.65) (see Supplementary Information). The models predicted large scale oceanic transportation from Fernando Noronha (offshore Brazil) to Barbados in the Caribbean Sea (Fig. 3). Geographic distant regions had no probability of connectivity mediated by ocean currents (i.e., no connectivity events occurred at all from/to these sites in the 10 years of simulations), indicating zero probability that currents could be predominantly responsible for the dispersal and genetic differentiation of *H. wrightii* across such spatial and temporal scales.

## Discussion

The genetic structure of the seagrass *H. wrightii* over its distributional range revealed three main genetic groups separated geographically at different spatial scales (from a broad to a regional scale), that did not completely match predictions from oceanographic transport of propagules (seeds or shoots) by currents. These results showed that although ocean circulation is an important factor in marine population structure^1^, long-distance dispersal is likely a very rare event. The results support the hypothesis that rare events of occasional biotic transportation might have been responsible for connectivity between *H. wrightii* populations that belong to distinct genetic clusters.

### Genetic structure and connectivity

#### Large scale connectivity

The significant genetic differentiation between West Atlantic, Gulf of Mexico, and West Africa, indicates restrictions to gene flow between these three main genetic lineages of *H. wrightii*. This was also supported by the biophysical modelling that revealed a null or very low probability of connectivity mediated by ocean currents between distant geographic regions (e.g., between east and west Atlantic coastlines) and suggested that most of the gene flow mediated by currents occurs at a regional scale (up to ~ 1230 km, Fig. S1). Such skewed relationship between oceanographic connectivity and distance has been previously reported from biophysical model predictions, regardless of the dispersal potential of the species of interested. Additional studies using biophysical modelling to address the role of oceanographic connectivity to the distribution of, e.g., mangrove forests (high dispersal) and macroalgae (reduced dispersal) also showed null or very low predicted probability of connectivity across large water masses^37–39^. Besides the null probability of transport by currents, distance and habitat discontinuity are likely to cause isolation between east and west Atlantic shores, as well as other physical processes, such as waves and direct forcing by winds, that are not accounted for in the HYCOM model. Predictions of long distance connectivity patterns through ocean currents benefit from knowledge of key life history traits such as seed or shoot viability times and establishment success^4^. Some of these details are unknown for *H. wrightii*, although there is information on such life traits for other seagrass species^40,41^, few of which facilitate oceanic rafting such as positively buoyant seeds or fruits^27,42^. However, *H. wrightii* has low seed dispersal potential because its seeds develop near the sediment at the base of the shoots and are negatively buoyant. Detached fragments may therefore be more likely to raft with ocean currents and they can survive floating in the water column up to 4 weeks^34^, although the subsequent establishment success has not been determined. Yet the ability of seeds to remain in a dormancy stage for at least up to 46 months^33^ supports the possibility that they might be remain viable during occasional long-range dispersal, by either abiotic or biotic vectors.

Abiotic transport by ocean currents may not require the capacity to float if seeds become entangled on other floating rafts, such as those formed by *Sargassum*, that can cross the Atlantic Ocean in less than 2 weeks^43^, a timeframe shorter than the viability of *H. wrightii* seeds and detached shoots. Rafting *Sargassum* have been recorded to move up the North Brazil current to the Caribbean and eastward towards western Africa^44,45^ and seagrass fragments have been found among *Sargassum* rafts^46,47^. Moreover, migratory species like sea turtles^46^ and seabirds^48^ use *Sargassum* mats and may facilitate dispersal. Alternative or complementary to ocean currents, seagrass propagules may also be transported to suitable habitat by grazers that use these mats as foraging sites.

Despite its low abiotic dispersal potential, *H. wrightii* has a wide distribution with variable genetic differentiation, and among all tropical seagrasses of the western Atlantic it is the only species that has colonized the eastern side of this ocean, which suggests long-distance dispersal capabilities. This apparent paradox supports the hypothesis of an important role for biotic transportation of its seeds by grazers, as proposed in other studies^8^. Megaherbivores, including dugongs, green turtles, some birds and fish, use seagrass as one of their food sources, and promote dispersal of seagrass seeds^49^. Successful seagrass endozoochory (i.e., seed dispersal by animals after passage through their guts) has been reported for fish, sea turtles and birds^7,28,50,51^. The dispersal distances of such animals known to feed among seagrass meadows would allow long-ranging dispersal (e.g., 277 to 652 km in green turtles or 173 to 234 km by dugong^29^). The reported digestion times for such herbivores, 6–7 days for dugongs^29,52^ and 1–2 weeks for green turtles^53^, are compatible with a moderate scale of long-range transport. Satellite tracking of green turtles in Atlantic Africa shows travel of 40 to > 1000 km between *H. wrightii* sites^54^ and genetic data indicate cross Atlantic green turtle migration^55^. Still, direct cross-Atlantic biotic dispersal seems unlikely or at least uncommon within average digestion time scales of grazers. Migratory routes of green turtles that feed on *H. wrightii*^56^ include several mid-Atlantic islands that are nesting grounds^55^, and could serve as occasional stepping-stones, although seagrass presence is mainly unknown in such islands^57^. It is relevant that a green turtle found dead in Senegal had been nesting in Trindade Island 5 months before^58^. All this information and our data indicate that transoceanic dispersal must be a very rare event, because the distances may be too vast to allow continuous gene flow even by biotic vectors, and there is a remote hypothesis that stepping stone islands could facilitate occasional dispersal of viable seeds. However, even if propagule dispersal is successful, post-settlement survival may be low, dependent on ending dispersal in a favorable environment^34^.

#### Fine scale connectivity

On the western Atlantic coastline, the biophysical model revealed high probability of oceanographic connectivity between populations, but the genetic differentiation into distinct clusters showed that gene flow is restricted among them. Genetic data revealed isolation between Gulf of Mexico, Caribbean, and Brazil despite the high probability of transport by oceanographic currents, particularly between the Caribbean and Gulf of Mexico. However, no apparent gene flow barrier exists between the Caribbean and the Gulf of Mexico, in contrast with the genetic structure shown in our findings and in previous seagrass studies using microsatellites^16,42^. This is suggesting that this genetic differentiation might be due to recolonization events that occurred in the Caribbean and Gulf of Mexico after the last glacial maximum^59^.

The low probability of connectivity between the Caribbean and Brazil coincides with a biogeographical barrier created by the discharge from the deltas of the Amazon and Orinoco rivers, among others^60^. This barrier, which runs for around 2300 km, separates the Brazilian coast from the Caribbean region and affects the genetic structure and dispersal of different marine animal organisms as fish or coral species^61,62^. Yet, this barrier is not matched in our genetic data, that suggests gene flow among these regions. Thus, it is plausible to hypothesize that biotic dispersal might also play a role in increasing *H. wrightii* dispersal potential between these regions. Sea turtles and manatees are known to feed on *H. wrightii* meadows in these regions^63^, and for some species, these areas are part of their migratory pathways^64^. Moreover, our data also suggests a closer level of similarity between population of these two regions (k = 7 structure plot; Fig. 1). However, this result must be interpreted with caution as it might be attributed to homoplasy (i.e., individuals with different ancestries that mutate at a locus to the same allele). Homoplasy due to mutation is expected to occur for microsatellite markers due to their allele size and high mutation rates. Therefore, it is important to consider the potential for homoplasy when using microsatellites to infer relationships among individuals or populations.

Despite the restricted ocean-driven dispersal potential of *H. wrightii*, population genetic differentiation was non-significant across most populations in West Africa, indicating significant inter-population connectivity. The genetic structure of *H. wrightii* in Africa and in general, the genetic evidence indicating long-distance migration where it is not predicted, and high differentiation in some places at short distances, support the hypothesis of animal-mediated transportation^8^.

### Genetic diversity

Genetic diversity contains the footprints of population stability, mating, and dispersal ecology, and is therefore used here as a proxy of the historical ecology of the populations. The higher genetic diversity of *H. wrightii* in the Gulf of Mexico supports previous reports^18,35^ suggesting the Gulf of Mexico should be a genetic hotspot for seagrass conservation. This suggests long-term stability without major bottlenecks.

Additionally, genetic diversity decreased in range-edge populations in both the east and west Atlantic (Angola and south Brazil, respectively), as reported for other seagrass, mangrove, and coral species. Low diversity at range edges is common^11,65^ and might be influenced by less suitable habitat increasing the reliance on clonal propagation, population bottlenecks or a relatively recent founder event during colonization of these regions from a larger more central population, resulting in genetic variation loss.

In comparison to other tropical seagrass species, the general microsatellite genetic diversity of *H. wrightii* was lower than *Enhalus acoroides*^66^, *Thalassia testudinum*^16^ and *Zostera japonica*^67^, but equivalent to *Halophila beccarii*^68^, *Cymodocea serrulata*^11^, *Syringodium filiforme*^17^ and *Zostera marina*^69^. Across the *H. wrightii* populations, negative inbreeding coefficients (F_IS_) were obtained, which is common in seagrass species^16,18,70^ and supports the hypothesis of selection favoring heterozygous individuals.

### Genotypic richness

Although*, H. wrightii* has been able to achieve such a vast distribution, its dispersal success alone cannot ensure establishment, because that is also influenced by survival, viability, and growth capacity of plants originated from seeds and fragments. The balance between sexual reproduction and clonal growth can be represented by the genotypic richness. Despite the small sample sizes in some populations, our findings revealed that genotypic richness of *H. wrightii* is very variable among locations. Higher genotypic richness values were found in populations located at the center of the distribution. Higher genotypic diversity (i.e., a population with a larger number of distinct genotypes or clones) may enhance productivity and community recovery^71,72^. Seagrass genotypic diversity can range from near monoclonal^36^ to very high^73^.

Reduced genotypic richness can be a common scenario for coastal populations found at the species’ range edge^36,74^. Indeed, clonal propagation was the dominant reproductive mode prevalent in southern marginal populations across east and west Atlantic, showing edge-of-range reduced sexual reproduction relative to populations at center of the species’ range (geographic parthenogenesis)^75,76^. Such a pattern could be attributed to limited population sizes, geographical isolation, climate oscillations, and poor seed dispersal from other source populations. However, the northern marginal population in West Africa (Banc d’Arguin, Mauritania), revealed a much higher genotypic richness compared to other marginal populations. This may be due to the Banc d'Arguin’s habitat suitability for seagrass species, as opposed to what would be predicted for a marginal range. The Banc d'Arguin is huge, protected, shallow, and nutrient-rich, with inputs of desert dust from the east and coastal upwelling from the northwest^77^. Furthermore, this tropical seagrass species is projected to expand there with climate change^78^.

Our findings showed that *H. wrightii* relies primarily on clonal propagation throughout the Caribbean region. Seagrasses reproduce both sexually and asexually, and their relative proportions may depend on several factors, including the level of disturbance^79^. Specifically, events that destroy seagrass meadows create opportunities for rapid colonization and growth by new shoots or clonal fragments, faster than through seed production^80,81^. There are reports of fast establishment of *H. wrightii* after a hurricane (e.g. hurricane Wilma in 2005^82^). Thus, we hypothesize that the low genotypic richness found among Caribbean populations of *H. wrightii* could be due to past disturbances followed by fast colonization^83^ through asexual reproduction of previously established or selected genotypes.

Overall*, H. wrightii* showed a low mean of genotypic richness (R = 0.38), lower when compared to the mean genotypic richness found for other Atlantic seagrass species such as *Zostera noltii* (R = 0.85)^84^, *Zostera marina* (R = 0.61)^85^ or *Thalassia testudinum* (R = 0.55)^73^, but comparable to *Syringodium filiforme* (R = 0.37^17^). However, seagrass populations can show a wide range of genotypic richness, from high rates of sexual reproduction^86^, to high levels of clonality^73^ such as in *Z. noltei*^87^ and also *H. wrightii*^18,35^*.*

## Concluding remarks

We found discordance between genetic differentiation and predicted ocean connectivity patterns, suggesting a role for other means of dispersal, such as the hypothesis of grazers mediating transport. This study underlines the importance of taking multi-pronged approaches to understand meta-population dynamics and connectivity. Using genetic data and modelling predictions enabled us to study the dispersal and connectivity patterns at different spatial scales while testing hypotheses of ocean currents mediating connectivity.

Genetic differentiation requires time for evolutionary processes to accumulate population differences. Therefore, although differentiation requires the lack of homogenizing gene flow, the absence of differentiation can be caused by either gene flow or insufficient time for differentiation among recently isolated populations, also designated as shared ancestral polymorphism. We used genetic methods as an empirical indicator of gene flow patterns, and biophysical modeling through propagule dispersion simulations to complement our findings. All these methods, like any other ways of assessing connectivity, have inherent drawbacks that should be highlighted. Genetic differentiation is affected by historical factors, such as founder events, bottlenecks, and variable population sizes. While this offers a significant advantage in discussing such processes, it also poses a limitation in addressing dispersal as the single cause of the patterns, given the simultaneous influence of these other processes. Additionally, the possibility of remaining ancestral polymorphism could create the false impression of contemporary gene flow even where populations are presently isolated. These complex demographic processes like population bottlenecks, founder events, or changes in population size over time can affect the genetic structure of populations in addition to gene flow. In our data interpretation, we therefore considered that there are other ecological factors besides ocean currents, such as habitat continuity and biological dispersal features, that may have an impact on gene flow, raising the hypotheses of influences of large flow of major rivers in breaking habitat continuity, and grazer-mediated transport.

Our results also shed light on the population genetic variability of an important tropical seagrass across its entire range. This new information for *H. wrightii* can be used to highlight areas where local protection is necessary or where populations could be managed in a metapopulation strategy. It could also be used as a baseline for future comparisons of the genetic diversity and differentiation of this species, motivating further research. The study also comprises a valuable and unique species distribution baseline confirmed by genetic tools, which may be particularly useful in future studies for example of cases where the validity of putative sister taxa to *H. wrightii* may be unclear.

## Methods

### Study area and sampling

The present study encompasses the distributional range of *H. wrightii* along the east and west Atlantic coastlines (Fig. 1C), which ranges from Mauritania (19.5° N, 16.5° W; Africa) to Angola (9.0° S, 13.0° E; Africa) and from Gulf of Mexico (27.7° N, 97.3° W; USA), including the Caribbean Sea, to Brazil (27.6° S, 48.4° W; Brazil). A variable number of shoots was collected with local permissions at 32 localities (Table S1) according with each country guidelines. Samples collected before 2018 were taken mostly from herbarium collections, resulting in distinct sample sizes. Following 2018, approximately 20 shoots were sampled in shallow and subtidal areas (up to 5 m depth depending on water turbidity) by keeping a minimum distance of approximately 1 m between each sampling unit. At some locations, only small patches were found, resulting in a lower sample size (Table S1). After field collection, all individual plants were dehydrated on silica gel drying crystals. Specimens were identified first by the local teams and later by EAS, voucher samples of the dried plants or their DNA were deposited at the herbarium of the University of Algarve (see Data availability statement).

### DNA extraction and genotyping

Genomic DNA was extracted using the NucleoSpin Plant II Kit (Macherey–Nagel, Duren, Germany) following the protocol from the supplier. Individuals were genotyped for eight microsatellite markers (Table S6) developed for *H. wrightii*^18,88^. Polymerase chain reactions (PCRs) were performed in a total volume of 15 μL, containing 1× Colorless GoTaq" Flexi Buffer (Promega, Madison, WI, USA), 2 mM MgCl2 (2.5 mM), 10 mM forward and reverse primers, 0.2 mM of dNTP’s, 1 U GoTaq G2 Flexi DNA Polymerase (Promega, USA) and 5 mL of diluted template DNA (several dilutions for different populations). PCR conditions included an initial denaturation step at 95ºC for 5 min, followed by 30 cycles of 95ºC for 30 s, *T*_a_ for 30 s, extension at 72 °C for 30 s and a final extension at 72 °C for 10 min. Amplified fragments were separated on an ABI3130 XL automated DNA sequencer (Applied Biosystems, Waltham, MA, USA, at the CCMAR sequencing facility), with 0.25 mL GeneScanTM 500" LIZ Size Standard (Applied Biosystems, UK) plus 9.75 mL of Hi-Di formamide, after denaturation at 95 °C for 5 min. Alleles were manually scored using STRAND (Veterinary Genetics Laboratory, University of California, Davis; http://www.vgl.ucdavis.edu/STRand), and binned using the R package ‘MsatAllele’^89^. To minimize all ambiguities, a manual review of microsatellite amplification and scoring was conducted, and the final genotyping alleles for each sample were obtained after a double-check reading process to reduce scoring errors.

### DNA statistical analyses

The software Micro-Checker 2.2.3^90^ was used to check for null alleles to avoid bias in estimating genetic parameters. The package ‘RClone’^91^ of R software v.3.6.2^92^ was used to assess clonality for all individuals, because seagrass can proliferate vegetatively and the number of shoots can represent duplicate genotypes from the same clone. To verify if individuals with the same number of multi-locus genotype (MLG) were true clones, *P*_sex_ was calculated, i.e., the probability of finding identical MLGs resulting from distinct sexual reproductive events^93^. When *P*_sex_ threshold was below 0.01, the identical MLGs were considered the same clone (i.e., genet). Following previous theoretical assumptions^94^ we estivated population genetic metrics with our dataset spited into (1) a ramet level dataset, that includes genetic information for all genotyped individuals, and (2) a genet level dataset, where only the genets were kept (after Psex estimates). For the second set of data (genet-level, only unique genets per population were considered for genetic diversity analysis. The proportion of genets (G) found among the individuals sampled (N) was used to estimate genotypic diversity in each population, using the clonal richness (*R*) index, (R = (G − 1)/(N − 1), ranging from 0 (one single clone) to 1 (when all sampling units analyzed were from different genets).

Microsatellite genetic diversity was quantified for each population by estimating allele frequencies, mean standardized allelic richness (Â) adjusted for the minimum genet sample size, standardized number of private alleles (PÂ), Nei’s gene diversity (H_E_), observed heterozygosity (H_O_) and inbreeding coefficients (F_IS_), using GENETIX 4.05^95^. We have interpreted our results in the context of previous theoretical studies, with a particular focus on the distribution of F_IS_ values among loci and populations for both datasets. By comparing both, we aim to assess whether the clonal rates in populations result in differences in F_IS_ values^96,97^. Also, by examining the distribution of F_IS_ values per locus per site, it may be possible to understand the extent to which asexual and sexual reproduction may be involved at a particular location. A factorial correspondence analysis (FCA) was performed to determine the genetic relationship between populations using GENETIX 4.05. To estimate the distribution of genets among genetic groups that minimize Hardy–Weinberg and linkage disequilibrium, a Bayesian assignment of genotypes to K groups was made using the program STRUCTURE^98^. The value K was estimated from the mean log-likelihood for each *K* value and the Δ*K* statistic^99^ to identify the optimal number of populations groups. This was analyzed for *K* ranging from 2 to 23 with ten replicates per value and a 50,000 burn-in followed by 500,000 MCMC replicates per iteration. All runs were performed in parallel on multiple cores using the R package ‘ParallelStructure’^100^. A discriminant analysis of principal components (DAPC) and a Principal component analysis (PCA) implemented in the R package adegenet 2.1.10^101^ were used to complement structure analysis. Genetic differentiation was estimated between sites (FST).

### Biophysical modelling

Biophysical modeling based on simulations of propagule dispersion coupled with network analysis was used to estimate the connectivity potential of *H. wrightii*. The simulations used daily data of ocean currents assembled from the Hybrid Coordinate Ocean Model (HYCOM), a hindcast of high-resolution three-dimensional ocean velocity fields (regular 1/12-degree horizontal grid with 40 depth layers). This model can resolve key oceanographic processes such as oceanic fronts, eddies, meandering currents, and filaments because it integrates the effect of precipitation, wind stress, wind speed and heat. When hindcast data on ocean current direction and intensity is combined with biological features of pelagic viable time in biophysical modelling, the component of connectivity that is exclusively mediated by ocean currents can be predicted. This modelling approach was previously validated using demographic and genetic data for macroalgae, seagrasses, limpets, mussels, fish, echinoderms, and crustaceans^102–104^.

Individual virtual particles were released on a daily basis (matching HYCOM temporal resolution) from source / sink sites, where the species is known to occur, spaced 1 km apart (spatial resolution of the simulation) over the course of a year. These particles simulate *H. wrightii* rafts strictly transported on the ocean surface, and as in other studies at large spatial scales^102–104^ no shape or density of rafting fragments is considered. Occurrence records describing the species distribution were compiled from the literature and biodiversity information facilities (OBIS and GBIF). The simulation encompassed locations distributed along the east and west Atlantic coastal shores. On the east Atlantic, the simulation included the west coast of Africa from Angola to Mauritania (~ 6250 km of coastlines from -10.0° to 21.5° latitude) and on the west Atlantic, from Florida to south Brazil (~ 13,000 km of coastlines from −30° to 28° latitude). A high-resolution polygon was used to define landmasses^105^. Every hour of simulation, the model determined the position of all drifting rafts while fitting a bilinear interpolation estimate over the velocity fields to smooth ongoing trajectories. Rafts were permitted to float for up to 60 days (extreme propagule duration estimate). Rafts that arrived at a coastline or got lost in the open ocean (outside of the model domain) were excluded from the simulation. Trajectories were aggregated to build asymmetric matrices of pairwise probability of connectivity between source/sink sites, by dividing the number of virtual rafts released from site *i* that reached site *j*, by the total number of rafts released from site *i*. For the ten-year period 2008–2017, interannual variability was analysed by running individual simulations per year. The overall approach of the biophysical modelling is documented in^106^ and the source code is openly available at: http://github.com/jorgeassis/biophysicalModelling.

Graph theory was used to generate networks that allow the visualization of connectivity patterns. The graph nodes (individual source / sink locations) and the strength of edges (probability of connectivity) were structured using a connectivity matrix based on a 10-year average of individual simulations. Stepping-stone probability estimates were determined using the graph, by using a product function over the probabilities of connectivity along the shortest paths between all pairs of sites, as found with Floyd–Warshall’s algorithm, which minimises the sum of log‐transformed probabilities. This approach aimed to capture all multigenerational potential connectivity across the study region, beyond the time frame considered in the biophysical simulations^102–104,107^. Graph analyses were performed in R^92^, using the package igraph.

## Supplementary Information

Supplementary Information.

## Data Availability

The datasets presented in this are openly available in Figshare at: https://figshare.com/s/3ed8c460b0f71559184a.
