# Author Correction: Long range gene flow beyond predictions from oceanographic transport in a tropical marine foundation species

**DOI:** 10.1038/s41598-023-37583-2

**Published:** 2023-07-04

**Authors:** Ana I. Tavares, Jorge Assis, Patrick D. Larkin, Joel C. Creed, Karine Magalhães, Paulo Horta, Aschwin Engelen, Noelo Cardoso, Castro Barbosa, Samuel Pontes, Aissa Regalla, Carmen Almada, Rogério Ferreira, Ba Mamadou Abdoul, Sidina Ebaye, Mohammed Bourweiss, Carmen Van-Dúnem dos Santos, Ana R. Patrício, Alexandra Teodósio, Rui Santos, Gareth A. Pearson, Ester A. Serrao

**Affiliations:** 1 Center of Marine Sciences (CCMAR-CIMAR), Universidade do Algarve, Faro, Portugal; 2 Faculty of Bioscience and Aquaculture, Nord Universitet, Postboks 1490, 8049 Bodø, Norway; 3 Texas A&M University-Corpus Christi, Corpus Christi, TX USA; 4 Departamento de Ecologia, Universidade do Estado do Rio de Janeiro, Rio de Janeiro, RJ Brazil; 5 Área de Ecologia, Departamento de Biologia, Universidade Federal Rural de Pernambuco, R. Dom Manoel de Medeiros, s/n-Dois Irmãos, Recife, PE CEP 52171-900 Brazil; 6 Laboratório de Ficologia, Departamento de Botânica, Universidade Federal de Santa Catarina, Florianópolis, SC 88040-970 Brazil; 7 CARMABI Foundation, Piscaderabaai z/n, P.O. Box 2090, Willemstad, Curaçao The Netherlands; 8 CIPA, Centro de Investigação Pesqueira Aplicada, Bissau, Guinea-Bissau; 9 IBAP-Instituto da Biodiversidade e Áreas Protegidas, Bissau, Guinea-Bissau; 10 Faculdade de Ciências e Tecnologia, Universidade de Cabo Verde, Praia, Cabo Verde; 11 Dragões do Mar, Nova Estrela, Ilha do Príncipe, São Tomé and Príncipe; 12 Parc Nationale du Banc d’Arguin (PNBA), Chami, Mauritania; 13 Institut Mauritanien de Recherche Oceanographique et des Peches (IMROP), Nouadhibou, Mauritania; 14 Universidade do Namibe, Namibe, Angola; 15 MARE-Marine and Environmental Sciences Centre, ISPA-Instituto Universitário, Lisbon, Portugal; 16 Centre for Ecology and Conservation, University of Exete, Penryn, UK; 17 CIBIO-InBIO, Centro de Investigação em Biodiversidade e Recursos Genéticos, Vairão, Portugal

Correction to: *Scientific Reports* https://doi.org/10.1038/s41598-023-36367-y, published online 05 June 2023

The original version of this Article contained an error in the Discussion section, under the subheading ‘Fine scale connectivity’,

“Sea turtles and dugongs are known to feed on *H. wrightii* meadows in these regions^63^, and for some species, these areas are part of their migratory pathways^64^.”

now reads:

“Sea turtles and manatees are known to feed on *H. wrightii* meadows in these regions^63^, and for some species, these areas are part of their migratory pathways^64^.”

The original Article has been corrected.

