# Supplementary material for: Long range gene flow beyond predictions from oceanographic transport in a tropical marine foundation species
Source: Sci Rep. 2023 Jun 5;13:9112. doi: 10.1038/s41598-023-36367-y (PMC10241777; doi:10.1038/s41598-023-36367-y)
Supplement: Supplementary file 1 — Supplementary Information. [file 41598_2023_36367_MOESM1_ESM.doc]

Supplementary material

Table S1 - Geographic location and genetic diversity indices for sampled populations of *Halodule wrightii* (genet-level)*.*Code (#); Population; Location; Country; sample size (N); number of unique genotypes (G); genotypic richness (R); standardized allelic richness (Â); standardized number of private alleles (PÂ) for a common sample size of 2 individuals; gene diversity or expected heterozygosity (HE); observed heterozygosity (HO); and multi-locus inbreeding coefficient (F_IS_) (*=non-significant, p>0.05).

|  | **#** | **Population** | **Location** | **Country** | **N** | **G** | **R** | **A** | **Â _(2)_** | **PÂ _(2)_** | **H_E_** | **H_O_** | **F_IS_** |
| --- | --- | --- | --- | --- | --- | --- | --- | --- | --- | --- | --- | --- | --- |
|  | 1 | TE.CC | Corpus Christi | Texas | 27 | 12 | 0.42 | 4.63 | 2.48 ± 0.33 | 8.44 ± 1.84 | 0.68 | 0.67 | 0.02* |
|  | 2 | TE.PM | Port Mansfield |  | 28 | 26 | 0.93 | 3.88 | 1.92 ± 0.21 | 6.64 ± 1.84 | 0.46 | 0.38 | 0.18* |
| ***Mean (Gulf of Mexico)*** |  | | | |  |  | *0.68* | *4.26* | *2.2±0.27* | *7.54±1.84* | *0.57* | *0.53* | *0.10* |
|  | 3 | BQ.AQ | Aquarium site | St. Eustatius, Caribbean Netherlands | 29 | 7 | 0.21 | 2.13 | 1.47 ± 0.2 | 0.57 ± 0.86 | 0.23 | 0.32 | -0.38 |
|  | 4 | BQ.OB | Oranjested Bay |  | 30 | 12 | 0.38 | 1.75 | 1.39 ± 0.15 | 0.06 ± 0.26 | 0.20 | 0.28 | -0.38 |
|  | 5 | BQ.WW | White Wall |  | 18 | 3 | 0.12 | 1.50 | 1.29 ± 0.16 | 0.09 ± 0.31 | 0.15 | 0.21 | -0.47 |
|  | 6 | CW.BA | Boka Ascension | Curaçao | 28 | 7 | 0.22 | 1.50 | 1.42 ± 0.06 | 0.14 ± 0.34 | 0.21 | 0.33 | -0.56 |
|  | 7 | CW.SM | St. Michiel |  | 37 | 4 | 0.08 | 1.38 | 1.38 ± 0.00 | 1.00 ± 0.00 | 0.19 | 0.35 | -0.84 |
|  | 8 | CW.SW | Spanish Water Entrance |  | 27 | 5 | 0.15 | 1.50 | 1.4 ± 0.07 | 0.09 ± 0.28 | 0.20 | 0.31 | -0.56 |
|  | 9 | BR.FN | Fernando de Noronha | Brazil | 44 | 8 | 0.16 | 2.00 | 1.33 ± 0.14 | 0.56 ± 0.88 | 0.17 | 0.22 | -0.37 |
|  | 10 | BR.OLI | Olinda |  | 23 | 17 | 0.73 | 2.13 | 1.69 ± 0.17 | 0.75 ± 0.63 | 0.35 | 0.38 | -0.08* |
|  | 11 | BR.C | Central Brazil |  | 10 | 8 | 0.78 | 2.13 | 1.65 ± 0.13 | 1.58 ± 0.80 | 0.34 | 0.46 | -0.38 |
|  | 12 | BR.LC | Lagoa da Conceição |  | 11 | 2 | 0.10 | 1.37 | 1.3 ± 0.06 | 0.41 ± 0.49 | 0.16 | 0.28 | -0.84 |
| ***Mean (West Atlantic)*** |  | | | |  |  | *0.19* | *1.63* | *1.40±0.14* | *0.49±0.42* | *0.20* | *0.32* | *-0.43* |
|  | 13 | MR | Banc d’Arguin | Mauritania | 24 | 10 | 0.39 | 2.13 | 1.56 ± 0.10 | 0.3 ± 0.49 | 0.28 | 0.44 | -0.56 |
|  | 14 | SN | Joal-Fadiouth | Senegal | 30 | 8 | 0.24 | 1.63 | 1.32 ± 0.13 | 0.22 ± 0.41 | 0.16 | 0.24 | -0.49 |
|  | 15 | CV | Santiago Island | Cabo Verde | 21 | 9 | 0.40 | 1.63 | 1.39 ± 0.12 | 0.59 ± 0.49 | 0.20 | 0.29 | -0.48 |
|  | 16 | GNB | Unhocomo Island | Guiné-Bissau | 26 | 14 | 0.52 | 2.50 | 1.44 ± 0.18 | 1.06 ± 0.73 | 0.21 | 0.29 | -0.35 |
|  | 17 | STP | São Tomé e Príncipe | São Tomé e Príncipe | 14 | 12 | 0.85 | 2.38 | 1.69 ± 0.11 | 0.71 ± 0.73 | 0.34 | 0.47 | -0.40 |
|  | 18 | GB/STP | Corisco Bay /Santana | Gabon/São Tomé e Principe | 9 | 8 | 0.88 | 2.00 | 1.61 ± 0.14 | 0.42 ± 0.49 | 0.32 | 0.39 | -0.25 |
|  | 19 | AO | Mussulo Bay | Angola | 39 | 4 | 0.08 | 1.75 | 1.51 ± 0.13 | 0.01 ± 0.08 | 0.26 | 0.43 | -0.69 |
| ***Mean (West Africa)*** |  | | | |  |  | *0.40* | *2.00* | *1.51±0.13* | *0.42±0.49* | *0.26* | *0.39* | *-0.46* |
| ***Total*** |  | | | | *475* | *176* |  |  |  |  |  |  |  |

Table S2 - Geographic location and genetic diversity indices for sampled populations of *Halodule wrightii* (ramet-level)*.*Code (#); Population; Location; Country; sample size (N); number of unique genotypes (G); genotypic richness (R); standardized allelic richness (Â); standardized number of private alleles (PÂ) for a common sample size of 2 individuals; gene diversity or expected heterozygosity (HE); observed heterozygosity (HO); and multi-locus inbreeding coefficient (F_IS_) (*=non-significant, p>0.05).

|  | **#** | **Population** | **Location** | **Country** | **N** | **G** | **R** | **A** | **Â _(2)_** | **PÂ _(2)_** | **H_E_** | **H_O_** | **F_IS_** |
| --- | --- | --- | --- | --- | --- | --- | --- | --- | --- | --- | --- | --- | --- |
|  | 1 | TE.CC | Corpus Christi | Texas | 27 | 12 | 0.42 | 4.63 | 2.43 ± 0.4 | 8.66 ± 2.20 | 0.65 | 0.68 | -0.04* |
|  | 2 | TE.PM | Port Mansfield |  | 28 | 26 | 0.93 | 3.88 | 1.92 ± 0.22 | 6.73 ± 1.73 | 0.46 | 0.38 | 0.17* |
| ***Mean (Gulf of Mexico)*** |  | | | |  |  | *0.68* | *4.26* | *2.12 ± 0.31* | *7.70 ± 1.97* | *0.56* | *0.53* | *0.07* |
|  | 3 | BQ.AQ | Aquarium site | St. Eustatius, Caribbean Netherlands | 29 | 7 | 0.21 | 2.13 | 1.32 ± 0.21 | 0.44 ± 0.76 | 0.16 | 0.22 | -0.41 |
|  | 4 | BQ.OB | Oranjested Bay |  | 30 | 12 | 0.38 | 1.75 | 1.35 ± 0.15 | 0.17 ± 0.43 | 0.18 | 0.25 | -0.38 |
|  | 5 | BQ.WW | White Wall |  | 18 | 3 | 0.12 | 1.50 | 1.2 ± 0.13 | 0.09 ± 0.33 | 0.098 | 0.16 | -0.66 |
|  | 6 | CW.BA | Boka Ascension | Curaçao | 28 | 7 | 0.22 | 1.50 | 1.42 ± 0.06 | 0.03 ± 0.16 | 0.21 | 0.34 | -0.66 |
|  | 7 | CW.SM | St. Michiel |  | 37 | 4 | 0.08 | 1.38 | 1.38 ± 0.00 | 1.00 ± 0.00 | 0.19 | 0.36 | -0.94 |
|  | 8 | CW.SW | Spanish Water Entrance |  | 27 | 5 | 0.15 | 1.50 | 1.4 ± 0.05 | 0.08 ± 0.27 | 0.20 | 0.35 | -0.78 |
|  | 9 | BR.FN | Fernando de Noronha | Brazil | 44 | 8 | 0.16 | 2.00 | 1.34 ± 0.13 | 0.54 ± 0.83 | 0.17 | 0.24 | -0.43 |
|  | 10 | BR.OLI | Olinda |  | 23 | 17 | 0.73 | 2.13 | 1.68 ± 0.18 | 0.74 ± 0.66 | 0.35 | 0.38 | -0.07* |
|  | 11 | BR.C | Central Brazil |  | 10 | 8 | 0.78 | 2.13 | 1.65 ± 0.13 | 1.59 ± 0.81 | 0.34 | 0.46 | -0.38 |
|  | 12 | BR.LC | Lagoa da Conceição |  | 11 | 2 | 0.10 | 1.38 | 1.29 ± 0.06 | 0.35 ± 0.48 | 0.15 | 0.28 | -0.86 |
| ***Mean (West Atlantic)*** |  | | | |  |  | *0.19* | *1,74* | *1.40±0.11* | *0.50 ±0.47* | *0.20* | *0.30* | *-0.56* |
|  | 13 | MR | Banc d’Arguin | Mauritania | 24 | 10 | 0.39 | 2.13 | 1.55 ± 0.09 | 0.23 ± 0.44 | 0.28 | 0.45 | -0.63 |
|  | 14 | SN | Joal-Fadiouth | Senegal | 30 | 8 | 0.24 | 1.63 | 1.32 ± 0.12 | 0.20 ± 0.4 | 0.16 | 0.24 | -0.52 |
|  | 15 | CV | Santiago Island | Cabo Verde | 21 | 9 | 0.40 | 1.63 | 1.4 ± 0.12 | 0.63 ± 0.48 | 0.20 | 0.30 | -0.49 |
|  | 16 | GNB | Unhocomo Island | Guiné-Bissau | 26 | 14 | 0.52 | 2.50 | 1.42 ± 0.17 | 1.11 ± 0.71 | 0.21 | 0.28 | -0.37 |
|  | 17 | STP | São Tomé e Príncipe | São Tomé e Príncipe | 14 | 12 | 0.85 | 2.50 | 1.69 ± 0.12 | 0.77 ± 0.74 | 0.35 | 0.46 | -0.32 |
|  | 18 | GB/STP | Corisco Bay /Santana | Gabon/São Tomé e Principe | 9 | 8 | 0.88 | 2.00 | 1.61 ± 0.14 | 0.42 ± 0.49 | 0.32 | 0.39 | -0.25 |
|  | 19 | AO | Mussulo Bay | Angola | 39 | 4 | 0.08 | 1.75 | 1.44 ± 0.10 | 0.00 ± 0.04 | 0.22 | 0.40 | -0.83 |
| ***Mean (West Africa)*** |  | | | |  |  | *0.40* | *2.02* | *1.49±0.12* | *0.48* ± *0.47* | *0.25* | *0.36* | *-0.49* |
| ***Total*** |  | | | | *475* | *176* |  |  |  |  |  |  |  |

Table S3 - Inbreeding coefficient (F_IS_) per population, ramet- level (*=non-significant, p>0.05).

| Population | F_IS_ |
| --- | --- |
| TE.PM | -0.03886* |
| TE.PM | 0.16640* |
| BQ.AQ | -0.40541 |
| BQ.OB | -0.37984 |
| BQ.WW | -0.65678 |
| CW.BA | -0.66019 |
| CW.SM | -0.94497 |
| CW.SW | -0.77920 |
| BR.FN | -0.42670 |
| BR.OLI | -0.07052* |
| BR.C | -0.37888 |
| BR.LC | -0.86047 |
| MR | -0.62617 |
| SN | -0.52355 |
| CV | -0.48810 |
| GNB | -0.36511 |
| STP | -0.32072 |
| GB/STP | -0.25140 |
| AO | -0.83469 |

Table S3 – Single locus inbreeding coefficient (F_IS_) values calculated for the ramet-level dataset for each population (*=non-significant, p>0.05).

| Locus | Population | F_IS_ |
| --- | --- | --- |
| HW 180 | TE.CC | -0.31955 |
|  | TE.PM | -0.08829* |
|  | BQ.AQ | -0.04186* |
|  | BQ.OB | -0.03571* |
|  | BQ.WW | 0.00000* |
|  | BR.FN | -0.17808* |
|  | BR.OLI | -0.10000* |
|  | BR.C | 0.04255* |
|  | MR | -0.05143* |
|  | CV | -0.21212* |
|  | GNB | -0.02041* |
|  | STP | -0.00000* |
|  | AO | -0.01333* |
| HW 188 | TE.CC | 0.31579* |
|  | TE.PM | 0.30591* |
|  | BQ.AQ | -0.16667* |
|  | BQ.OB | -0.09434* |
|  | BQ.WW | -0.03030* |
|  | CW.BA | -0.85224 |
|  | CW.SM | -0.88906 |
|  | CW.SW | -0.84659 |
|  | BR.FN | 0.24138* |
|  | BR.OLI | 0.00000* |
|  | BR.C | -0.05263* |
|  | MR | 0.00000* |
|  | STP | -0.12069* |
|  | GB/STP | 0.58974* |
|  | AO | -1.00000 |
| HW 190 | TE.CC | 0.22139* |
|  | TE.PM | 0.08280* |
|  | BQ.AQ | -1.00000 |
|  | BQ.OB | -1.00000 |
|  | BQ.WW | -1.00000 |
|  | CW.BA | -1.00000 |
|  | CW.SM | -0.94595 |
|  | CW.SW | -1.00000 |
|  | BR.FN | -0.95455 |
|  | BR.OLI | -0.60127 |
|  | BR.C | -1.00000 |
|  | BR.LC | -1.00000 |
|  | MR | -1.00000 |
|  | SN | -0.93333 |
|  | CV | -0.81818 |
|  | GNB | -1.00000 |
|  | STP | -1.00000 |
|  | GB/STP | -1.00000 |
|  | AO | -1.00000 |
| HW 196 | TE.CC | -0.62500 |
|  | TE.PM | 0.00000* |
|  | BQ.AQ | 0.00000* |
|  | BQ.OB | -0.09434* |
|  | BQ.WW | -0.03030* |
|  | BR.FN | -0.06679* |
|  | BR.OLI | -0.46667 |
|  | BR.C | 0.00000* |
|  | MR | -0.32374 |
|  | SN | -0.01754* |
|  | CV | -0.08108* |
|  | GNB | 0.00568* |
|  | STP | -0.02463* |
|  | GB/STP | -0.06667* |
| HW 200 | TE.PM | 0.24492* |
|  | TE.PM | 0.29493* |
|  | BR.C | -0.05882* |
| HW 212 | TE.PM | -0.15789* |
|  | TE.PM | 0.31646* |
|  | BQ.OB | -0.00578* |
|  | CW.BA | -0.24008 |
|  | CW.SM | -1.00000 |
|  | CW.SW | -0.52163 |
|  | BR.FN | -0.12602* |
|  | BR.OLI | 0.49425* |
|  | BR.C | 1.00000* |
|  | BR.LC | -1.00000 |
|  | MR | 0.00000* |
|  | SN | -0.03571* |
|  | GNB | -0.32743 |
|  | STP | 0.25180* |
|  | GB/STP | -0.47368* |
|  | AO | -0.90000 |
| HW 190b | TE.PM | 0.13028* |
|  | TE.PM | 0.15515* |
|  | BR.OLI | 0.08626* |
|  | BR.C | -0.39806* |
|  | MR | -0.76923 |
|  | SN | -0.09434* |
|  | CV | -0.05263* |
|  | GNB | 0.48718* |
|  | STP | -0.36842* |
|  | GB/STP | -0.14286* |
|  | AO | -0.04110* |
| HW 228 | TE.PM | -0.24121 |
|  | TE.PM | 0.00000* |
|  | BQ.AQ | 0.32121* |
|  | BQ.OB | -0.01754* |
|  | BR.C | -1.00000 |
|  | MR | -0.70370 |
|  | SN | -0.41463 |
|  | CV | -0.60000 |
|  | GNB | -0.06383* |
|  | STP | -0.53763 |
|  | GB/STP | -0.33333* |
|  | AO | -0.04110* |

Table S4 - Geographic location and genetic diversity indices for the first hierarchal level of Structure subdivision (K=3) for *Halodule wrightii.* Cluster; sample size (N); number of unique genotypes (G); genotypic richness (R); standardized allelic richness (Â); standardized number of private alleles (PÂ) for a minimum common sample size of 38 individuals; gene diversity or expected heterozygosity (HE); observed heterozygosity (HO); and multi-locus inbreeding coefficient (F_IS_).

| **Cluster** | **N** | **G** | **R** | **A** | **Â _(38)_** | **PÂ _(38)_** | **H_E_** | **H_O_** | **F_IS_** |
| --- | --- | --- | --- | --- | --- | --- | --- | --- | --- |
| Gulf of Mexico | 55 | 38 | 0.69 | 5.38 | 5.38±0.00 | 34.02±0.64 | 0.58 | 0.47 | 0.19 |
| West Atalntic | 257 | 61 | 0.23 | 5.13 | 4.08±0.27 | 19.11±1.79 | 0.50 | 0.31 | 0.38 |
| West Africa | 163 | 52 | 0.31 | 3.62 | 2.92±0.20 | 9.72±1.46 | 0.27 | 0.34 | -0.27 |

Table S5 – *Halodule wrightii* data from literature, our samples and biodiversity databases

| LAT_WGS84_DEG | LON_WGS84_DEG | Local name | Country | Source/Reference |
| --- | --- | --- | --- | --- |
| 19.739766 | -16.457939 | Banc d'Arguin | Mauritania | Wolff. W. J.. et al. "Biomass of macrobenthic tidal flat fauna of the Banc d'Arguin. Mauritania."Hydrobiologia, 258.1-3 (1993): 151-163. |
| 20.228214 | -16.225236 | Banc d'Arguin | Mauritania | Cardona. Luis. A. Aguilar. and L. Pazos. "Delayed ontogenic dietary shift and high levels of omnivory in green turtles (Chelonia mydas) from the NW coast of Africa."Marine Biology, 156.7 (2009): 1487-1495. |
| 19.48 | -16.445 | Teichott, Banc d’Arguin | Mauritania | Biogeographical Ecology and Evolution Group, CCMAR |
| 14.27166667 | -16.90472222 | Joal Fadiouth | Senegal | Cunha. A. H.. & Araújo. A. (2009). New distribution limits of seagrass beds in West Africa. Journal of Biogeography. 36(8). 1621-1622. doi:10.1111/j.1365-2699.2009.02135.x |
| 14.15222222 | -16.83416667 | Bamboung-Sourou area | Senegal | Cunha. A. H.. & Araújo. A. (2009).New distribution limits of seagrass beds in West Africa. Journal of Biogeography. 36(8). 1621-1622.doi:10.1111/j.1365-2699.2009.02135.x |
| 14.156 | -16.841 | Fadiouth | Senegal | Biogeographical Ecology and Evolution Group, CCMAR |
| 13.384509 | -16.811234 | Bijol Islands | Gambia | https://resiliensea.org/2020/01/27/participant-diary-from-the-national-seagrass-species-identification-mapping-and-monitoring-training-in-the-gambia/ |
| 14.90888889 | -23.51027778 | Santiago Island | Cabo Verde | Creed. Joel C.. et al. "First record of seagrass in Cape Verde. eastern Atlantic."Marine Biodiversity Records, 9.1 (2016): 57. |
| 14.912 | -23.508 | Gamboa, Praia (Santiago) | Cabo Verde | Biogeographical Ecology and Evolution Group, CCMAR |
| 11.281 | -16.382 | Acampamento, Unhocomo | Guinea-Bissau | Biogeographical Ecology and Evolution Group, CCMAR |
| 11.312 | -16.405 | Ancante, Unhocomo | Guinea-Bissau | Biogeographical Ecology and Evolution Group, CCMAR |
| 7.641079 | -13.05448 | Moot Island | Sierra Leone | https://news.grida.no/finding-treasure-in-the-turtle-islands |
| 7.660010 | -13.029801 | Mania | Sierra Leone | ResilienSEA porject |
| 7.660056 | -13.029837 | Mania | Sierra Leone | ResilienSEA porject |
| 7.660111 | -13.029945 | Mania | Sierra Leone | ResilienSEA porject |
| 7.660138 | -13.030000 | Mania | Sierra Leone | ResilienSEA porject |
| 7.660129 | -13.030000 | Mania | Sierra Leone | ResilienSEA porject |
| 7.660111 | -13.030045 | Mania | Sierra Leone | ResilienSEA porject |
| 7.660093 | -13.030045 | Mania | Sierra Leone | ResilienSEA porject |
| 7.660808 | -13.030160 | Mania | Sierra Leone | ResilienSEA porject |
| 7.659867 | -13.030173 | Mania | Sierra Leone | ResilienSEA porject |
| 7.659858 | -13.030182 | Mania | Sierra Leone | ResilienSEA porject |
| 7.659707 | -13.030763 | Mania | Sierra Leone | ResilienSEA porject |
| 7.659471 | -13.030374 | Mania | Sierra Leone | ResilienSEA porject |
| 7.659403 | -13.031480 | Mania | Sierra Leone | ResilienSEA porject |
| 0.414204 | 6.68026 | Cabras islet | São Tomé e Príncipe | Alexandre. Ana. et al. "First description of seagrass distribution and abundance in São Tomé and Príncipe."Aquatic Botany, 142 (2017): 48-52. |
| 0.395462 | 6.730666 | Cabras islet | São Tomé e Príncipe | Alexandre. Ana. et al. "First description of seagrass distribution and abundance in São Tomé and Príncipe."Aquatic Botany, 142 (2017): 48-52. |
| 0.246614 | 6.7465 | Santana Bay | São Tomé e Príncipe | Alexandre. Ana. et al. "First description of seagrass distribution and abundance in São Tomé and Príncipe."Aquatic Botany, 142 (2017): 48-52. |
| 1.632689 | 7.454281 | Abade beach (island of Pri?ncipe) | São Tomé e Príncipe | Alexandre. Ana. et al. "First description of seagrass distribution and abundance in São Tomé and Príncipe."Aquatic Botany, 142 (2017): 48-52. |
| 0.57277778 | 6.86166667 | Ilheu das Cabras | São Tomé e Príncipe | Hancock. Joana M.. et al. "Stable isotopes reveal dietary differences and site fidelity in juvenile green turtles foraging around São Tomé Island. West Central Africa."Marine Ecology Progress Series, 600 (2018): 165-177. |
| 1.633 | 7.455 | Abade beach, Pagué, Príncipe | São Tomé e Príncipe | Biogeographical Ecology and Evolution Group, CCMAR |
| 1.635 | 7.456 | Abade beach, Pagué, Príncipe | São Tomé e Príncipe | Biogeographical Ecology and Evolution Group, CCMAR |
| 0.414 | 6.656 | Cabras Islet | São Tomé e Príncipe | Biogeographical Ecology and Evolution Group, CCMAR |
| 0.255 | 6.747 | Santana | São Tomé e Príncipe | Biogeographical Ecology and Evolution Group, CCMAR |
| 0.892721 | 9.349402 | Corisco Bay | Gabon | http://www.yaqupacha.org/fileadmin/user_upload/pdf/seagrass_magazine_46_2012.pdf |
| 0.594 | 9.307 | Corisco Bay | Gabon | Biogeographical Ecology and Evolution Group, CCMAR |
| -9.006 | 13.064 | Mussulo Bay | Angola | Biogeographical Ecology and Evolution Group, CCMAR |
| -8.95653 | 13.108091 | Ba¡a do Mussulo | Angola | Santos. Carmen I. "Comunidades de macroinvertebrados e peixes associadas … pradaria marinha de Halodule wrightii (Ascherson. 1868) na Laguna do Mussulo. Angola." (2007). |
| 27.718 | -97.324 | Corpus Christi, Texas | United States | Biogeographical Ecology and Evolution Group, CCMAR |
| 26.319 | -97.223 | Port Mansfield, Texas | United States | Biogeographical Ecology and Evolution Group, CCMAR |
| 34.71666667 | -76.66666667 | Beaufort, North carolina | United States | Biber. Patrick D.. W. Judson Kenworthy. and Hans W. Paerl. "Experimental analysis of the response and recovery of Zostera marina (L.) and Halodule wrightii (Ascher.) to repeated light-limitation stress."Journal of Experimental Marine Biology and Ecology369.2 (2009): 110-117. |
| 27.85333333 | -80.49138889 | Indian River Lagoon south of Sebastian River, Florida | United States | Hall. Lauren M.. M. Dennis Hanisak. and Robert W. Virnstein. "Fragments of the seagrasses Halodule wrightii and Halophila johnsonii as potential recruits in Indian River Lagoon. Florida."Marine Ecology Progress Series310 (2006): 109-117. |
| 30.38333333 | -87.4 | Big Lagoon. Perdido Bay, Florida | United States | Heck Jr. K. L.. et al. "Effects of nutrient enrichment and grazing on shoalgrass Halodule wrightii and its epiphytes: results of a field experiment."Marine Ecology Progress Series326 (2006): 145-156. |
| 28.34527778 | -80.70361111 | Indian River Lagoon , Florida | United States | Taplin. K. A.. Irlandi. E. A.. & Raves. R. (2005).Interference between the macroalga Caulerpa prolifera and the seagrass Halodule wrightii. Aquatic Botany. 83(3). 175-186.doi:10.1016/j.aquabot.2005.06.003 |
| 28.10833333 | -82.78611111 | Indian Bluff Island, Florida | United States | Dawes. C. J.. & Lawrence. J. M. (1980).Seasonal changes in the proximate constituents of the seagrasses Thalassia testudinum. Halodule wrightii. and Syringodium filiforme. Aquatic Botany. 8. 371-380.doi:10.1016/0304-3770(80)90066-2 |
| 30.3 | -87.5 | Perdido Bay, Alabama | United States | Shafer. Deborah J. "The effects of dock shading on the seagrass Halodule wrightii in Perdido Bay. Alabama."Estuaries22.4 (1999): 936-943. |
| 27.35 | -97.36666667 | Laguna Madre, Texas | United States | Burd. Adrian B.. and K. H. Dunton. "Field verification of a light-driven model of biomass changes in the seagrass Halodule wrightii."Marine Ecology Progress Series209 (2001): 85-98. |
| 26.15 | -97.23416667 | Gulf Intra- coastal Waterway, Texas | United States | Kowalski. Joseph L.. et al. "Productivity estimation in Halodule wrightii Aschers: comparison of leaf-clipping and leaf-marking techniques and the importance of clip height."Marine Ecology Progress Series220 (2001): 131-136. |
| 26.06666667 | -97.15 | Isla Blanca Park. South Padre Island, Texas | United States | Kowalski. Joseph L.. and Hudson R. DeYoe. "Flowering and seed production in the subtropical seagrass. Halodule wrightii (shoal grass)."Botanica marina59.2-3 (2016): 193-199. |
| 18.620293 | -91.787836 | Laguna de Términos | Mexico | Coria-Monter. Erik. and Elizabeth Dur n-Campos. "Análisis proximal de los pastos marinos de la Laguna de Términos, México."Hidrobiológica, 25.2 (2015): 249-255. |
| 24.50416667 | -97.74055556 | Punta Carbajal | Mexico | Rodr¡guez-Almaraz. Gabino A.. and Víctor M. Ortega-Vidales. "Primer registro de Caprella scaura y Caprella penantis (Crustacea: Peracarida: Amphipoda) en la laguna Madre. Tamaulipas. México."Revista mexicana de biodiversidad84.3 (2013): 989-993. |
| 19.589034 | -96.381382 | Laguna de la Mancha | Mexico | del Pilar Reyes-Barrag n. Ma. and Sergio I. Salazar-Vallejo. "Bentos asociado al pastizal de Halodule (Potamogetonaceae) en Laguna de la Mancha. Veracruz. México."Revista de Biolog¡a Tropical(1990): 167-173. |
| 11.945213 | -66.676877 | Gran Roque | Venezuela | Burandt. C. L.. & Campins. R. D. (1986).Colonization. Extinction and Species Numbers of Vascular Plants for the Island Gran Roque. Venezuela. Journal of Biogeography. 13(6). 541.doi:10.2307/2844817 |
| 12.01416667 | -69.83305556 | El Supí | Venezuela | Mariño, Joany, María Daniela Mendoza, and Beatriz López-Sánchez. "Composition and abundance of decapod crustaceans in mixed seagrass meadows in the Paraguaná Peninsula, Venezuela." Iheringia. Série Zoologia 108 (2018). |
| 10.617109 | -66.749138 | Puerto Azúl Bay (Playa Mansa) | Venezuela | Vera, Beatriz, et al. "Halophila stipulacea (Hydrocharitaceae): a recent introduction to the continental waters of Venezuela." Caribbean Journal of Science 48.1 (2014): 66-70. |
| 25.73333333 | -79.26666667 | Bimini | Bahamas | Fuentes. Mariana MPB. et al. "Informing marine protected areas in Bimini. Bahamas by considering hotspots for green turtles (Chelonia mydas)."Biodiversity and conservation28.1 (2019): 197-211. |
| 17.7575 | -64.59638889 | St. Croix, Virgin Islands | United States | Feser. Kelsey M.Utilizing the Subfossil Record of Seagrass-Associated Mollusks to Reveal Recent Changes in Coastal Marine Environments. Diss. University of Cincinnati. 2015. |
| 12.273369 | -69.052366 | Ascension | Curaçao | Stuij. T. M.Distinct Microbiomes in three Tropical seagrasses around the island of Curaçao: Halophila stipulacea. Halodule wrightii and Thalassia testudinum. MS thesis. 2018. |
| 12.204255 | -69.052609 | Jankok | Curaçao | Stuij. T. M.Distinct Microbiomes in three Tropical seagrasses around the island of Curaçao: Halophila stipulacea. Halodule wrightii and Thalassia testudinum. MS thesis. 2018. |
| 12.066101 | -68.853585 | Spanish Water | Curaçao | Stuij. T. M.Distinct Microbiomes in three Tropical seagrasses around the island of Curaçao: Halophila stipulacea. Halodule wrightii and Thalassia testudinum. MS thesis. 2018. |
| 12.272 | -69.055 | Boka ascension | Curaçao | Biogeographical Ecology and Evolution Group, CCMAR |
| 12.148 | -68.998 | St. Michiel | Curaçao | Biogeographical Ecology and Evolution Group, CCMAR |
| 12.066 | -68.854 | Spanish water entrance | Curaçao | Biogeographical Ecology and Evolution Group, CCMAR |
| 17.513 | -63.000 | Aquarium site, St. Eustatius Island | Netherlands | Biogeographical Ecology and Evolution Group, CCMAR |
| 17.483 | -62.988 | Oranjested Bay, St. Eustatius Island | Netherlands | Biogeographical Ecology and Evolution Group, CCMAR |
| 17.465 | -62.956 | White Wall, St. Eustatius Island | Netherlands | Biogeographical Ecology and Evolution Group, CCMAR |
| 10.690153 | -61.746430 | Chacachacare Island, Trindade e Tobago | Republic of Trinidad and Tobago | Juman, Rahanna A., and Karlene James Alexander. "An Inventory of Seagrass Communities around Trinidad and Tobago." (2006). |
| 10.687663 | -61.743271 | Chacachacare Island, Trindade e Tobago | Republic of Trinidad and Tobago | Juman, Rahanna A., and Karlene James Alexander. "An Inventory of Seagrass Communities around Trinidad and Tobago." (2006). |
| 10.684581 | -61.740295 | Chacachacare Island, Trindade e Tobago | Republic of Trinidad and Tobago | Juman, Rahanna A., and Karlene James Alexander. "An Inventory of Seagrass Communities around Trinidad and Tobago." (2006). |
| 10.687684 | -61.687504 | Monos Island, Trindade e Tobago | Republic of Trinidad and Tobago | Juman, Rahanna A., and Karlene James Alexander. "An Inventory of Seagrass Communities around Trinidad and Tobago." (2006). |
| 10.686772 | -61.685994 | Monos Island, Trindade e Tobago | Republic of Trinidad and Tobago | Juman, Rahanna A., and Karlene James Alexander. "An Inventory of Seagrass Communities around Trinidad and Tobago." (2006). |
| 10.697099 | -61.679489 | Monos Island, Trindade e Tobago | Republic of Trinidad and Tobago | Juman, Rahanna A., and Karlene James Alexander. "An Inventory of Seagrass Communities around Trinidad and Tobago." (2006). |
| 10.668788 | -61.646508 | Gaspar Grande Island, Trindade e Tobago | Republic of Trinidad and Tobago | Juman, Rahanna A., and Karlene James Alexander. "An Inventory of Seagrass Communities around Trinidad and Tobago." (2006). |
| 10.662527 | -61.597124 | Caledonia Island,Trindade e Tobago | Republic of Trinidad and Tobago | Juman, Rahanna A., and Karlene James Alexander. "An Inventory of Seagrass Communities around Trinidad and Tobago." (2006). |
| 10.701635 | -61.665601 | Scotland Bay, Trindade e Tobago | Republic of Trinidad and Tobago | Juman, Rahanna A., and Karlene James Alexander. "An Inventory of Seagrass Communities around Trinidad and Tobago." (2006). |
| 10.697588 | -61.664677 | Scotland Bay, Trindade e Tobago | Republic of Trinidad and Tobago | Juman, Rahanna A., and Karlene James Alexander. "An Inventory of Seagrass Communities around Trinidad and Tobago." (2006). |
| 10.690281 | -61.602919 | Carenage Bay, Trindade e Tobago | Republic of Trinidad and Tobago | Juman, Rahanna A., and Karlene James Alexander. "An Inventory of Seagrass Communities around Trinidad and Tobago." (2006). |
| 10.683852 | -61.593131 | Carenage Bay, Trindade e Tobago | Republic of Trinidad and Tobago | Juman, Rahanna A., and Karlene James Alexander. "An Inventory of Seagrass Communities around Trinidad and Tobago." (2006). |
| 10.682546 | -61.584118 | Carenage Bay, Trindade e Tobago | Republic of Trinidad and Tobago | Juman, Rahanna A., and Karlene James Alexander. "An Inventory of Seagrass Communities around Trinidad and Tobago." (2006). |
| 10.336678 | -61.461383 | Claxton Bay, Trindade e Tobago | Republic of Trinidad and Tobago | Juman, Rahanna A., and Karlene James Alexander. "An Inventory of Seagrass Communities around Trinidad and Tobago." (2006). |
| 10.141755 | -61.001047 | Guayaguayare Bay, Trindade e Tobago | Republic of Trinidad and Tobago | Juman, Rahanna A., and Karlene James Alexander. "An Inventory of Seagrass Communities around Trinidad and Tobago." (2006). |
| 11.167222 | -60.826915 | Bon Accord Lagoon, Trindade e Tobago | Republic of Trinidad and Tobago | Juman, Rahanna A., and Karlene James Alexander. "An Inventory of Seagrass Communities around Trinidad and Tobago." (2006). |
| 11.141501 | -60.804047 | Canoe Bay, Trindade e Tobago | Republic of Trinidad and Tobago | Juman, Rahanna A., and Karlene James Alexander. "An Inventory of Seagrass Communities around Trinidad and Tobago." (2006). |
| 11.266350 | -60.546648 | King’s Bay, Trindade e Tobago | Republic of Trinidad and Tobago | Juman, Rahanna A., and Karlene James Alexander. "An Inventory of Seagrass Communities around Trinidad and Tobago." (2006). |
| -22.95138889 | -43.90777778 | Itacurucá Island | Brazil | Filho. G. M. A.. Creed. J. C.. Andrade. L. R.. & Pfeiffer. W. C. (2004).Metal accumulation by Halodule wrightii populations. Aquatic Botany. 80(4). 241-251.doi:10.1016/j.aquabot.2004.07.011 |
| -23.054443 | -43.926944 | Jaguanum Island | Brazil | Filho. G. M. A.. Creed. J. C.. Andrade. L. R.. & Pfeiffer. W. C. (2004).Metal accumulation by Halodule wrightii populations. Aquatic Botany. 80(4). 241-251.doi:10.1016/j.aquabot.2004.07.011 |
| -22.98333 | -44.434444 | Saco de Piraquara | Brazil | Filho, G. M. A., Creed, J. C., Andrade, L. R., & Pfeiffer, W. C. (2004).Metal accumulation by Halodule wrightii populations. Aquatic Botany, 80(4), 241-251.doi:10.1016/j.aquabot.2004.07.011 |
| -22.883052 | -42.011112 | Araruama Lagoon | Brazil | Filho, G. M. A., Creed, J. C., Andrade, L. R., & Pfeiffer, W. C. (2004).Metal accumulation by Halodule wrightii populations. Aquatic Botany, 80(4), 241-251.doi:10.1016/j.aquabot.2004.07.011 |
| -5.104827 | -36.321424 | Guamare | Brazil | Silva, Juliana, et al. "Biological activities of the sulfated polysaccharide from the vascular plant Halodule wrightii."Revista Brasileira de Farmacognosia22.1 (2012): 94-101. |
| -17.96666667 | -38.7175 | Bahia (65 km off the southern coast of Bahia state) | Brazil | de Paula, Alline Figueira, MA de O. Figueiredo, and Joel Christopher Creed. "Structure of the macroalgal community associated with the seagrass Halodule wrightii Ascherson in the Abrolhos Marine National Park, Brazil."Botanica Marina46.5 (2003): 413-424. |
| -3.69194444 | -38.58027778 | Goiabeiras Beach | Brazil | Barros, Kcrishna VS, and CRISTINA A. ROCHA-BARREIRA. "Responses of the molluscan fauna to environmental variations in a Halodule wrightii Ascherson ecosystem from Northeastern Brazil."Anais da Academia Brasileira de Ciˆncias85.4 (2013): 1397-1410. |
| -12.83333333 | -38.63333333 | Todos os Santos Bay, Bahia | Brazil | Brito, G. B., de Souza, T. L., do N. Costa, F., Moura, C. W. N., & Korn, M. G. A. (2016).Baseline trace elements in the seagrass Halodule wrightii Aschers (Cymodoceaceae) from Todos os Santos Bay, Bahia, Brazil. Marine Pollution Bulletin, 104(1-2), 335-342.doi:10.1016/j.marpolbul.2016.01.044 |
| -7.72944444 | -34.82555556 | Jaguaribe, Itamaracá Island | Brazil | PITANGA, MARIA ELISA, et al. "Quantification and classification of the main environmental impacts on a Halodule wrightii seagrass meadow on a tropical island in northeastern Brazil."An Acad Bras Cienc84.1 (2012). |
| -7.75333333 | -34.82361111 | Pilar, Itamaracá Island | Brazil | PITANGA, MARIA ELISA, et al. "Quantification and classification of the main environmental impacts on a Halodule wrightii seagrass meadow on a tropical island in northeastern Brazil."An Acad Bras Cienc84.1 (2012). |
| -2.90194444 | -41.40944444 | Barra Grande Beach, in Piauí State | Brazil | Cavalcante, L. L., Barroso, C. X., Carneiro, P. B. de M., & Matthews-Cascon, H. (2019).Spatiotemporal dynamics of the molluscan community associated with seagrass on the western equatorial Atlantic. Journal of the Marine Biological Association of the United Kingdom, 1-10.doi:10.1017/s0025315419000183 |
| -17.97722222 | -38.71666667 | Abrolhos archipelago | Brazil | Creed, Joel C., and Gilberto M. Amado Filho. "Disturbance and recovery of the macroflora of a seagrass (Halodule wrightii Ascherson) meadow in the Abrolhos Marine National Park, Brazil: an experimental evaluation of anchor damage."Journal of experimental marine biology and ecology235.2 (1999): 285-306. |
| -23.5 | -45.11666667 | Saco da Ribeira, São Paulo | Brazil | Corbisier, Thais Navajas. "Macrozoobentos da Praia do Cod¢ (Ubatuba, SP) e a presen‡a de Halodule wrightii Ascherson."Boletim do Instituto Oceanogr fico42.1-2 (1994): 99-111. |
| -22.769548 | -41.884128 | Buzios, Rio de Janeiro | Brazil | Omena, Elianne, and Joel C. Creed. "Polychaete fauna of seagrass beds (Halodule wrightii Ascherson) along the coast of Rio de Janeiro (Southeast Brazil)."Marine Ecology25.4 (2004): 273-288. |
| -22.882278 | -42.005635 | Cabo Frio, Rio do Janeiro | Brazil | Omena, Elianne, and Joel C. Creed. "Polychaete fauna of seagrass beds (Halodule wrightii Ascherson) along the coast of Rio de Janeiro (Southeast Brazil)."Marine Ecology25.4 (2004): 273-288. |
| -22.996072 | -43.917471 | Jaguanum Island | Brazil | Omena, Elianne, and Joel C. Creed. "Polychaete fauna of seagrass beds (Halodule wrightii Ascherson) along the coast of Rio de Janeiro (Southeast Brazil)."Marine Ecology25.4 (2004): 273-288. |
| -22.948193 | -43.908769 | Itacuruçá Island | Brazil | Omena, Elianne, and Joel C. Creed. "Polychaete fauna of seagrass beds (Halodule wrightii Ascherson) along the coast of Rio de Janeiro (Southeast Brazil)."Marine Ecology25.4 (2004): 273-288. |
| -23.016326 | -44.226415 | Monsuaba | Brazil | Omena, Elianne, and Joel C. Creed. "Polychaete fauna of seagrass beds (Halodule wrightii Ascherson) along the coast of Rio de Janeiro (Southeast Brazil)."Marine Ecology25.4 (2004): 273-288. |
| -23.215295 | -44.619266 | Parati | Brazil | Omena, Elianne, and Joel C. Creed. "Polychaete fauna of seagrass beds (Halodule wrightii Ascherson) along the coast of Rio de Janeiro (Southeast Brazil)."Marine Ecology25.4 (2004): 273-288. |
| -16.40777778 | -38.99305556 | Parque Municipal Marinho do Recife de Fora | Brazil | Schneider, Geniane. " Efeitos do aumento de CO² na fisiologia, anatomia e ultraestrutura de Halodule wrightii Ascherson" (2014). |
| -25.45238 | -48.443468 | Baixio do Perigo, Paran | Brazil | Leis, Mirella de Oliveira. "Interação entre o ambiente sedimentar e pradarias de Halodule wrightii do Complexo Estuarino de Paranaguá-PR." |
| -25.539571 | -48.308123 | Saco do Limoeiro, Paran | Brazil | Leis, Mirella de Oliveira. "Interação entre o ambiente sedimentar e pradarias de Halodule wrightii do Complexo Estuarino de Paranaguá-PR." |
| -3.692549 | -38.584539 | Goiabeiras beach, Fortaleza | Brazil | Barros, Kcrishna VS, et al. "Seasonal variation of the crustacean fauna in the belowground and aboveground strata in a Halodule wrightii meadow of northeastern Brazil." Iheringia. Série Zoologia 107 (2017). |
| -4.68333333 | -37.35 | Icapuí | Brasil | Barros, Kcrishna VS, et al. "Seasonal variation of the crustacean fauna in the belowground and aboveground strata in a Halodule wrightii meadow of northeastern Brazil." Iheringia. Série Zoologia 107 (2017). |
| -3.867 | -32.424 | Fernando Noronha | Brazil | Biogeographical Ecology and Evolution Group, CCMAR |
| -7.981 | -34.834 | Olinda | Brazil | Biogeographical Ecology and Evolution Group, CCMAR |
| -19.911950 | -40.097230 | Praia Mar Azul, Aracruz | Brazil | Biogeographical Ecology and Evolution Group, CCMAR |
| -19.953790 | -40.141317 | Praia de Santa Cruz II, Aracruz | Brazil | Biogeographical Ecology and Evolution Group, CCMAR |
| -20.037988 | -40.167251 | Praia Grande, Fundão | Brazil | Biogeographical Ecology and Evolution Group, CCMAR |
| -20.096035 | -40.173115 | Praia de Costa Bela, Serra | Brazil | Biogeographical Ecology and Evolution Group, CCMAR |
| -20.119948 | -40.175779 | Praia de Jacaraípe III, Serra | Brazil | Biogeographical Ecology and Evolution Group, CCMAR |
| -20.200215 | -40.192379 | Praia de Manguinhos III, Serra | Brazil | Biogeographical Ecology and Evolution Group, CCMAR |
| -19.972685 | -40.137331 | Praia do Mamão, Aracruz | Brazil | Biogeographical Ecology and Evolution Group, CCMAR |
| -20.301384 | -40.285798 | Ilha do Frade, Vitória | Brazil | Biogeographical Ecology and Evolution Group, CCMAR |
| -20.324801 | -40.271780 | Praia do Ribeiro, Vila Velha | Brazil | Biogeographical Ecology and Evolution Group, CCMAR |
| -20.309373 | -40.283671 | lha do Boi, Vitória | Brazil | Biogeographical Ecology and Evolution Group, CCMAR |
| -27.581 | -48.446 | Lagoa da Conceição | Brazil | Biogeographical Ecology and Evolution Group, CCMAR |
| -2.966595 | -39.749809 | Almofala | Brazil | https://research.unl.pt/ws/portalfiles/portal/3337967/Livro_Braspor_2015_SFRH_BPD_70384_2010_UID_ELT_00657_2013_.pdf |
| -2.966633 | -39.751758 | Almofala | Brazil | https://research.unl.pt/ws/portalfiles/portal/3337967/Livro_Braspor_2015_SFRH_BPD_70384_2010_UID_ELT_00657_2013_.pdf |
| -2.953404 | -39.765741 | Almofala | Brazil | https://research.unl.pt/ws/portalfiles/portal/3337967/Livro_Braspor_2015_SFRH_BPD_70384_2010_UID_ELT_00657_2013_.pdf |
| -25.530833 | -48.396389 | Ilha Rasa da Cotinga | Brazil | Guebert, Flávia Maria. "Ecologia alimentar e consumo de material inorgânico por tartarugas-verdes, Chelonia mydas, no litoral do Estado do Paraná." Master's thesis. Universidade Federal do Paraná, Curitiba, PR, Brazil (2008). |
| -25.493611 | -48.340000 | Ilha do Mel | Brazil | Guebert, Flávia Maria. "Ecologia alimentar e consumo de material inorgânico por tartarugas-verdes, Chelonia mydas, no litoral do Estado do Paraná." Master's thesis. Universidade Federal do Paraná, Curitiba, PR, Brazil (2008). |
| -25.471944 | -48.429722 | Ilha das Cobras | Brazil | Guebert, Flávia Maria. "Ecologia alimentar e consumo de material inorgânico por tartarugas-verdes, Chelonia mydas, no litoral do Estado do Paraná." Master's thesis. Universidade Federal do Paraná, Curitiba, PR, Brazil (2008). |
| -6.757994 | -34.929734 | Mamanguape River Estuary | Brazil | da Silva, Rayssa Soares, Alexandra Sofia Baptista Vicente Baeta, and André Luiz Machado Pessanha. "Are vegetated areas more attractive for juvenile fish in estuaries? A comparison in a tropical estuary." Environmental Biology of Fishes 101.10 (2018): 1427-1442. |
| -2.902499 | -41.398788 | Barra Grande | Brazil | Cavalcante, Lorraine Lopes. "Malacofauna associada ao prado de Halodule wrightii Ascherson em Barra Grande, Piauí." (2015). |
| -5.245949 | -35.360025 | Área de Proteção Ambiental dos Recifes de Corais | Brazil | Viana, Marina Gomes. "Macrofauna de ambientes não consolidados adjacentes à recifes da área de proteção ambiental dos recifes de corais Rio Grande do Norte, Brasil." (2013). |
| -2.902222 | -41.570556 | Coqueiro | Brazil | da Silva, Noelia Pereira, et al. "Seagrasses of Piauí, Brazil: A floristic treatment." Feddes Repertorium 129.1 (2018): 43-50. |
| -2.915833 | -41.451389 | Macapá | Brazil | da Silva, Noelia Pereira, et al. "Seagrasses of Piauí, Brazil: A floristic treatment." Feddes Repertorium 129.1 (2018): 43-50. |
| -2.925000 | -41.328056 | Barbaço | Brazil | da Silva, Noelia Pereira, et al. "Seagrasses of Piauí, Brazil: A floristic treatment." Feddes Repertorium 129.1 (2018): 43-50. |
| -2.920000 | '-41.321389 | Croa Grande | Brazil | da Silva, Noelia Pereira, et al. "Seagrasses of Piauí, Brazil: A floristic treatment." Feddes Repertorium 129.1 (2018): 43-50. |
| -2.931944 | -41.317222 | Ilha Grande | Brazil | da Silva, Noelia Pereira, et al. "Seagrasses of Piauí, Brazil: A floristic treatment." Feddes Repertorium 129.1 (2018): 43-50. |
| 25.69821 | -80.154366 |  |  | GBIF |
| 19.328134 | -69.456353 |  |  | GBIF |
| 25.125762 | -80.404862 |  |  | GBIF |
| 19.325133 | -69.48246 |  |  | GBIF |
| 19.323658 | -69.48674 |  |  | GBIF |
| 12.45711 | -61.483208 |  |  | GBIF |
| -13.917472 | -38.936277 |  |  | GBIF |
| -16.403056 | -38.982222 |  |  | GBIF |
| 29.772326 | -83.579375 |  |  | GBIF |
| 29.20775 | -94.95833 |  |  | GBIF |
| -16.407778 | -38.993056 |  |  | GBIF |
| -27.579722 | -48.429722 |  |  | GBIF |
| 25.31388 | -80.30989 |  |  | GBIF |
| 25.31177 | -80.30064 |  |  | GBIF |
| 25.21897 | -80.36522 |  |  | GBIF |
| 25.22878 | -80.37006 |  |  | GBIF |
| 25.32355 | -80.29167 |  |  | GBIF |
| 25.23078 | -80.36703 |  |  | GBIF |
| 25.23381 | -80.35669 |  |  | GBIF |
| 25.2587 | -80.3498 |  |  | GBIF |
| 25.24186 | -80.36237 |  |  | GBIF |
| 25.24494 | -80.34817 |  |  | GBIF |
| 25.21164 | -80.36176 |  |  | GBIF |
| 25.21297 | -80.37633 |  |  | GBIF |
| 25.20694 | -80.37376 |  |  | GBIF |
| 25.06301 | -80.66747 |  |  | GBIF |
| 25.05055 | -80.66206 |  |  | GBIF |
| 25.05788 | -80.68124 |  |  | GBIF |
| 25.07454 | -80.67886 |  |  | GBIF |
| 25.08188 | -80.68774 |  |  | GBIF |
| 25.07971 | -80.68259 |  |  | GBIF |
| 24.99754 | -80.65176 |  |  | GBIF |
| 25.0486 | -80.67296 |  |  | GBIF |
| 25.05158 | -80.68393 |  |  | GBIF |
| 24.99759 | -80.64209 |  |  | GBIF |
| 25.07185 | -80.68289 |  |  | GBIF |
| 25.01069 | -80.64347 |  |  | GBIF |
| 25.01098 | -80.65818 |  |  | GBIF |
| 25.0764 | -80.67307 |  |  | GBIF |
| 24.99637 | -80.63103 |  |  | GBIF |
| 25.10283 | -80.79565 |  |  | GBIF |
| 25.00256 | -80.64456 |  |  | GBIF |
| 25.1014 | -80.7896 |  |  | GBIF |
| 25.10811 | -80.80687 |  |  | GBIF |
| 25.10663 | -80.79639 |  |  | GBIF |
| 25.10885 | -80.80524 |  |  | GBIF |
| 25.10644 | -80.7879 |  |  | GBIF |
| 25.11007 | -80.80707 |  |  | GBIF |
| 25.06373 | -80.74316 |  |  | GBIF |
| 25.05009 | -80.77331 |  |  | GBIF |
| 25.0712 | -80.77112 |  |  | GBIF |
| 25.07469 | -80.78684 |  |  | GBIF |
| 25.06925 | -80.74725 |  |  | GBIF |
| 25.0714 | -80.77888 |  |  | GBIF |
| 25.07333 | -80.73376 |  |  | GBIF |
| 25.08496 | -80.7721 |  |  | GBIF |
| 25.0821 | -80.78155 |  |  | GBIF |
| 25.08061 | -80.73405 |  |  | GBIF |
| 25.08545 | -80.76282 |  |  | GBIF |
| 25.08932 | -80.76626 |  |  | GBIF |
| 25.0892 | -80.78184 |  |  | GBIF |
| 25.09563 | -80.77786 |  |  | GBIF |
| 25.09314 | -80.75717 |  |  | GBIF |
| 25.09394 | -80.76917 |  |  | GBIF |
| 25.05172 | -80.76509 |  |  | GBIF |
| 25.09351 | -80.75925 |  |  | GBIF |
| 25.06758 | -80.7683 |  |  | GBIF |
| 25.04921 | -80.90755 |  |  | GBIF |
| 25.06384 | -80.782128 |  |  | GBIF |
| 25.05693 | -80.92799 |  |  | GBIF |
| 25.0535 | -80.91656 |  |  | GBIF |
| 25.04753 | -80.9228 |  |  | GBIF |
| 25.05833 | -80.77356 |  |  | GBIF |
| 25.03588 | -80.91676 |  |  | GBIF |
| 25.0576 | -80.90591 |  |  | GBIF |
| 25.06576 | -80.76127 |  |  | GBIF |
| 25.04756 | -80.90443 |  |  | GBIF |
| 25.06331 | -80.91923 |  |  | GBIF |
| 25.06304 | -80.91426 |  |  | GBIF |
| 25.06519 | -80.90167 |  |  | GBIF |
| 25.03538 | -80.92533 |  |  | GBIF |
| 25.03481 | -80.91245 |  |  | GBIF |
| 25.071611 | -80.906302 |  |  | GBIF |
| 25.06474 | -80.9344 |  |  | GBIF |
| 25.06853 | -80.91747 |  |  | GBIF |
| 25.07122 | -80.94037 |  |  | GBIF |
| 25.04369 | -80.91167 |  |  | GBIF |
| 25.04053 | -80.929 |  |  | GBIF |
| 24.98075 | -80.88212 |  |  | GBIF |
| 25.0424 | -80.90684 |  |  | GBIF |
| 25.0685 | -80.93247 |  |  | GBIF |
| 25.04432 | -80.9021 |  |  | GBIF |
| 25.03861 | -80.90571 |  |  | GBIF |
| 25.03999 | -80.92277 |  |  | GBIF |
| 24.97772 | -80.85018 |  |  | GBIF |
| 24.98497 | -80.86936 |  |  | GBIF |
| 24.99313 | -80.89745 |  |  | GBIF |
| 24.99573 | -80.87253 |  |  | GBIF |
| 24.97685 | -80.84259 |  |  | GBIF |
| 24.99702 | -80.88467 |  |  | GBIF |
| 25.00619 | -80.89622 |  |  | GBIF |
| 24.99738 | -80.86546 |  |  | GBIF |
| 25.0014 | -80.89271 |  |  | GBIF |
| 24.9863 | -80.85725 |  |  | GBIF |
| 25.00124 | -80.87032 |  |  | GBIF |
| 25.01588 | -80.88548 |  |  | GBIF |
| 24.98822 | -80.85054 |  |  | GBIF |
| 25.00799 | -80.89387 |  |  | GBIF |
| 25.01125 | -80.86638 |  |  | GBIF |
| 25.01402 | -80.85788 |  |  | GBIF |
| 25.01796 | -80.87983 |  |  | GBIF |
| 25.02078 | -80.89142 |  |  | GBIF |
| 24.97029 | -80.83611 |  |  | GBIF |
| 24.96695 | -80.84028 |  |  | GBIF |
| 25.08775 | -80.61308 |  |  | GBIF |
| 25.10048 | -80.58334 |  |  | GBIF |
| 25.08102 | -80.61026 |  |  | GBIF |
| 25.10351 | -80.60273 |  |  | GBIF |
| 25.07575 | -80.59387 |  |  | GBIF |
| 25.09267 | -80.62754 |  |  | GBIF |
| 25.10001 | -80.61846 |  |  | GBIF |
| 25.09202 | -80.5891 |  |  | GBIF |
| 25.05427 | -80.66992 |  |  | GBIF |
| 24.99532 | -80.6535 |  |  | GBIF |
| 25.05761 | -80.6685 |  |  | GBIF |
| 25.06718 | -80.68958 |  |  | GBIF |
| 25.06154 | -80.69069 |  |  | GBIF |
| 25.03006 | -80.64917 |  |  | GBIF |
| 24.99923 | -80.66245 |  |  | GBIF |
| 25.00479 | -80.64263 |  |  | GBIF |
| 25.05925 | -80.65504 |  |  | GBIF |
| 25.04814 | -80.65644 |  |  | GBIF |
| 25.10441 | -80.79597 |  |  | GBIF |
| 24.99809 | -80.64535 |  |  | GBIF |
| 25.06572 | -80.66389 |  |  | GBIF |
| 25.02508 | -80.64604 |  |  | GBIF |
| 24.99775 | -80.63874 |  |  | GBIF |
| 25.10699 | -80.80807 |  |  | GBIF |
| 25.10498 | -80.78867 |  |  | GBIF |
| 25.10687 | -80.80258 |  |  | GBIF |
| 25.105963 | -80.8002 |  |  | GBIF |
| 25.07195 | -80.73817 |  |  | GBIF |
| 25.10551 | -80.78822 |  |  | GBIF |
| 25.08709 | -80.75872 |  |  | GBIF |
| 25.08152 | -80.77937 |  |  | GBIF |
| 25.08268 | -80.74461 |  |  | GBIF |
| 25.07902 | -80.74973 |  |  | GBIF |
| 25.08661 | -80.78008 |  |  | GBIF |
| 25.09734 | -80.7699 |  |  | GBIF |
| 25.06927 | -80.74971 |  |  | GBIF |
| 25.06993 | -80.78751 |  |  | GBIF |
| 25.09888 | -80.78008 |  |  | GBIF |
| 25.08344 | -80.7653 |  |  | GBIF |
| 25.07002 | -80.7794 |  |  | GBIF |
| 25.0856 | -80.74194 |  |  | GBIF |
| 25.095 | -80.75962 |  |  | GBIF |
| 25.07903 | -80.76887 |  |  | GBIF |
| 25.07121 | -80.75971 |  |  | GBIF |
| 25.07286 | -80.77175 |  |  | GBIF |
| 25.0896 | -80.76164 |  |  | GBIF |
| 25.06589 | -80.77847 |  |  | GBIF |
| 25.0472 | -80.90409 |  |  | GBIF |
| 25.04902 | -80.91401 |  |  | GBIF |
| 25.06611 | -80.75491 |  |  | GBIF |
| 25.04907 | -80.92657 |  |  | GBIF |
| 25.0566 | -80.9313 |  |  | GBIF |
| 25.05025 | -80.91915 |  |  | GBIF |
| 25.05469 | -80.91099 |  |  | GBIF |
| 25.05774 | -80.7617 |  |  | GBIF |
| 25.03861 | -80.92883 |  |  | GBIF |
| 25.05609 | -80.92441 |  |  | GBIF |
| 25.05368 | -80.93587 |  |  | GBIF |
| 25.05117 | -80.93282 |  |  | GBIF |
| 25.03709 | -80.91859 |  |  | GBIF |
| 25.03631 | -80.9109 |  |  | GBIF |
| 25.05934 | -80.91112 |  |  | GBIF |
| 25.05793 | -80.91203 |  |  | GBIF |
| 25.06006 | -80.90631 |  |  | GBIF |
| 25.06476 | -80.91824 |  |  | GBIF |
| 24.97688 | -80.86602 |  |  | GBIF |
| 25.06277 | -80.93155 |  |  | GBIF |
| 25.03993 | -80.92111 |  |  | GBIF |
| 25.04442 | -80.9113 |  |  | GBIF |
| 25.06707 | -80.90834 |  |  | GBIF |
| 24.99069 | -80.86796 |  |  | GBIF |
| 24.98518 | -80.88812 |  |  | GBIF |
| 25.039 | -80.90173 |  |  | GBIF |
| 24.97937 | -80.87095 |  |  | GBIF |
| 24.98464 | -80.85259 |  |  | GBIF |
| 24.98794 | -80.8958 |  |  | GBIF |
| 24.98741 | -80.84598 |  |  | GBIF |
| 24.97691 | -80.84071 |  |  | GBIF |
| 24.99089 | -80.88103 |  |  | GBIF |
| 25.00589 | -80.90258 |  |  | GBIF |
| 24.98709 | -80.85509 |  |  | GBIF |
| 24.96234 | -80.83379 |  |  | GBIF |
| 25.01548 | -80.89358 |  |  | GBIF |
| 25.00774 | -80.86592 |  |  | GBIF |
| 25.00287 | -80.89561 |  |  | GBIF |
| 25.00507 | -80.87955 |  |  | GBIF |
| 24.99975 | -80.85349 |  |  | GBIF |
| 25.0059 | -80.86061 |  |  | GBIF |
| 25.0212 | -80.89473 |  |  | GBIF |
| 24.97416 | -80.89361 |  |  | GBIF |
| 25.00968 | -80.88862 |  |  | GBIF |
| 24.97695 | -80.8727 |  |  | GBIF |
| 24.98146 | -80.897 |  |  | GBIF |
| 24.97496 | -80.88973 |  |  | GBIF |
| 25.02197 | -80.87302 |  |  | GBIF |
| 24.97503 | -80.83167 |  |  | GBIF |
| 25.75824 | -80.15314 |  |  | GBIF |
| 25.75824 | -80.15468 |  |  | GBIF |
| 25.08341 | -80.59624 |  |  | GBIF |
| 25.09193 | -80.58791 |  |  | GBIF |
| 25.09597 | -80.61442 |  |  | GBIF |
| 25.11098 | -80.58533 |  |  | GBIF |
| 25.08883 | -80.61937 |  |  | GBIF |
| 25.10596 | -80.60168 |  |  | GBIF |
| 25.35024 | -80.31326 |  |  | GBIF |
| 25.30736 | -80.30333 |  |  | GBIF |
| 25.24519 | -80.34055 |  |  | GBIF |
| 25.36269 | -80.31061 |  |  | GBIF |
| 25.22363 | -80.35468 |  |  | GBIF |
| 25.30612 | -80.31186 |  |  | GBIF |
| 25.24609 | -80.36035 |  |  | GBIF |
| 25.3635 | -80.30292 |  |  | GBIF |
| 25.2107 | -80.36577 |  |  | GBIF |
| 25.26181 | -80.35796 |  |  | GBIF |
| 25.20685 | -80.37735 |  |  | GBIF |
| 25.19907 | -80.39418 |  |  | GBIF |
| 25.06289 | -80.672 |  |  | GBIF |
| 25.07976 | -80.69543 |  |  | GBIF |
| 25.07098 | -80.686 |  |  | GBIF |
| 25.08183 | -80.67096 |  |  | GBIF |
| 25.05303 | -80.65904 |  |  | GBIF |
| 25.06754 | -80.69331 |  |  | GBIF |
| 25.03904 | -80.66994 |  |  | GBIF |
| 25.06485 | -80.68154 |  |  | GBIF |
| 25.1037 | -80.79514 |  |  | GBIF |
| 25.10274 | -80.7874 |  |  | GBIF |
| 25.1068 | -80.79903 |  |  | GBIF |
| 25.10649 | -80.79448 |  |  | GBIF |
| 25.10657 | -80.8035 |  |  | GBIF |
| 25.10928 | -80.80726 |  |  | GBIF |
| 25.07852 | -80.78832 |  |  | GBIF |
| 25.07391 | -80.75737 |  |  | GBIF |
| 25.06075 | -80.7393 |  |  | GBIF |
| 25.07311 | -80.73367 |  |  | GBIF |
| 25.07653 | -80.76389 |  |  | GBIF |
| 25.07727 | -80.74629 |  |  | GBIF |
| 25.07429 | -80.77534 |  |  | GBIF |
| 25.07077 | -80.74799 |  |  | GBIF |
| 25.08635 | -80.76006 |  |  | GBIF |
| 25.08231 | -80.73257 |  |  | GBIF |
| 25.08186 | -80.77317 |  |  | GBIF |
| 25.08202 | -80.7776 |  |  | GBIF |
| 25.09144 | -80.75735 |  |  | GBIF |
| 25.0991 | -80.77806 |  |  | GBIF |
| 25.09337 | -80.76883 |  |  | GBIF |
| 25.08836 | -80.78084 |  |  | GBIF |
| 25.0594 | -80.74615 |  |  | GBIF |
| 25.06833 | -80.77056 |  |  | GBIF |
| 25.08751 | -80.7431 |  |  | GBIF |
| 25.06021 | -80.93665 |  |  | GBIF |
| 25.09601 | -80.77507 |  |  | GBIF |
| 25.05336 | -80.75284 |  |  | GBIF |
| 25.06633 | -80.78221 |  |  | GBIF |
| 25.06728 | -80.94289 |  |  | GBIF |
| 25.05293 | -80.93179 |  |  | GBIF |
| 25.04445 | -80.92346 |  |  | GBIF |
| 25.06677 | -80.91473 |  |  | GBIF |
| 25.06401 | -80.92159 |  |  | GBIF |
| 25.04002 | -80.90186 |  |  | GBIF |
| 25.05856 | -80.77556 |  |  | GBIF |
| 24.98501 | -80.87994 |  |  | GBIF |
| 25.05241 | -80.93475 |  |  | GBIF |
| 25.044 | -80.91512 |  |  | GBIF |
| 24.98791 | -80.89033 |  |  | GBIF |
| 25.03989 | -80.92007 |  |  | GBIF |
| 24.9836 | -80.8489 |  |  | GBIF |
| 24.97754 | -80.84241 |  |  | GBIF |
| 24.99769 | -80.89644 |  |  | GBIF |
| 25.05668 | -80.94223 |  |  | GBIF |
| 24.98013 | -80.869 |  |  | GBIF |
| 24.99691 | -80.88263 |  |  | GBIF |
| 24.99096 | -80.86924 |  |  | GBIF |
| 25.00092 | -80.88746 |  |  | GBIF |
| 25.04015 | -80.90598 |  |  | GBIF |
| 24.99611 | -80.8559 |  |  | GBIF |
| 25.01066 | -80.87682 |  |  | GBIF |
| 25.00218 | -80.86678 |  |  | GBIF |
| 24.99336 | -80.84504 |  |  | GBIF |
| 25.02352 | -80.89148 |  |  | GBIF |
| 25.00071 | -80.87389 |  |  | GBIF |
| 25.00777 | -80.90033 |  |  | GBIF |
| 25.0104 | -80.87444 |  |  | GBIF |
| 25.01558 | -80.85893 |  |  | GBIF |
| 25.01423 | -80.89523 |  |  | GBIF |
| 24.97515 | -80.86972 |  |  | GBIF |
| 24.97452 | -80.85386 |  |  | GBIF |
| 24.97472 | -80.88136 |  |  | GBIF |
| 25.01952 | -80.87485 |  |  | GBIF |
| 24.96814 | -80.83979 |  |  | GBIF |
| 25.75762 | -80.15299 |  |  | GBIF |
| 25.11605 | -80.57979 |  |  | GBIF |
| 25.10756 | -80.59463 |  |  | GBIF |
| 25.25333 | -80.34729 |  |  | GBIF |
| 25.31576 | -80.3012 |  |  | GBIF |
| 25.25832 | -80.35789 |  |  | GBIF |
| 25.31907 | -80.30565 |  |  | GBIF |
| 25.31699 | -80.30712 |  |  | GBIF |
| 25.37214 | -80.30428 |  |  | GBIF |
| 25.32171 | -80.32584 |  |  | GBIF |
| 25.22756 | -80.36343 |  |  | GBIF |
| 25.31115 | -80.32214 |  |  | GBIF |
| 25.24008 | -80.36009 |  |  | GBIF |
| 25.20026 | -80.37666 |  |  | GBIF |
| 25.20959 | -80.37601 |  |  | GBIF |
| 25.23802 | -80.36764 |  |  | GBIF |
| 25.19886 | -80.39246 |  |  | GBIF |
| 27.74 | -97.14 |  |  | GBIF |
| 25.01025 | -80.67317 |  |  | GBIF |
| 25.10524 | -80.79605 |  |  | GBIF |
| 25.1053 | -80.79319 |  |  | GBIF |
| 25.07482 | -80.67658 |  |  | GBIF |
| 25.10283 | -80.79196 |  |  | GBIF |
| 25.10598 | -80.81155 |  |  | GBIF |
| 25.10658 | -80.80237 |  |  | GBIF |
| 25.05029 | -80.77402 |  |  | GBIF |
| 25.07242 | -80.76921 |  |  | GBIF |
| 25.07401 | -80.75662 |  |  | GBIF |
| 25.06877 | -80.77581 |  |  | GBIF |
| 25.09479 | -80.76637 |  |  | GBIF |
| 25.08057 | -80.76016 |  |  | GBIF |
| 25.07876 | -80.78467 |  |  | GBIF |
| 25.08249 | -80.7742 |  |  | GBIF |
| 25.09271 | -80.75748 |  |  | GBIF |
| 25.07016 | -80.78304 |  |  | GBIF |
| 25.09271 | -80.77521 |  |  | GBIF |
| 25.07793 | -80.75512 |  |  | GBIF |
| 25.1025 | -80.77892 |  |  | GBIF |
| 25.07762 | -80.73531 |  |  | GBIF |
| 25.0559 | -80.76956 |  |  | GBIF |
| 25.06832 | -80.77979 |  |  | GBIF |
| 25.09332 | -80.77012 |  |  | GBIF |
| 25.06662 | -80.77186 |  |  | GBIF |
| 25.09821 | -80.76318 |  |  | GBIF |
| 25.05702 | -80.92263 |  |  | GBIF |
| 25.04917 | -80.90773 |  |  | GBIF |
| 25.04935 | -80.93464 |  |  | GBIF |
| 25.05632 | -80.91106 |  |  | GBIF |
| 25.04518 | -80.90387 |  |  | GBIF |
| 25.06181 | -80.90964 |  |  | GBIF |
| 25.06264 | -80.92105 |  |  | GBIF |
| 25.05581 | -80.90867 |  |  | GBIF |
| 25.06107 | -80.92749 |  |  | GBIF |
| 25.06556 | -80.90287 |  |  | GBIF |
| 25.06891 | -80.91431 |  |  | GBIF |
| 25.04178 | -80.89952 |  |  | GBIF |
| 24.98739 | -80.89039 |  |  | GBIF |
| 25.06626 | -80.90985 |  |  | GBIF |
| 24.98357 | -80.87492 |  |  | GBIF |
| 24.96513 | -80.84133 |  |  | GBIF |
| 25.06938 | -80.93256 |  |  | GBIF |
| 24.98474 | -80.84895 |  |  | GBIF |
| 24.99513 | -80.87144 |  |  | GBIF |
| 24.98299 | -80.86198 |  |  | GBIF |
| 24.99019 | -80.88708 |  |  | GBIF |
| 25.04406 | -80.9048 |  |  | GBIF |
| 24.9907 | -80.8526 |  |  | GBIF |
| 25.00439 | -80.88229 |  |  | GBIF |
| 24.96353 | -80.83221 |  |  | GBIF |
| 24.99643 | -80.84727 |  |  | GBIF |
| 24.97604 | -80.84007 |  |  | GBIF |
| 24.99357 | -80.8963 |  |  | GBIF |
| 25.01118 | -80.87623 |  |  | GBIF |
| 25.00685 | -80.90234 |  |  | GBIF |
| 24.99885 | -80.89271 |  |  | GBIF |
| 25.00057 | -80.86425 |  |  | GBIF |
| 25.02381 | -80.88783 |  |  | GBIF |
| 24.98665 | -80.89777 |  |  | GBIF |
| 25.0148 | -80.88878 |  |  | GBIF |
| 24.96952 | -80.8485 |  |  | GBIF |
| 25.0098 | -80.89252 |  |  | GBIF |
| 24.97723 | -80.88487 |  |  | GBIF |
| 24.97721 | -80.87198 |  |  | GBIF |
| 25.01754 | -80.87391 |  |  | GBIF |
| 24.96865 | -80.83674 |  |  | GBIF |
| 25.75827 | -80.15314 |  |  | GBIF |
| 25.75787 | -80.14934 |  |  | GBIF |
| 25.22146 | -80.36452 |  |  | GBIF |
| 25.31293 | -80.3095 |  |  | GBIF |
| 25.34428 | -80.30627 |  |  | GBIF |
| 25.30795 | -80.3014 |  |  | GBIF |
| 25.24813 | -80.34844 |  |  | GBIF |
| 25.25955 | -80.35843 |  |  | GBIF |
| 25.20328 | -80.37611 |  |  | GBIF |
| 25.23476 | -80.35106 |  |  | GBIF |
| 25.21114 | -80.37154 |  |  | GBIF |
| 25.20175 | -80.39083 |  |  | GBIF |
| 25.20873 | -80.37553 |  |  | GBIF |
| 25.16513 | -80.4598 |  |  | GBIF |
| 25.17177 | -80.4604 |  |  | GBIF |
| 25.11574 | -80.57903 |  |  | GBIF |
| 25.0831 | -80.62041 |  |  | GBIF |
| 25.09706 | -80.62555 |  |  | GBIF |
| 25.0865 | -80.60542 |  |  | GBIF |
| 25.10193 | -80.58562 |  |  | GBIF |
| 25.10328 | -80.59775 |  |  | GBIF |
| -20.18528 | -40.19127 |  |  | GBIF |
| 25.05918 | -80.66833 |  |  | GBIF |
| 25.0524 | -80.64854 |  |  | GBIF |
| 25.05491 | -80.67458 |  |  | GBIF |
| 25.04946 | -80.66705 |  |  | GBIF |
| 25.07199 | -80.67542 |  |  | GBIF |
| 25.10247 | -80.79603 |  |  | GBIF |
| 25.00998 | -80.65412 |  |  | GBIF |
| 25.05898 | -80.65868 |  |  | GBIF |
| 25.07258 | -80.66453 |  |  | GBIF |
| 25.07911 | -80.69014 |  |  | GBIF |
| 24.99165 | -80.65163 |  |  | GBIF |
| 25.06312 | -80.6777 |  |  | GBIF |
| 25.00691 | -80.64079 |  |  | GBIF |
| 25.00664 | -80.62847 |  |  | GBIF |
| 25.04723 | -80.69064 |  |  | GBIF |
| 25.06567 | -80.6911 |  |  | GBIF |
| 25.00858 | -80.6389 |  |  | GBIF |
| 25.10278 | -80.78729 |  |  | GBIF |
| 25.11069 | -80.80759 |  |  | GBIF |
| 25.10856 | -80.80308 |  |  | GBIF |
| 25.10816 | -80.79794 |  |  | GBIF |
| 25.07459 | -80.77638 |  |  | GBIF |
| 25.11024 | -80.8086 |  |  | GBIF |
| 25.06677 | -80.75065 |  |  | GBIF |
| 25.07518 | -80.78298 |  |  | GBIF |
| 25.07789 | -80.75521 |  |  | GBIF |
| 25.08197 | -80.77577 |  |  | GBIF |
| 25.07903 | -80.76431 |  |  | GBIF |
| 25.08225 | -80.78025 |  |  | GBIF |
| 25.07951 | -80.7264 |  |  | GBIF |
| 25.08793 | -80.75418 |  |  | GBIF |
| 25.08605 | -80.77432 |  |  | GBIF |
| 25.07175 | -80.74656 |  |  | GBIF |
| 25.09715 | -80.77711 |  |  | GBIF |
| 25.09895 | -80.7604 |  |  | GBIF |
| 25.08579 | -80.74312 |  |  | GBIF |
| 25.0998 | -80.77409 |  |  | GBIF |
| 25.08455 | -80.76659 |  |  | GBIF |
| 25.06835 | -80.78712 |  |  | GBIF |
| 25.08615 | -80.74996 |  |  | GBIF |
| 25.05063 | -80.92629 |  |  | GBIF |
| 25.05365 | -80.74597 |  |  | GBIF |
| 25.04838 | -80.90607 |  |  | GBIF |
| 25.0373 | -80.92986 |  |  | GBIF |
| 25.04894 | -80.9223 |  |  | GBIF |
| 25.05123 | -80.9099 |  |  | GBIF |
| 25.05656 | -80.91861 |  |  | GBIF |
| 25.06255 | -80.94106 |  |  | GBIF |
| 25.05426 | -80.92133 |  |  | GBIF |
| 25.03487 | -80.91971 |  |  | GBIF |
| 25.05956 | -80.91386 |  |  | GBIF |
| 25.03356 | -80.90893 |  |  | GBIF |
| 25.06102 | -80.93517 |  |  | GBIF |
| 25.05607 | -80.91087 |  |  | GBIF |
| 25.06967 | -80.90928 |  |  | GBIF |
| 25.06231 | -80.90596 |  |  | GBIF |
| 25.06307 | -80.9217 |  |  | GBIF |
| 24.98374 | -80.86817 |  |  | GBIF |
| 24.98256 | -80.88234 |  |  | GBIF |
| 25.06703 | -80.94135 |  |  | GBIF |
| 25.06769 | -80.93185 |  |  | GBIF |
| 25.0341 | -80.90528 |  |  | GBIF |
| 25.07086 | -80.91782 |  |  | GBIF |
| 25.0436 | -80.90147 |  |  | GBIF |
| 25.04303 | -80.93134 |  |  | GBIF |
| 24.99301 | -80.86217 |  |  | GBIF |
| 24.96199 | -80.8409 |  |  | GBIF |
| 24.98314 | -80.84742 |  |  | GBIF |
| 24.98013 | -80.89085 |  |  | GBIF |
| 25.04104 | -80.90416 |  |  | GBIF |
| 25.03907 | -80.92483 |  |  | GBIF |
| 25.04533 | -80.91467 |  |  | GBIF |
| 25.00397 | -80.89066 |  |  | GBIF |
| 25.0062 | -80.86865 |  |  | GBIF |
| 25.01354 | -80.88919 |  |  | GBIF |
| 24.98727 | -80.84296 |  |  | GBIF |
| 24.98287 | -80.84167 |  |  | GBIF |
| 24.99425 | -80.8642 |  |  | GBIF |
| 24.97817 | -80.89475 |  |  | GBIF |
| 25.01075 | -80.88142 |  |  | GBIF |
| 25.00708 | -80.85192 |  |  | GBIF |
| 24.97434 | -80.83294 |  |  | GBIF |
| 25.02308 | -80.87414 |  |  | GBIF |
| 24.97495 | -80.88016 |  |  | GBIF |
| 24.97005 | -80.84241 |  |  | GBIF |
| 25.761983 | -80.150916 |  |  | GBIF |
| 25.757833 | -80.151383 |  |  | GBIF |
| 25.367 | -80.30634 |  |  | GBIF |
| 25.31812 | -80.30572 |  |  | GBIF |
| 25.31101 | -80.31487 |  |  | GBIF |
| 25.21981 | -80.37416 |  |  | GBIF |
| 25.30998 | -80.30323 |  |  | GBIF |
| 25.35076 | -80.26939 |  |  | GBIF |
| 25.23849 | -80.3587 |  |  | GBIF |
| 25.36453 | -80.31424 |  |  | GBIF |
| 25.32367 | -80.29419 |  |  | GBIF |
| 25.24746 | -80.36397 |  |  | GBIF |
| 25.36945 | -80.27307 |  |  | GBIF |
| 25.25424 | -80.34977 |  |  | GBIF |
| 25.25874 | -80.35024 |  |  | GBIF |
| 25.19808 | -80.37579 |  |  | GBIF |
| 25.20938 | -80.37761 |  |  | GBIF |
| 25.20983 | -80.37217 |  |  | GBIF |
| 25.09672 | -80.59734 |  |  | GBIF |
| 25.10223 | -80.60692 |  |  | GBIF |
| 25.10258 | -80.5771 |  |  | GBIF |
| 25.06303 | -80.6778 |  |  | GBIF |
| 25.07185 | -80.67577 |  |  | GBIF |
| 25.04948 | -80.66708 |  |  | GBIF |
| 25.04807 | -80.64672 |  |  | GBIF |
| 25.03914 | -80.68938 |  |  | GBIF |
| 25.05555 | -80.64833 |  |  | GBIF |
| 25.01746 | -80.64837 |  |  | GBIF |
| 25.04733 | -80.6907 |  |  | GBIF |
| 25.07914 | -80.69016 |  |  | GBIF |
| 25.05488 | -80.67485 |  |  | GBIF |
| 25.07254 | -80.66485 |  |  | GBIF |
| 25.00453 | -80.64864 |  |  | GBIF |
| 25.07801 | -80.66798 |  |  | GBIF |
| 25.10501 | -80.79501 |  |  | GBIF |
| 25.10361 | -80.7927 |  |  | GBIF |
| 25.04379 | -80.6563 |  |  | GBIF |
| 25.01656 | -80.64384 |  |  | GBIF |
| 25.05898 | -80.65907 |  |  | GBIF |
| 25.10907 | -80.80534 |  |  | GBIF |
| 25.1072 | -80.80959 |  |  | GBIF |
| 25.10793 | -80.79694 |  |  | GBIF |
| 25.06781 | -80.74401 |  |  | GBIF |
| 25.07598 | -80.78419 |  |  | GBIF |
| 25.07563 | -80.7765 |  |  | GBIF |
| 25.07867 | -80.776 |  |  | GBIF |
| 25.05164 | -80.77254 |  |  | GBIF |
| 25.06262 | -80.74803 |  |  | GBIF |
| 25.08012 | -80.78475 |  |  | GBIF |
| 25.09412 | -80.76694 |  |  | GBIF |
| 25.07344 | -80.7698 |  |  | GBIF |
| 25.09671 | -80.77869 |  |  | GBIF |
| 25.09565 | -80.76079 |  |  | GBIF |
| 25.08344 | -80.76215 |  |  | GBIF |
| 25.10227 | -80.774 |  |  | GBIF |
| 25.07597 | -80.75452 |  |  | GBIF |
| 25.0881 | -80.75153 |  |  | GBIF |
| 25.08822 | -80.78182 |  |  | GBIF |
| 25.06198 | -80.91827 |  |  | GBIF |
| 25.05809 | -80.91656 |  |  | GBIF |
| 25.05063 | -80.76434 |  |  | GBIF |
| 25.0734 | -80.73595 |  |  | GBIF |
| 25.07759 | -80.75346 |  |  | GBIF |
| 25.05743 | -80.77437 |  |  | GBIF |
| 25.05532 | -80.76105 |  |  | GBIF |
| 25.05718 | -80.90733 |  |  | GBIF |
| 25.08453 | -80.73948 |  |  | GBIF |
| 25.064 | -80.91049 |  |  | GBIF |
| 25.03697 | -80.92761 |  |  | GBIF |
| 25.04574 | -80.90233 |  |  | GBIF |
| 25.05618 | -80.93861 |  |  | GBIF |
| 25.05501 | -80.74941 |  |  | GBIF |
| 25.06059 | -80.78017 |  |  | GBIF |
| 25.0546 | -80.91948 |  |  | GBIF |
| 25.06052 | -80.90358 |  |  | GBIF |
| 25.06922 | -80.94053 |  |  | GBIF |
| 25.06937 | -80.92456 |  |  | GBIF |
| 25.06362 | -80.77054 |  |  | GBIF |
| 25.03822 | -80.9097 |  |  | GBIF |
| 25.04451 | -80.90626 |  |  | GBIF |
| 25.07015 | -80.90408 |  |  | GBIF |
| 25.07046 | -80.91709 |  |  | GBIF |
| 25.0381 | -80.90449 |  |  | GBIF |
| 25.0403 | -80.91115 |  |  | GBIF |
| 24.99224 | -80.88386 |  |  | GBIF |
| 24.98551 | -80.87089 |  |  | GBIF |
| 24.98208 | -80.87009 |  |  | GBIF |
| 24.98427 | -80.89005 |  |  | GBIF |
| 25.04004 | -80.90112 |  |  | GBIF |
| 24.98983 | -80.86423 |  |  | GBIF |
| 24.96512 | -80.83441 |  |  | GBIF |
| 24.9799 | -80.8504 |  |  | GBIF |
| 24.97636 | -80.83565 |  |  | GBIF |
| 24.99448 | -80.88947 |  |  | GBIF |
| 25.06461 | -80.94318 |  |  | GBIF |
| 24.98669 | -80.85989 |  |  | GBIF |
| 25.00303 | -80.90221 |  |  | GBIF |
| 25.00031 | -80.88224 |  |  | GBIF |
| 25.00776 | -80.89027 |  |  | GBIF |
| 25.00395 | -80.85619 |  |  | GBIF |
| 25.0018 | -80.86915 |  |  | GBIF |
| 24.98738 | -80.84312 |  |  | GBIF |
| 25.01599 | -80.89502 |  |  | GBIF |
| 25.00751 | -80.88091 |  |  | GBIF |
| 25.01069 | -80.87476 |  |  | GBIF |
| 25.02576 | -80.89037 |  |  | GBIF |
| 24.97277 | -80.8572 |  |  | GBIF |
| 24.97396 | -80.89198 |  |  | GBIF |
| 24.97709 | -80.88123 |  |  | GBIF |
| 25.01809 | -80.87741 |  |  | GBIF |
| 25.01795 | -80.86534 |  |  | GBIF |
| 24.97422 | -80.84214 |  |  | GBIF |
| 24.98134 | -80.89639 |  |  | GBIF |
| 24.969 | -80.83416 |  |  | GBIF |
| 25.75976 | -80.1543 |  |  | GBIF |
| 25.3555 | -80.3044 |  |  | GBIF |
| 25.36699 | -80.30603 |  |  | GBIF |
| 25.35069 | -80.26933 |  |  | GBIF |
| 25.32381 | -80.29404 |  |  | GBIF |
| 25.30984 | -80.30316 |  |  | GBIF |
| 25.31811 | -80.30559 |  |  | GBIF |
| 25.22389 | -80.35996 |  |  | GBIF |
| 25.23946 | -80.36291 |  |  | GBIF |
| 25.36454 | -80.31424 |  |  | GBIF |
| 25.23185 | -80.35413 |  |  | GBIF |
| 25.24863 | -80.35989 |  |  | GBIF |
| 25.2475 | -80.3452 |  |  | GBIF |
| 25.25883 | -80.35039 |  |  | GBIF |
| 25.19947 | -80.37453 |  |  | GBIF |
| 25.16963 | -80.45647 |  |  | GBIF |
| 25.11786 | -80.58939 |  |  | GBIF |
| 25.1619 | -80.46127 |  |  | GBIF |
| 25.11382 | -80.58134 |  |  | GBIF |
| 25.07669 | -80.68659 |  |  | GBIF |
| 25.00502 | -80.65025 |  |  | GBIF |
| 25.103 | -80.79801 |  |  | GBIF |
| 25.06397 | -80.68228 |  |  | GBIF |
| 25.06909 | -80.67695 |  |  | GBIF |
| 25.00491 | -80.64423 |  |  | GBIF |
| 25.01607 | -80.64949 |  |  | GBIF |
| 25.00028 | -80.6448 |  |  | GBIF |
| 25.07753 | -80.68296 |  |  | GBIF |
| 25.00921 | -80.65274 |  |  | GBIF |
| 25.10892 | -80.80711 |  |  | GBIF |
| 25.1093 | -80.80704 |  |  | GBIF |
| 25.08941 | -80.7607 |  |  | GBIF |
| 25.07123 | -80.75739 |  |  | GBIF |
| 25.07633 | -80.78812 |  |  | GBIF |
| 25.07284 | -80.77779 |  |  | GBIF |
| 25.07572 | -80.73437 |  |  | GBIF |
| 25.0835 | -80.7837 |  |  | GBIF |
| 25.08219 | -80.74577 |  |  | GBIF |
| 25.06109 | -80.76614 |  |  | GBIF |
| 25.07284 | -80.74861 |  |  | GBIF |
| 25.07244 | -80.76688 |  |  | GBIF |
| 25.08593 | -80.7751 |  |  | GBIF |
| 25.08587 | -80.77487 |  |  | GBIF |
| 25.04508 | -80.75885 |  |  | GBIF |
| 25.07951 | -80.77534 |  |  | GBIF |
| 25.08602 | -80.76435 |  |  | GBIF |
| 25.08286 | -80.75777 |  |  | GBIF |
| 25.09572 | -80.78033 |  |  | GBIF |
| 25.04752 | -80.9253 |  |  | GBIF |
| 25.08547 | -80.74375 |  |  | GBIF |
| 25.05602 | -80.7634 |  |  | GBIF |
| 25.04706 | -80.91751 |  |  | GBIF |
| 25.08458 | -80.73803 |  |  | GBIF |
| 25.06502 | -80.90068 |  |  | GBIF |
| 25.04975 | -80.90154 |  |  | GBIF |
| 25.06246 | -80.7629 |  |  | GBIF |
| 25.05691 | -80.90796 |  |  | GBIF |
| 25.05599 | -80.74937 |  |  | GBIF |
| 25.06156 | -80.7684 |  |  | GBIF |
| 25.06824 | -80.78013 |  |  | GBIF |
| 25.09087 | -80.76711 |  |  | GBIF |
| 25.03644 | -80.92991 |  |  | GBIF |
| 25.05469 | -80.78291 |  |  | GBIF |
| 25.05394 | -80.9141 |  |  | GBIF |
| 25.05044 | -80.90928 |  |  | GBIF |
| 25.04492 | -80.92139 |  |  | GBIF |
| 25.05647 | -80.9281 |  |  | GBIF |
| 25.05719 | -80.92426 |  |  | GBIF |
| 25.06583 | -80.92168 |  |  | GBIF |
| 25.03916 | -80.90788 |  |  | GBIF |
| 25.06828 | -80.90977 |  |  | GBIF |
| 24.98154 | -80.86239 |  |  | GBIF |
| 25.038 | -80.89891 |  |  | GBIF |
| 25.06223 | -80.91194 |  |  | GBIF |
| 25.06484 | -80.91837 |  |  | GBIF |
| 25.0598 | -80.9463 |  |  | GBIF |
| 25.03971 | -80.91392 |  |  | GBIF |
| 25.03636 | -80.92179 |  |  | GBIF |
| 25.03689 | -80.90879 |  |  | GBIF |
| 24.99023 | -80.84945 |  |  | GBIF |
| 25.03547 | -80.8988 |  |  | GBIF |
| 24.98239 | -80.87357 |  |  | GBIF |
| 24.97832 | -80.89109 |  |  | GBIF |
| 24.99673 | -80.88454 |  |  | GBIF |
| 25.0449 | -80.932 |  |  | GBIF |
| 24.98174 | -80.84377 |  |  | GBIF |
| 24.97942 | -80.85487 |  |  | GBIF |
| 24.995 | -80.89092 |  |  | GBIF |
| 24.98771 | -80.87108 |  |  | GBIF |
| 24.99916 | -80.88712 |  |  | GBIF |
| 25.00281 | -80.87614 |  |  | GBIF |
| 25.01945 | -80.87759 |  |  | GBIF |
| 24.99746 | -80.86475 |  |  | GBIF |
| 24.96477 | -80.82616 |  |  | GBIF |
| 25.00877 | -80.89719 |  |  | GBIF |
| 25.01047 | -80.87923 |  |  | GBIF |
| 25.01712 | -80.8727 |  |  | GBIF |
| 25.01947 | -80.8595 |  |  | GBIF |
| 24.9865 | -80.85896 |  |  | GBIF |
| 25.0007 | -80.85172 |  |  | GBIF |
| 24.9776 | -80.85762 |  |  | GBIF |
| 24.97095 | -80.84058 |  |  | GBIF |
| 24.97195 | -80.87361 |  |  | GBIF |
| 24.97749 | -80.88223 |  |  | GBIF |
| 24.97383 | -80.89814 |  |  | GBIF |
| 24.97015 | -80.83578 |  |  | GBIF |
| 24.98572 | -80.90758 |  |  | GBIF |
| 25.75846 | -80.15237 |  |  | GBIF |
| 25.22448 | -80.36616 |  |  | GBIF |
| 25.32173 | -80.32802 |  |  | GBIF |
| 25.23551 | -80.34882 |  |  | GBIF |
| 25.24538 | -80.3569 |  |  | GBIF |
| 25.24373 | -80.34757 |  |  | GBIF |
| 25.25778 | -80.34731 |  |  | GBIF |
| 25.21691 | -80.36495 |  |  | GBIF |
| 25.16724 | -80.46233 |  |  | GBIF |
| 25.09908 | -80.58584 |  |  | GBIF |
| 25.08676 | -80.63159 |  |  | GBIF |
| 25.0964 | -80.60878 |  |  | GBIF |
| 25.10528 | -80.60529 |  |  | GBIF |
| 25.09982 | -80.58429 |  |  | GBIF |
| 12.173333 | -83.035278 |  |  | GBIF |
| 25.0607 | -80.68513 |  |  | GBIF |
| 25.10503 | -80.79434 |  |  | GBIF |
| 25.04768 | -80.66319 |  |  | GBIF |
| 24.99449 | -80.63499 |  |  | GBIF |
| 25.0432 | -80.65635 |  |  | GBIF |
| 25.07432 | -80.68083 |  |  | GBIF |
| 25.06754 | -80.67901 |  |  | GBIF |
| 25.00288 | -80.63901 |  |  | GBIF |
| 25.10167 | -80.79163 |  |  | GBIF |
| 25.10656 | -80.79134 |  |  | GBIF |
| 25.10741 | -80.81136 |  |  | GBIF |
| 25.10779 | -80.79846 |  |  | GBIF |
| 25.10937 | -80.80238 |  |  | GBIF |
| 25.07077 | -80.76272 |  |  | GBIF |
| 25.07505 | -80.78876 |  |  | GBIF |
| 25.07643 | -80.77659 |  |  | GBIF |
| 25.07219 | -80.74457 |  |  | GBIF |
| 25.07269 | -80.75552 |  |  | GBIF |
| 25.06423 | -80.74621 |  |  | GBIF |
| 25.07918 | -80.771 |  |  | GBIF |
| 25.07199 | -80.73497 |  |  | GBIF |
| 25.08364 | -80.78294 |  |  | GBIF |
| 25.09358 | -80.77955 |  |  | GBIF |
| 25.0805 | -80.76324 |  |  | GBIF |
| 25.09168 | -80.75429 |  |  | GBIF |
| 25.04947 | -80.75639 |  |  | GBIF |
| 25.0858 | -80.76809 |  |  | GBIF |
| 25.09978 | -80.7769 |  |  | GBIF |
| 25.0973 | -80.76643 |  |  | GBIF |
| 25.0976 | -80.76511 |  |  | GBIF |
| 25.03796 | -80.93029 |  |  | GBIF |
| 25.06021 | -80.78403 |  |  | GBIF |
| 25.0525 | -80.76704 |  |  | GBIF |
| 25.05503 | -80.77237 |  |  | GBIF |
| 25.06365 | -80.94698 |  |  | GBIF |
| 25.0655 | -80.77589 |  |  | GBIF |
| 25.04967 | -80.92999 |  |  | GBIF |
| 25.05632 | -80.74783 |  |  | GBIF |
| 25.05381 | -80.90755 |  |  | GBIF |
| 25.05676 | -80.9222 |  |  | GBIF |
| 25.04511 | -80.91819 |  |  | GBIF |
| 25.04579 | -80.90274 |  |  | GBIF |
| 25.0465 | -80.91235 |  |  | GBIF |
| 25.06011 | -80.91955 |  |  | GBIF |
| 25.06434 | -80.90106 |  |  | GBIF |
| 25.06366 | -80.90994 |  |  | GBIF |
| 25.05867 | -80.93839 |  |  | GBIF |
| 25.05742 | -80.91369 |  |  | GBIF |
| 25.0585 | -80.93049 |  |  | GBIF |
| 25.07018 | -80.90698 |  |  | GBIF |
| 25.06852 | -80.9408 |  |  | GBIF |
| 25.06803 | -80.91244 |  |  | GBIF |
| 25.0444 | -80.91419 |  |  | GBIF |
| 25.06126 | -80.92802 |  |  | GBIF |
| 25.03584 | -80.90939 |  |  | GBIF |
| 25.0386 | -80.90559 |  |  | GBIF |
| 25.0656 | -80.92264 |  |  | GBIF |
| 25.04267 | -80.93404 |  |  | GBIF |
| 24.97687 | -80.87997 |  |  | GBIF |
| 25.04437 | -80.92603 |  |  | GBIF |
| 24.98095 | -80.85997 |  |  | GBIF |
| 25.04329 | -80.90907 |  |  | GBIF |
| 24.9775 | -80.83865 |  |  | GBIF |
| 24.99538 | -80.88371 |  |  | GBIF |
| 24.98621 | -80.85043 |  |  | GBIF |
| 25.0401 | -80.89998 |  |  | GBIF |
| 24.98932 | -80.90056 |  |  | GBIF |
| 24.98418 | -80.88873 |  |  | GBIF |
| 24.96446 | -80.82295 |  |  | GBIF |
| 25.00328 | -80.89159 |  |  | GBIF |
| 24.99585 | -80.86702 |  |  | GBIF |
| 24.99137 | -80.85683 |  |  | GBIF |
| 24.9997 | -80.90676 |  |  | GBIF |
| 25.01531 | -80.89101 |  |  | GBIF |
| 24.99912 | -80.87429 |  |  | GBIF |
| 25.00539 | -80.86545 |  |  | GBIF |
| 25.01509 | -80.88625 |  |  | GBIF |
| 24.98544 | -80.90482 |  |  | GBIF |
| 24.99377 | -80.8387 |  |  | GBIF |
| 25.02359 | -80.89304 |  |  | GBIF |
| 25.01816 | -80.87982 |  |  | GBIF |
| 25.02362 | -80.8604 |  |  | GBIF |
| 24.97597 | -80.90272 |  |  | GBIF |
| 24.9724 | -80.84465 |  |  | GBIF |
| 25.75401 | -80.15397 |  |  | GBIF |
| 25.76174 | -80.15009 |  |  | GBIF |
| 25.76328 | -80.15325 |  |  | GBIF |
| 25.30681 | -80.30798 |  |  | GBIF |
| 25.31719 | -80.29246 |  |  | GBIF |
| 25.31236 | -80.32189 |  |  | GBIF |
| 25.32809 | -80.32249 |  |  | GBIF |
| 25.34607 | -80.27454 |  |  | GBIF |
| 25.3239 | -80.29102 |  |  | GBIF |
| 25.23104 | -80.36673 |  |  | GBIF |
| 25.3117 | -80.3149 |  |  | GBIF |
| 25.22527 | -80.37189 |  |  | GBIF |
| 25.2207 | -80.35802 |  |  | GBIF |
| 25.24274 | -80.34547 |  |  | GBIF |
| 25.23495 | -80.35339 |  |  | GBIF |
| 25.25339 | -80.35737 |  |  | GBIF |
| 25.199 | -80.39651 |  |  | GBIF |
| 25.263 | -80.35403 |  |  | GBIF |
| 25.2071 | -80.37521 |  |  | GBIF |
| 25.21817 | -80.36659 |  |  | GBIF |
| 25.11453 | -80.58894 |  |  | GBIF |
| 25.15773 | -80.49123 |  |  | GBIF |
| 25.16934 | -80.46178 |  |  | GBIF |
| 25.10606 | -80.58699 |  |  | GBIF |
| 25.10932 | -80.59222 |  |  | GBIF |
| 25.09919 | -80.61141 |  |  | GBIF |
| 24.562833 | -82.107167 |  |  | GBIF |
| 24.658167 | -81.4205 |  |  | GBIF |
| 25.02257 | -80.65397 |  |  | GBIF |
| 25.08027 | -80.68795 |  |  | GBIF |
| 25.07469 | -80.67517 |  |  | GBIF |
| 25.07422 | -80.66281 |  |  | GBIF |
| 24.99699 | -80.65736 |  |  | GBIF |
| 25.102867 | -80.793017 |  |  | GBIF |
| 24.9946 | -80.6535 |  |  | GBIF |
| 25.07543 | -80.68676 |  |  | GBIF |
| 25.00729 | -80.6358 |  |  | GBIF |
| 25.101617 | -80.790567 |  |  | GBIF |
| 25.109667 | -80.802833 |  |  | GBIF |
| 25.111633 | -80.8085 |  |  | GBIF |
| 25.108183 | -80.807967 |  |  | GBIF |
| 25.107783 | -80.79785 |  |  | GBIF |
| 25.0805 | -80.78458 |  |  | GBIF |
| 25.10635 | -80.792433 |  |  | GBIF |
| 25.06623 | -80.75348 |  |  | GBIF |
| 25.0633 | -80.74331 |  |  | GBIF |
| 25.08199 | -80.76369 |  |  | GBIF |
| 25.07915 | -80.77069 |  |  | GBIF |
| 25.07733 | -80.78763 |  |  | GBIF |
| 25.09318 | -80.77597 |  |  | GBIF |
| 25.08033 | -80.73182 |  |  | GBIF |
| 25.07555 | -80.77592 |  |  | GBIF |
| 25.05747 | -80.77587 |  |  | GBIF |
| 25.08343 | -80.74998 |  |  | GBIF |
| 25.08017 | -80.74349 |  |  | GBIF |
| 25.0922 | -80.757717 |  |  | GBIF |
| 25.092167 | -80.76105 |  |  | GBIF |
| 25.099333 | -80.77735 |  |  | GBIF |
| 25.09767 | -80.77007 |  |  | GBIF |
| 25.09075 | -80.74735 |  |  | GBIF |
| 25.092717 | -80.760067 |  |  | GBIF |
| 25.05537 | -80.74963 |  |  | GBIF |
| 25.06713 | -80.75694 |  |  | GBIF |
| 25.05092 | -80.90551 |  |  | GBIF |
| 25.06183 | -80.93764 |  |  | GBIF |
| 25.049 | -80.92712 |  |  | GBIF |
| 25.06442 | -80.94342 |  |  | GBIF |
| 25.06804 | -80.7867 |  |  | GBIF |
| 25.06401 | -80.90749 |  |  | GBIF |
| 25.05495 | -80.94003 |  |  | GBIF |
| 25.05653 | -80.90587 |  |  | GBIF |
| 25.05408 | -80.93453 |  |  | GBIF |
| 25.0599 | -80.90347 |  |  | GBIF |
| 25.06513 | -80.92064 |  |  | GBIF |
| 25.07031 | -80.93907 |  |  | GBIF |
| 25.07134 | -80.93221 |  |  | GBIF |
| 25.06171 | -80.92729 |  |  | GBIF |
| 25.06948 | -80.92207 |  |  | GBIF |
| 25.06865 | -80.91374 |  |  | GBIF |
| 25.06751 | -80.90376 |  |  | GBIF |
| 25.04091 | -80.90095 |  |  | GBIF |
| 25.04413 | -80.90702 |  |  | GBIF |
| 25.76057 | -80.14933 |  |  | GBIF |
| 25.75613 | -80.1522 |  |  | GBIF |
| 25.22211 | -80.36794 |  |  | GBIF |
| 25.3146 | -80.31017 |  |  | GBIF |
| 25.2291 | -80.35406 |  |  | GBIF |
| 25.32548 | -80.30693 |  |  | GBIF |
| 25.22885 | -80.361933 |  |  | GBIF |
| 25.2542 | -80.34793 |  |  | GBIF |
| 25.25624 | -80.35462 |  |  | GBIF |
| 25.37628 | -80.30711 |  |  | GBIF |
| 25.24634 | -80.3588 |  |  | GBIF |
| 25.20384 | -80.38436 |  |  | GBIF |
| 25.21579 | -80.36753 |  |  | GBIF |
| 25.11575 | -80.58958 |  |  | GBIF |
| 25.0812 | -80.60239 |  |  | GBIF |
| 25.15807 | -80.4632 |  |  | GBIF |
| 25.09696 | -80.59983 |  |  | GBIF |
| 25.0811 | -80.61482 |  |  | GBIF |
| 25.12451 | -80.57254 |  |  | GBIF |
| 25.09256 | -80.59142 |  |  | GBIF |
| 24.98095 | -80.88324 |  |  | GBIF |
| 24.98132 | -80.86565 |  |  | GBIF |
| 24.99273 | -80.89349 |  |  | GBIF |
| 24.99502 | -80.88602 |  |  | GBIF |
| 24.98887 | -80.84653 |  |  | GBIF |
| 24.98549 | -80.89581 |  |  | GBIF |
| 24.99265 | -80.86552 |  |  | GBIF |
| 24.98101 | -80.83807 |  |  | GBIF |
| 24.9978 | -80.85616 |  |  | GBIF |
| 25.00676 | -80.90269 |  |  | GBIF |
| 25.0087 | -80.86206 |  |  | GBIF |
| 25.00328 | -80.88077 |  |  | GBIF |
| 25.00343 | -80.89278 |  |  | GBIF |
| 25.00481 | -80.85461 |  |  | GBIF |
| 25.0161 | -80.89299 |  |  | GBIF |
| 25.02416 | -80.88923 |  |  | GBIF |
| 25.01036 | -80.877 |  |  | GBIF |
| 25.01486 | -80.87557 |  |  | GBIF |
| 25.01962 | -80.86658 |  |  | GBIF |
| 24.97309 | -80.88871 |  |  | GBIF |
| 24.97434 | -80.84394 |  |  | GBIF |
| 25.01906 | -80.88171 |  |  | GBIF |
| 24.97979 | -80.8979 |  |  | GBIF |
| 24.97373 | -80.85638 |  |  | GBIF |
| 24.97204 | -80.87033 |  |  | GBIF |
| 25.10369 | -80.79462 |  |  | GBIF |
| 25.10533 | -80.7897 |  |  | GBIF |
| 25.10879 | -80.80855 |  |  | GBIF |
| 25.10903 | -80.80541 |  |  | GBIF |
| 25.0867 | -80.76884 |  |  | GBIF |
| 25.10734 | -80.79306 |  |  | GBIF |
| 25.06902 | -80.76781 |  |  | GBIF |
| 25.08283 | -80.77337 |  |  | GBIF |
| 25.08002 | -80.78527 |  |  | GBIF |
| 25.07458 | -80.77452 |  |  | GBIF |
| 25.08969 | -80.75491 |  |  | GBIF |
| 25.08489 | -80.74256 |  |  | GBIF |
| 25.0836 | -80.76056 |  |  | GBIF |
| 25.06792 | -80.74785 |  |  | GBIF |
| 25.07878 | -80.73365 |  |  | GBIF |
| 25.0813 | -80.75373 |  |  | GBIF |
| 25.09723 | -80.7794 |  |  | GBIF |
| 25.07486 | -80.78458 |  |  | GBIF |
| 25.0948 | -80.7748 |  |  | GBIF |
| 25.09151 | -80.77394 |  |  | GBIF |
| 25.04686 | -80.91856 |  |  | GBIF |
| 25.07316 | -80.75768 |  |  | GBIF |
| 25.0505 | -80.93394 |  |  | GBIF |
| 25.04994 | -80.92686 |  |  | GBIF |
| 25.05476 | -80.93891 |  |  | GBIF |
| 25.03816 | -80.91883 |  |  | GBIF |
| 25.0353 | -80.92748 |  |  | GBIF |
| 25.05021 | -80.9073 |  |  | GBIF |
| 25.06869 | -80.9144 |  |  | GBIF |
| 25.0622 | -80.78204 |  |  | GBIF |
| 25.0556 | -80.934 |  |  | GBIF |
| 25.04933 | -80.90352 |  |  | GBIF |
| 25.06408 | -80.92592 |  |  | GBIF |
| 25.03469 | -80.91204 |  |  | GBIF |
| 25.06429 | -80.90735 |  |  | GBIF |
| 25.06483 | -80.7737 |  |  | GBIF |
| 25.05169 | -80.90611 |  |  | GBIF |
| 25.05333 | -80.91592 |  |  | GBIF |
| 25.06296 | -80.90038 |  |  | GBIF |
| 25.04073 | -80.92841 |  |  | GBIF |
| 25.0615 | -80.9081 |  |  | GBIF |
| 25.04021 | -80.90291 |  |  | GBIF |
| 25.06183 | -80.91509 |  |  | GBIF |
| 24.98357 | -80.88142 |  |  | GBIF |
| 25.04049 | -80.92713 |  |  | GBIF |
| 24.98548 | -80.88882 |  |  | GBIF |
| 24.96042 | -80.83504 |  |  | GBIF |
| 25.06922 | -80.92747 |  |  | GBIF |
| 24.98029 | -80.85715 |  |  | GBIF |
| 25.04617 | -80.91498 |  |  | GBIF |
| 24.97892 | -80.86894 |  |  | GBIF |
| 24.97833 | -80.84061 |  |  | GBIF |
| 24.99679 | -80.85642 |  |  | GBIF |
| 24.99437 | -80.87826 |  |  | GBIF |
| 24.98771 | -80.84809 |  |  | GBIF |
| 24.99318 | -80.89099 |  |  | GBIF |
| 24.99525 | -80.86906 |  |  | GBIF |
| 24.99927 | -80.87583 |  |  | GBIF |
| 25.00182 | -80.88391 |  |  | GBIF |
| 24.97585 | -80.86868 |  |  | GBIF |
| 24.97174 | -80.89571 |  |  | GBIF |
| 24.99805 | -80.85867 |  |  | GBIF |
| 25.02069 | -80.89223 |  |  | GBIF |
| 25.01701 | -80.88613 |  |  | GBIF |
| 25.00274 | -80.8984 |  |  | GBIF |
| 25.01811 | -80.89259 |  |  | GBIF |
| 25.00903 | -80.87424 |  |  | GBIF |
| 24.97586 | -80.88164 |  |  | GBIF |
| 24.97713 | -80.84436 |  |  | GBIF |
| 25.02352 | -80.87851 |  |  | GBIF |
| 24.96765 | -80.83409 |  |  | GBIF |
| 24.98373 | -80.89905 |  |  | GBIF |
| 25.7574 | -80.1524 |  |  | GBIF |
| 25.30226 | -80.30774 |  |  | GBIF |
| 25.31907 | -80.32618 |  |  | GBIF |
| 25.34324 | -80.31946 |  |  | GBIF |
| 25.35688 | -80.31162 |  |  | GBIF |
| 25.32902 | -80.28867 |  |  | GBIF |
| 25.31854 | -80.30997 |  |  | GBIF |
| 25.32037 | -80.29731 |  |  | GBIF |
| 25.23504 | -80.3476 |  |  | GBIF |
| 25.21704 | -80.3616 |  |  | GBIF |
| 25.2521 | -80.35604 |  |  | GBIF |
| 25.24987 | -80.34528 |  |  | GBIF |
| 25.25958 | -80.35368 |  |  | GBIF |
| 25.21296 | -80.3636 |  |  | GBIF |
| 25.20212 | -80.37921 |  |  | GBIF |
| 25.20217 | -80.38581 |  |  | GBIF |
| 25.05552 | -80.66183 |  |  | GBIF |
| 25.05983 | -80.68548 |  |  | GBIF |
| 25.04704 | -80.69268 |  |  | GBIF |
| 25.06726 | -80.68826 |  |  | GBIF |
| 25.066 | -80.6712 |  |  | GBIF |
| 25.0713 | -80.67416 |  |  | GBIF |
| 25.00038 | -80.65413 |  |  | GBIF |
| 25.06549 | -80.69371 |  |  | GBIF |
| 25.07615 | -80.6806 |  |  | GBIF |
| 25.00373 | -80.61868 |  |  | GBIF |
| 25.11783 | -80.58982 |  |  | GBIF |
| 25.16336 | -80.48167 |  |  | GBIF |
| 25.08788 | -80.58812 |  |  | GBIF |
| 25.10772 | -80.58714 |  |  | GBIF |
| 25.08175 | -80.6012 |  |  | GBIF |
| 25.08578 | -80.61079 |  |  | GBIF |
| 25.09099 | -80.60465 |  |  | GBIF |
| 25.10861 | -80.59549 |  |  | GBIF |
| 25.753816 | -80.154133 |  |  | GBIF |
| 25.312683 | -80.3004 |  |  | GBIF |
| 25.3242 | -80.296333 |  |  | GBIF |
| 25.25315 | -80.351883 |  |  | GBIF |
| 25.25405 | -80.346933 |  |  | GBIF |
| 25.219316 | -80.360483 |  |  | GBIF |
| 25.315116 | -80.3184 |  |  | GBIF |
| 25.318783 | -80.309116 |  |  | GBIF |
| 25.235233 | -80.366333 |  |  | GBIF |
| 25.252016 | -80.361366 |  |  | GBIF |
| 25.270466 | -80.35435 |  |  | GBIF |
| 25.20895 | -80.373166 |  |  | GBIF |
| 25.06595 | -80.672383 |  |  | GBIF |
| 25.1035 | -80.795716 |  |  | GBIF |
| 25.065216 | -80.6559 |  |  | GBIF |
| 25.068183 | -80.690566 |  |  | GBIF |
| 25.105516 | -80.790233 |  |  | GBIF |
| 25.107433 | -80.8115 |  |  | GBIF |
| 25.106683 | -80.804383 |  |  | GBIF |
| 25.076533 | -80.755933 |  |  | GBIF |
| 25.106783 | -80.797483 |  |  | GBIF |
| 25.110483 | -80.80745 |  |  | GBIF |
| 25.078133 | -80.7891 |  |  | GBIF |
| 25.04715 | -80.90315 |  |  | GBIF |
| 25.094383 | -80.745 |  |  | GBIF |
| 25.105633 | -80.785983 |  |  | GBIF |
| 25.090966 | -80.772183 |  |  | GBIF |
| 25.067733 | -80.744283 |  |  | GBIF |
| 25.106733 | -80.7914 |  |  | GBIF |
| 25.074266 | -80.779633 |  |  | GBIF |
| 25.095466 | -80.76345 |  |  | GBIF |
| 25.100433 | -80.779383 |  |  | GBIF |
| 25.047167 | -80.933683 |  |  | GBIF |
| 25.0487 | -80.923117 |  |  | GBIF |
| 25.06897 | -80.772638 |  |  | GBIF |
| 25.06925 | -80.93795 |  |  | GBIF |
| 25.057767 | -80.914567 |  |  | GBIF |
| 25.0975 | -80.774133 |  |  | GBIF |
| 25.048017 | -80.930783 |  |  | GBIF |
| 25.06213 | -80.745483 |  |  | GBIF |
| 25.0665 | -80.785983 |  |  | GBIF |
| 25.054367 | -80.922767 |  |  | GBIF |
| 25.059633 | -80.907467 |  |  | GBIF |
| 25.05475 | -80.9071 |  |  | GBIF |
| 25.051814 | -80.774631 |  |  | GBIF |
| 24.979383 | -80.855483 |  |  | GBIF |
| 25.034783 | -80.91495 |  |  | GBIF |
| 25.063817 | -80.90125 |  |  | GBIF |
| 25.049767 | -80.9141 |  |  | GBIF |
| 25.059817 | -80.930883 |  |  | GBIF |
| 25.057183 | -80.931367 |  |  | GBIF |
| 25.0704 | -80.908133 |  |  | GBIF |
| 25.05785 | -80.940533 |  |  | GBIF |
| 25.062067 | -80.93385 |  |  | GBIF |
| 25.044383 | -80.89935 |  |  | GBIF |
| 25.043533 | -80.928383 |  |  | GBIF |
| 25.064333 | -80.9444 |  |  | GBIF |
| 25.043617 | -80.90805 |  |  | GBIF |
| 24.9977 | -80.875017 |  |  | GBIF |
| 25.065666 | -80.764766 |  |  | GBIF |
| 24.993183 | -80.868767 |  |  | GBIF |
| 24.990267 | -80.899583 |  |  | GBIF |
| 24.9909 | -80.857117 |  |  | GBIF |
| 25.040466 | -80.913133 |  |  | GBIF |
| 24.97725 | -80.84165 |  |  | GBIF |
| 25.001767 | -80.897033 |  |  | GBIF |
| 24.986183 | -80.8856 |  |  | GBIF |
| 24.978883 | -80.864633 |  |  | GBIF |
| 25.020017 | -80.8867 |  |  | GBIF |
| 25.02165 | -80.8776 |  |  | GBIF |
| 25.003267 | -80.864967 |  |  | GBIF |
| 24.9716 | -80.897667 |  |  | GBIF |
| 24.975967 | -80.86115 |  |  | GBIF |
| 24.983317 | -80.911167 |  |  | GBIF |
| 25.01465 | -80.892533 |  |  | GBIF |
| 24.972333 | -80.8445 |  |  | GBIF |
| 25.00125 | -80.886283 |  |  | GBIF |
| 24.995867 | -80.846567 |  |  | GBIF |
| 24.96875 | -80.872017 |  |  | GBIF |
| 25.015217 | -80.8752 |  |  | GBIF |
| 25.163983 | -80.469966 |  |  | GBIF |
| 25.093666 | -80.594366 |  |  | GBIF |
| 25.075716 | -80.593833 |  |  | GBIF |
| 25.0828 | -80.620416 |  |  | GBIF |
| 25.104316 | -80.61835 |  |  | GBIF |
| 25.098133 | -80.609633 |  |  | GBIF |
| 25.102883 | -80.59495 |  |  | GBIF |
| 25.0941 | -80.61715 |  |  | GBIF |
| 25.103 | -80.795983 |  |  | GBIF |
| 25.102366 | -80.789716 |  |  | GBIF |
| 25.104966 | -80.79845 |  |  | GBIF |
| 25.107066 | -80.809333 |  |  | GBIF |
| 25.071816 | -80.78755 |  |  | GBIF |
| 25.107433 | -80.794533 |  |  | GBIF |
| 25.075666 | -80.757816 |  |  | GBIF |
| 25.075733 | -80.733466 |  |  | GBIF |
| 25.1072 | -80.80675 |  |  | GBIF |
| 25.05665 | -80.761116 |  |  | GBIF |
| 25.072666 | -80.7802 |  |  | GBIF |
| 25.082216 | -80.755533 |  |  | GBIF |
| 25.062683 | -80.763266 |  |  | GBIF |
| 25.070716 | -80.770416 |  |  | GBIF |
| 25.081516 | -80.7804 |  |  | GBIF |
| 25.053116 | -80.776066 |  |  | GBIF |
| 25.095216 | -80.781466 |  |  | GBIF |
| 25.079966 | -80.774616 |  |  | GBIF |
| 25.08 | -80.751683 |  |  | GBIF |
| 25.08805 | -80.752733 |  |  | GBIF |
| 25.099016 | -80.765216 |  |  | GBIF |
| 25.092833 | -80.745966 |  |  | GBIF |
| 25.086516 | -80.7639 |  |  | GBIF |
| 25.095417 | -80.76625 |  |  | GBIF |
| 25.757483 | -80.154466 |  |  | GBIF |
| 25.26175 | -80.351983 |  |  | GBIF |
| 25.32183 | -80.31917 |  |  | GBIF |
| 25.31485 | -80.31183 |  |  | GBIF |
| 25.22752 | -80.36818 |  |  | GBIF |
| 25.2353 | -80.35353 |  |  | GBIF |
| 25.25478 | -80.34495 |  |  | GBIF |
| 25.31048 | -80.29798 |  |  | GBIF |
| 25.24377 | -80.36495 |  |  | GBIF |
| 25.20908 | -80.37322 |  |  | GBIF |
| 25.16038 | -80.46327 |  |  | GBIF |
| 25.0817 | -80.61245 |  |  | GBIF |
| 25.10085 | -80.62708 |  |  | GBIF |
| 25.08372 | -80.60308 |  |  | GBIF |
| 25.09062 | -80.60212 |  |  | GBIF |
| 25.03172 | -80.6828 |  |  | GBIF |
| 25.06143 | -80.6814 |  |  | GBIF |
| 25.08213 | -80.69203 |  |  | GBIF |
| 25.01692 | -80.66505 |  |  | GBIF |
| 25.0677 | -80.6623 |  |  | GBIF |
| 25.03517 | -80.93113 |  |  | GBIF |
| 25.06245 | -80.9083 |  |  | GBIF |
| 25.05715 | -80.914566 |  |  | GBIF |
| 25.03857 | -80.9121 |  |  | GBIF |
| 25.045066 | -80.9021 |  |  | GBIF |
| 25.050983 | -80.92055 |  |  | GBIF |
| 25.060566 | -80.932666 |  |  | GBIF |
| 25.060466 | -80.945283 |  |  | GBIF |
| 24.98805 | -80.878566 |  |  | GBIF |
| 25.03767 | -80.90458 |  |  | GBIF |
| 25.059883 | -80.90375 |  |  | GBIF |
| 25.058433 | -80.90855 |  |  | GBIF |
| 24.96595 | -80.839483 |  |  | GBIF |
| 25.060217 | -80.92635 |  |  | GBIF |
| 25.042283 | -80.899417 |  |  | GBIF |
| 25.0696 | -80.935333 |  |  | GBIF |
| 25.057566 | -80.929933 |  |  | GBIF |
| 25.03868 | -80.93295 |  |  | GBIF |
| 25.069183 | -80.904366 |  |  | GBIF |
| 25.004883 | -80.867983 |  |  | GBIF |
| 24.978024 | -80.851584 |  |  | GBIF |
| 24.990033 | -80.883933 |  |  | GBIF |
| 25.03712 | -80.91672 |  |  | GBIF |
| 25.050966 | -80.927516 |  |  | GBIF |
| 24.993166 | -80.851966 |  |  | GBIF |
| 24.980133 | -80.86245 |  |  | GBIF |
| 25.039766 | -80.91485 |  |  | GBIF |
| 25.043633 | -80.906033 |  |  | GBIF |
| 25.013533 | -80.895716 |  |  | GBIF |
| 24.988333 | -80.872116 |  |  | GBIF |
| 24.979066 | -80.834916 |  |  | GBIF |
| 24.956083 | -80.823833 |  |  | GBIF |
| 24.968833 | -80.847316 |  |  | GBIF |
| 24.996366 | -80.896533 |  |  | GBIF |
| 24.99405 | -80.84835 |  |  | GBIF |
| 24.975767 | -80.864617 |  |  | GBIF |
| 25.010066 | -80.874633 |  |  | GBIF |
| 24.972866 | -80.835633 |  |  | GBIF |
| 25.0175 | -80.862066 |  |  | GBIF |
| 25.081883 | -80.651733 |  |  | GBIF |
| 25.061433 | -80.64905 |  |  | GBIF |
| 25.073433 | -80.668966 |  |  | GBIF |
| 25.0751 | -80.680116 |  |  | GBIF |
| 25.0825 | -80.764567 |  |  | GBIF |
| 25.079316 | -80.684333 |  |  | GBIF |
| 25.0648 | -80.68655 |  |  | GBIF |
| 25.096983 | -80.7745 |  |  | GBIF |
| 25.086033 | -80.749033 |  |  | GBIF |
| 25.0594 | -80.931516 |  |  | GBIF |
| 25.073233 | -80.7639 |  |  | GBIF |
| 25.085167 | -80.751667 |  |  | GBIF |
| 25.0703 | -80.7517 |  |  | GBIF |
| 25.059883 | -80.937566 |  |  | GBIF |
| 25.075633 | -80.746 |  |  | GBIF |
| 25.051433 | -80.9405 |  |  | GBIF |
| 25.059966 | -80.76965 |  |  | GBIF |
| 25.0856 | -80.751533 |  |  | GBIF |
| 25.08617 | -80.760667 |  |  | GBIF |
| 25.085266 | -80.781783 |  |  | GBIF |
| 25.083083 | -80.779433 |  |  | GBIF |
| 25.091783 | -80.772416 |  |  | GBIF |
| 25.070716 | -80.9347 |  |  | GBIF |
| 25.0608 | -80.778883 |  |  | GBIF |
| 25.058566 | -80.9205 |  |  | GBIF |
| 25.0777 | -80.772783 |  |  | GBIF |
| 25.057783 | -80.918983 |  |  | GBIF |
| 25.03355 | -80.90835 |  |  | GBIF |
| 25.067433 | -80.917133 |  |  | GBIF |
| 25.043546 | -80.92841 |  |  | GBIF |
| 25.015966 | -80.898 |  |  | GBIF |
| 25.07895 | -80.758166 |  |  | GBIF |
| 25.063316 | -80.77688 |  |  | GBIF |
| 25.059016 | -80.90655 |  |  | GBIF |
| 25.0706 | -80.774833 |  |  | GBIF |
| 25.063833 | -80.937516 |  |  | GBIF |
| 25.050016 | -80.917783 |  |  | GBIF |
| 25.066616 | -80.943066 |  |  | GBIF |
| 25.03325 | -80.908883 |  |  | GBIF |
| 25.075016 | -80.782783 |  |  | GBIF |
| 25.0845 | -80.774733 |  |  | GBIF |
| 25.063466 | -80.9085 |  |  | GBIF |
| 25.059866 | -80.94535 |  |  | GBIF |
| 24.987416 | -80.874666 |  |  | GBIF |
| 24.9838 | -80.899266 |  |  | GBIF |
| 24.987483 | -80.85215 |  |  | GBIF |
| 24.980583 | -80.897383 |  |  | GBIF |
| 24.9986 | -80.883433 |  |  | GBIF |
| 25.006816 | -80.884866 |  |  | GBIF |
| 24.973766 | -80.899516 |  |  | GBIF |
| 25.01555 | -80.876416 |  |  | GBIF |
| 25.060633 | -80.944783 |  |  | GBIF |
| 25.010883 | -80.876483 |  |  | GBIF |
| 25.065083 | -80.9062 |  |  | GBIF |
| 24.987616 | -80.849766 |  |  | GBIF |
| 24.987266 | -80.863683 |  |  | GBIF |
| 24.980183 | -80.86735 |  |  | GBIF |
| 24.99285 | -80.873 |  |  | GBIF |
| 24.9765 | -80.83885 |  |  | GBIF |
| 25.008 | -80.868166 |  |  | GBIF |
| 25.009133 | -80.898516 |  |  | GBIF |
| 24.978883 | -80.877233 |  |  | GBIF |
| 24.997 | -80.895083 |  |  | GBIF |
| 24.98605 | -80.843916 |  |  | GBIF |
| 24.9836 | -80.884583 |  |  | GBIF |
| 25.0245 | -80.889816 |  |  | GBIF |
| 25.0053 | -80.876583 |  |  | GBIF |
| 25.011866 | -80.884683 |  |  | GBIF |
| 24.965066 | -80.840083 |  |  | GBIF |
| 25.168016 | -80.465266 |  |  | GBIF |
| 25.097883 | -80.6113 |  |  | GBIF |
| 25.104333 | -80.6028 |  |  | GBIF |
| 25.757833 | -80.151366 |  |  | GBIF |
| 25.224866 | -80.361683 |  |  | GBIF |
| 25.30875 | -80.30705 |  |  | GBIF |
| 25.263416 | -80.354016 |  |  | GBIF |
| 25.25265 | -80.343533 |  |  | GBIF |
| 25.315366 | -80.300283 |  |  | GBIF |
| 25.30095 | -80.30855 |  |  | GBIF |
| 25.069367 | -80.651933 |  |  | GBIF |
| 25.05235 | -80.683583 |  |  | GBIF |
| 25.072917 | -80.6901 |  |  | GBIF |
| 25.064267 | -80.654233 |  |  | GBIF |
| 25.10495 | -80.790017 |  |  | GBIF |
| 25.063667 | -80.691283 |  |  | GBIF |
| 25.066833 | -80.677167 |  |  | GBIF |
| 25.0588 | -80.751333 |  |  | GBIF |
| 25.0776 | -80.779267 |  |  | GBIF |
| 25.09085 | -80.770717 |  |  | GBIF |
| 25.0433 | -80.7598 |  |  | GBIF |
| 25.07535 | -80.7847 |  |  | GBIF |
| 25.083483 | -80.767517 |  |  | GBIF |
| 25.0668 | -80.786683 |  |  | GBIF |
| 25.069767 | -80.750083 |  |  | GBIF |
| 25.081083 | -80.744 |  |  | GBIF |
| 25.057883 | -80.751 |  |  | GBIF |
| 25.082167 | -80.73265 |  |  | GBIF |
| 25.0559 | -80.774267 |  |  | GBIF |
| 25.076467 | -80.7605 |  |  | GBIF |
| 25.057633 | -80.764467 |  |  | GBIF |
| 25.226616 | -80.364616 |  |  | GBIF |
| 25.373766 | -80.28825 |  |  | GBIF |
| 25.37435 | -80.302316 |  |  | GBIF |
| 25.349133 | -80.2701 |  |  | GBIF |
| 25.217466 | -80.370933 |  |  | GBIF |
| 25.24065 | -80.350216 |  |  | GBIF |
| 25.163683 | -80.467416 |  |  | GBIF |
| 25.31395 | -80.302533 |  |  | GBIF |
| 25.247333 | -80.351566 |  |  | GBIF |
| 25.32935 | -80.302433 |  |  | GBIF |
| 25.094633 | -80.59285 |  |  | GBIF |
| 25.07685 | -80.598917 |  |  | GBIF |
| 25.1069 | -80.592467 |  |  | GBIF |
| 25.086417 | -80.607917 |  |  | GBIF |
| 25.048 | -80.930883 |  |  | GBIF |
| 25.034917 | -80.925783 |  |  | GBIF |
| 25.04695 | -80.9337 |  |  | GBIF |
| 25.058017 | -80.940867 |  |  | GBIF |
| 25.0619 | -80.933833 |  |  | GBIF |
| 25.064283 | -80.94435 |  |  | GBIF |
| 25.057717 | -80.9146 |  |  | GBIF |
| 25.063833 | -80.901283 |  |  | GBIF |
| 25.0596 | -80.930917 |  |  | GBIF |
| 25.054533 | -80.907067 |  |  | GBIF |
| 25.048617 | -80.923033 |  |  | GBIF |
| 25.059667 | -80.907533 |  |  | GBIF |
| 25.0497 | -80.914067 |  |  | GBIF |
| 25.068383 | -80.915833 |  |  | GBIF |
| 25.0435 | -80.928633 |  |  | GBIF |
| 25.07025 | -80.926017 |  |  | GBIF |
| 25.05415 | -80.923133 |  |  | GBIF |
| 25.034933 | -80.914917 |  |  | GBIF |
| 25.042833 | -80.89585 |  |  | GBIF |
| 25.070317 | -80.908217 |  |  | GBIF |
| 25.06925 | -80.937933 |  |  | GBIF |
| 24.980667 | -80.835083 |  |  | GBIF |
| 24.9861 | -80.8853 |  |  | GBIF |
| 25.0398 | -80.9214 |  |  | GBIF |
| 25.0404 | -80.913317 |  |  | GBIF |
| 24.984417 | -80.875267 |  |  | GBIF |
| 24.9904 | -80.8994 |  |  | GBIF |
| 24.961867 | -80.843933 |  |  | GBIF |
| 25.007567 | -80.882267 |  |  | GBIF |
| 25.043467 | -80.908067 |  |  | GBIF |
| 24.979067 | -80.914433 |  |  | GBIF |
| 24.990667 | -80.885967 |  |  | GBIF |
| 25.019933 | -80.886567 |  |  | GBIF |
| 24.993433 | -80.86855 |  |  | GBIF |
| 25.009167 | -80.8925 |  |  | GBIF |
| 24.998083 | -80.84785 |  |  | GBIF |
| 25.001867 | -80.89705 |  |  | GBIF |
| 25.0033 | -80.864933 |  |  | GBIF |
| 24.968467 | -80.83285 |  |  | GBIF |
| 24.97965 | -80.85525 |  |  | GBIF |
| 25.018267 | -80.8735 |  |  | GBIF |
| 24.997717 | -80.874833 |  |  | GBIF |
| 24.979033 | -80.90215 |  |  | GBIF |
| 24.99095 | -80.856767 |  |  | GBIF |
| 25.0013 | -80.8861 |  |  | GBIF |
| 24.972917 | -80.865283 |  |  | GBIF |
| 24.971617 | -80.8974 |  |  | GBIF |
| 24.972367 | -80.8444 |  |  | GBIF |
| 24.974283 | -80.87915 |  |  | GBIF |
| 25.755983 | -80.156167 |  |  | GBIF |
| 25.758767 | -80.1491 |  |  | GBIF |
| 16.8028 | -88.0821 |  |  | GBIF |
| 16.7881 | -88.0829 |  |  | GBIF |
| 16.8035 | -88.0834 |  |  | GBIF |
| 19.780556 | -90.622222 |  |  | GBIF |
| 27.72694 | -97.16639 |  |  | GBIF |
| 16.6647 | -88.1911 |  |  | GBIF |
| 16.18 | -61.1 |  |  | GBIF |
| 21.258333 | -89.741667 |  |  | GBIF |
| 20.858333 | -90.375 |  |  | GBIF |
| 21.258333 | -89.725 |  |  | GBIF |
| 20.825 | -90.391667 |  |  | GBIF |
| 21.258333 | -89.708333 |  |  | GBIF |
| 21.275 | -89.675 |  |  | GBIF |
| 20.841667 | -90.391667 |  |  | GBIF |
| 20.775 | -90.441667 |  |  | GBIF |
| 20.791667 | -90.408333 |  |  | GBIF |
| 21.275 | -89.691667 |  |  | GBIF |
| 21.258333 | -89.758333 |  |  | GBIF |
| 21.258333 | -89.691667 |  |  | GBIF |
| 24.83 | -80.78 |  |  | GBIF |
| -7.75162931833411 | -34.8252187095818 |  |  | GBIF |
| 27.15 | -82.51667 |  |  | GBIF |
| 21.275 | -89.708333 |  |  | GBIF |
| 20.875 | -90.358333 |  |  | GBIF |
| 17.75 | -88.033333 |  |  | GBIF |
| 17.75 | -88.03333 |  |  | GBIF |
| 27.62917 | -97.21694 |  |  | GBIF |
| 17.7523 | -64.7195 |  |  | GBIF |
| 23.77 | -76 |  |  | GBIF |
| 11.328365 | -74.13878 |  |  | GBIF |
| 24.583333 | -81.633333 |  |  | GBIF |
| 24.862635 | -80.717287 |  |  | GBIF |
| 22.3825 | -89.6825 |  |  | GBIF |
| 12.166667 | -83.05 |  |  | GBIF |
| 21.605556 | -87.102222 |  |  | GBIF |
| 18.579444 | -87.33 |  |  | GBIF |
| 18.583333 | -87.333333 |  |  | GBIF |
| 21.256111 | -86.749167 |  |  | GBIF |
| 21.258056 | -86.751667 |  |  | GBIF |
| 21.591667 | -88.161111 |  |  | GBIF |
| 16.808889 | -88.081389 |  |  | GBIF |
| 21.572222 | -88.1 |  |  | GBIF |
| 19.5825 | -96.383333 |  |  | GBIF |
| 21.24 | -89.72 |  |  | GBIF |
| 19.57 | -96.38 |  |  | GBIF |
| 19.585833 | -96.384444 |  |  | GBIF |
| 14.4675 | -82.7 |  |  | GBIF |
| 19.101389 | -95.938611 |  |  | GBIF |
| 19.0665844196311 | -95.9288764332644 |  |  | GBIF |
| 23.1 | -74.983333 |  |  | GBIF |
| 16.2 | -88.216667 |  |  | GBIF |
| 16.113333 | -88.248055 |  |  | GBIF |
| 16.130278 | -88.245 |  |  | GBIF |
| 16.7 | -87.75 |  |  | GBIF |
| 16.7 | -87.833333 |  |  | GBIF |
| 16.725 | -87.791667 |  |  | GBIF |
| 28.73 | -82.75 |  |  | GBIF |
| 19.199444 | -96.066944 |  |  | GBIF |
| 24.67 | -82.8917 |  |  | GBIF |
| 15.5911 | -61.3391 |  |  | GBIF |
| 15.4 | -61.425 |  |  | GBIF |
| 24.638194 | -81.34425 |  |  | GBIF |
| 21.677698 | -82.99739 |  |  | GBIF |
| 17.943389 | -67.191065 |  |  | GBIF |
| 18.316215 | -65.229336 |  |  | GBIF |
| 17.948052 | -66.922989 |  |  | GBIF |
| 21.6 | -88.15 |  |  | GBIF |
| 21.27 | -89.75 |  |  | GBIF |
| 20.7233 | -90.4647 |  |  | GBIF |
| 21.265833 | -89.691111 |  |  | GBIF |
| 21.5488 | -87.2079 |  |  | GBIF |
| 21.150486 | -86.785511 |  |  | GBIF |
| 20.8746 | -90.394 |  |  | GBIF |
| 19.21 | -96.102 |  |  | GBIF |
| 20.86 | -90.4 |  |  | GBIF |
| 19.19 | -96.1 |  |  | GBIF |
| 21.46 | -97.22 |  |  | GBIF |
| 21.2723 | -89.7389 |  |  | GBIF |
| 20.747222 | -90.434167 |  |  | GBIF |
| 19.21 | -96.09 |  |  | GBIF |
| 21.269722 | -89.688333 |  |  | GBIF |
| 20.47 | -90.48 |  |  | GBIF |
| 19.5 | -87.416667 |  |  | GBIF |
| 21.57 | -88.23 |  |  | GBIF |
| 19.17 | -96.04 |  |  | GBIF |
| 21.16 | -90.2 |  |  | GBIF |
| 21.025953 | -86.8 |  |  | GBIF |
| 19.06 | -95.97 |  |  | GBIF |
| 21.130944 | -86.750833 |  |  | GBIF |
| 21.426738 | -86.83647 |  |  | GBIF |
| 11.948889 | -66.676111 |  |  | GBIF |
| 27.77 | -97.13 |  |  | GBIF |
| 27.54 | -97.27 |  |  | GBIF |
| -8.3699 | -34.9611 |  |  | OBIS |
| 17.9189300537 | -67.0124816895 |  |  | OBIS |
| 17.9066009521 | -67.0981063843 |  |  | OBIS |
| -7.7441 | -34.8193 |  |  | OBIS |
| 18.3566608429 | -64.7468414307 |  |  | OBIS |
| 17.7915096283 | -64.6266098022 |  |  | OBIS |
| 17.9262504578 | -66.9887084961 |  |  | OBIS |
| 17.7640705109 | -64.6341018677 |  |  | OBIS |
| -7.7566 | -34.819 |  |  | OBIS |
| -8.5369 | -35.0089 |  |  | OBIS |
| 17.7252006531 | -64.633102417 |  |  | OBIS |
| -8.3565 | -34.9528 |  |  | OBIS |
| 17.7611999512 | -64.6283493042 |  |  | OBIS |
| -8.3658 | -34.9541 |  |  | OBIS |
| 18.3537693024 | -64.7578582764 |  |  | OBIS |
| 17.9254302979 | -66.9573516846 |  |  | OBIS |
| 17.9176197052 | -66.9623336792 |  |  | OBIS |
| -20.18528 | -40.19127 |  |  | OBIS |
| 17.7421092987 | -64.6023483276 |  |  | OBIS |
| -17.605278 | -39.050833 |  |  | OBIS |
| -17.875 | -38.979167 |  |  | OBIS |
| 17.7918605804 | -64.6249771118 |  |  | OBIS |
| 17.9240207672 | -66.9856567383 |  |  | OBIS |
| 17.760559082 | -64.5962600708 |  |  | OBIS |
| 17.7219696045 | -64.6501312256 |  |  | OBIS |
| 17.9387893677 | -67.0960006714 |  |  | OBIS |
| 18.3099098206 | -64.7530593872 |  |  | OBIS |
| 17.9133300781 | -66.9977035522 |  |  | OBIS |
| 17.9398498535 | -67.1067581177 |  |  | OBIS |
| 17.765209198 | -64.6312789917 |  |  | OBIS |
| 17.7611293793 | -64.6198272705 |  |  | OBIS |
| 17.7906894684 | -64.6212081909 |  |  | OBIS |
| 17.935760498 | -67.0960388184 |  |  | OBIS |
| 17.9291496277 | -66.9880218506 |  |  | OBIS |
| 17.7610702515 | -64.6062698364 |  |  | OBIS |
| -8.3762 | -34.9566 |  |  | OBIS |
| 17.760799408 | -64.5782775879 |  |  | OBIS |
| 18.320810318 | -64.7650299072 |  |  | OBIS |
| 17.937040329 | -66.9645767212 |  |  | OBIS |
| 17.9090309143 | -67.1202087402 |  |  | OBIS |
| 17.7900009155 | -64.6163482666 |  |  | OBIS |
| 17.7592792511 | -64.591293335 |  |  | OBIS |
| 18.3449707031 | -64.6686706543 |  |  | OBIS |
| 18.3156890869 | -64.7666702271 |  |  | OBIS |
| 18.3571891785 | -64.7478790283 |  |  | OBIS |
| 17.918340683 | -67.0924835205 |  |  | OBIS |
| 18.3201599121 | -64.7478408813 |  |  | OBIS |
| 18.3285694122 | -64.7993087769 |  |  | OBIS |
| 17.7609500885 | -64.5867614746 |  |  | OBIS |
| 18.3502502441 | -64.7831115723 |  |  | OBIS |
| 18.3143005371 | -64.7625427246 |  |  | OBIS |
| 17.7915897369 | -64.6278381348 |  |  | OBIS |
| 17.9298000336 | -66.947593689 |  |  | OBIS |
| 17.9135608673 | -66.9956665039 |  |  | OBIS |
| 17.9280891418 | -66.9945373535 |  |  | OBIS |
| 18.34608078 | -64.6677780151 |  |  | OBIS |
| 17.7903308868 | -64.6209411621 |  |  | OBIS |
| 18.3482704163 | -64.7929534912 |  |  | OBIS |
| 17.9174900055 | -66.9766998291 |  |  | OBIS |
| 17.7905197144 | -64.6231994629 |  |  | OBIS |
| 18.3479003906 | -64.7038421631 |  |  | OBIS |
| 17.9398899078 | -66.257232666 |  |  | OBIS |
| 17.7898406982 | -64.6197662354 |  |  | OBIS |
| 17.9515705109 | -66.9926528931 |  |  | OBIS |
| 17.759519577 | -64.5992202759 |  |  | OBIS |
| 18.3149700165 | -64.766960144 |  |  | OBIS |
| 17.9233608246 | -66.9832611084 |  |  | OBIS |
| 17.7899799347 | -64.6203994751 |  |  | OBIS |
| 18.3571891785 | -64.7462768555 |  |  | OBIS |
| 17.7918395996 | -64.6268386841 |  |  | OBIS |
| 18.3218994141 | -64.7889480591 |  |  | OBIS |
| 17.926410675 | -67.1061706543 |  |  | OBIS |
| 17.7843608856 | -64.6204299927 |  |  | OBIS |
| 18.3186206818 | -64.7471237183 |  |  | OBIS |
| 17.9288196564 | -67.1163406372 |  |  | OBIS |
| 17.7608890533 | -64.5915679932 |  |  | OBIS |
| 17.7912406921 | -64.6293792725 |  |  | OBIS |
| 17.9306793213 | -67.1123504639 |  |  | OBIS |
| 17.9419002533 | -66.9637680054 |  |  | OBIS |
| 17.7583293915 | -64.6070632935 |  |  | OBIS |
| 17.9240093231 | -66.9976730347 |  |  | OBIS |
| 17.7894706726 | -64.6180267334 |  |  | OBIS |
| 17.9312591553 | -66.9458312988 |  |  | OBIS |
| 17.7646598816 | -64.6360931396 |  |  | OBIS |
| 18.3595790863 | -64.7445526123 |  |  | OBIS |
| 17.7909107208 | -64.6233901978 |  |  | OBIS |
| 17.928899765 | -67.1050872803 |  |  | OBIS |
| 17.9188899994 | -67.0521697998 |  |  | OBIS |
| 17.9349899292 | -66.9621505737 |  |  | OBIS |
| 18.3151092529 | -64.7767181396 |  |  | OBIS |
| 17.9490394592 | -66.9761123657 |  |  | OBIS |
| 17.9338207245 | -67.0935974121 |  |  | OBIS |
| 18.3203392029 | -64.7469177246 |  |  | OBIS |
| 17.7830600739 | -64.632270813 |  |  | OBIS |
| 17.7614192963 | -64.6032562256 |  |  | OBIS |
| 17.7632904053 | -64.6213912964 |  |  | OBIS |
| -17.708056 | -39.000833 |  |  | OBIS |
| 17.79088974 | -64.6185684204 |  |  | OBIS |
| 17.9086894989 | -67.0173034668 |  |  | OBIS |
| 18.3193092346 | -64.7497177124 |  |  | OBIS |
| 18.3279495239 | -64.7995376587 |  |  | OBIS |
| 17.9223403931 | -66.9955215454 |  |  | OBIS |
| 17.9333591461 | -67.0952606201 |  |  | OBIS |
| 18.3143806458 | -64.7749481201 |  |  | OBIS |
| -17.906944 | -38.690278 |  |  | OBIS |
| 18.3209495544 | -64.7650985718 |  |  | OBIS |
| 17.9475097656 | -66.9788131714 |  |  | OBIS |
| 17.7582092285 | -64.6058425903 |  |  | OBIS |
| 17.7908592224 | -64.6191177368 |  |  | OBIS |
| 17.9265193939 | -67.1131286621 |  |  | OBIS |
| 17.9232406616 | -66.9986877441 |  |  | OBIS |
| 17.7595806122 | -64.5884933472 |  |  | OBIS |
| 18.3474006653 | -64.7875976562 |  |  | OBIS |
| 17.9303092957 | -66.9517593384 |  |  | OBIS |
| 18.3180599213 | -64.772819519 |  |  | OBIS |
| 17.924539566 | -67.0116271973 |  |  | OBIS |
| 17.7850704193 | -64.6250228882 |  |  | OBIS |
| 17.942199707 | -67.0947494507 |  |  | OBIS |
| 18.3558292389 | -64.7822875977 |  |  | OBIS |
| 17.9177093506 | -67.0574264526 |  |  | OBIS |
| 17.7851600647 | -64.6147537231 |  |  | OBIS |
| 17.9444007874 | -67.0809936523 |  |  | OBIS |
| 17.9367904663 | -66.9246902466 |  |  | OBIS |
| 17.7839508057 | -64.6190719604 |  |  | OBIS |
| 17.7634391785 | -64.6220703125 |  |  | OBIS |
| 17.7227592468 | -64.6330566406 |  |  | OBIS |
| 24.5628333333 | -82.1071666667 |  |  | OBIS |
| 17.9509296417 | -66.9914016724 |  |  | OBIS |
| 17.7899894714 | -64.6160507202 |  |  | OBIS |
| 17.7618904114 | -64.6081619263 |  |  | OBIS |
| 17.7910995483 | -64.6277770996 |  |  | OBIS |
| 17.9412708282 | -67.0430679321 |  |  | OBIS |
| 17.7902202606 | -64.6205673218 |  |  | OBIS |
| 17.784330368 | -64.6249237061 |  |  | OBIS |
| 17.9347000122 | -66.9593734741 |  |  | OBIS |
| 17.7606105804 | -64.6258773804 |  |  | OBIS |
| 17.9112892151 | -67.070892334 |  |  | OBIS |
| 17.7902393341 | -64.6169281006 |  |  | OBIS |
| 17.9338302612 | -66.959602356 |  |  | OBIS |
| 17.9243106842 | -67.0003433228 |  |  | OBIS |
| 17.9270706177 | -67.1033630371 |  |  | OBIS |
| 17.9364490509 | -67.0953369141 |  |  | OBIS |
| 18.3182506561 | -64.7649383545 |  |  | OBIS |
| 17.7216091156 | -64.6472473145 |  |  | OBIS |
| 17.9472293854 | -66.9985733032 |  |  | OBIS |
| 17.9268093109 | -67.1005020142 |  |  | OBIS |
| 18.3313102722 | -64.7971878052 |  |  | OBIS |
| 17.7635402679 | -64.5852432251 |  |  | OBIS |
| 17.7899608612 | -64.6218338013 |  |  | OBIS |
| 17.7621192932 | -64.6119918823 |  |  | OBIS |
| 18.3098201752 | -64.7181015015 |  |  | OBIS |
| 17.9489898682 | -67.0361175537 |  |  | OBIS |
| 27.4702758789 | -82.6907272339 |  |  | OBIS |
| 17.9502696991 | -66.9909286499 |  |  | OBIS |
| 17.7855892181 | -64.6139526367 |  |  | OBIS |
| 17.9228305817 | -67.0189666748 |  |  | OBIS |
| 27.3733863831 | -97.3691329956 |  |  | OBIS |
| 21.5704536438 | -87.8837127686 |  |  | OBIS |
| 22.279958725 | -97.7937316895 |  |  | OBIS |
| 17.7869701385 | -64.6305465698 |  |  | OBIS |
| 17.9127502441 | -67.1214828491 |  |  | OBIS |
| 17.9360198975 | -66.9605484009 |  |  | OBIS |
| 18.3176193237 | -64.772857666 |  |  | OBIS |
| 17.7633495331 | -64.5857086182 |  |  | OBIS |
| 17.9096794128 | -67.0186233521 |  |  | OBIS |
| 17.7573108673 | -64.6024017334 |  |  | OBIS |
| 17.905790329 | -67.1047973633 |  |  | OBIS |
| 18.3484306335 | -64.7047271729 |  |  | OBIS |
| 18.3436107635 | -64.7871780396 |  |  | OBIS |
| 17.9176197052 | -66.9924926758 |  |  | OBIS |
| 18.3576393127 | -64.7478790283 |  |  | OBIS |
| 18.313999176 | -64.7774887085 |  |  | OBIS |
| 17.7916908264 | -64.626411438 |  |  | OBIS |
| 18.3170700073 | -64.7709732056 |  |  | OBIS |
| 17.9411697388 | -67.0479125977 |  |  | OBIS |
| 17.7915306091 | -64.6240081787 |  |  | OBIS |
| 18.3489494324 | -64.6701126099 |  |  | OBIS |
| 17.9465694427 | -67.0136566162 |  |  | OBIS |
| 17.7458896637 | -64.5962677002 |  |  | OBIS |
| 18.3542900085 | -64.7809524536 |  |  | OBIS |
| 17.7265090942 | -64.6321563721 |  |  | OBIS |
| 17.9352092743 | -66.9770507812 |  |  | OBIS |
| 17.7907791138 | -64.6187973022 |  |  | OBIS |
| 17.932800293 | -66.9740219116 |  |  | OBIS |
| 18.3535499573 | -64.7716827393 |  |  | OBIS |
| 17.7905693054 | -64.6226730347 |  |  | OBIS |
| 18.3201007843 | -64.7629623413 |  |  | OBIS |
| 17.9493999481 | -66.9937896729 |  |  | OBIS |
| 17.7904396057 | -64.6208267212 |  |  | OBIS |
| 17.7409095764 | -64.6015167236 |  |  | OBIS |
| 18.3575592041 | -64.6614379883 |  |  | OBIS |
| 18.3141403198 | -64.7737884521 |  |  | OBIS |
| 17.9363193512 | -67.1144104004 |  |  | OBIS |
| 12.5496205538 | -81.6890144348 |  |  | iNaturalist |
| 26.1296557216 | -97.1668805928 |  |  | iNaturalist |
| 29.6962374263 | -85.3703360586 |  |  | iNaturalist |
| 25.69821 | -80.154366 |  |  | iNaturalist |
| 18.3497327881 | -64.9904981007 |  |  | iNaturalist |
| 18.350242137 | -64.992340403 |  |  | iNaturalist |
| 18.341996764 | -64.9769126255 |  |  | iNaturalist |
| 25.124469202 | -80.4011408418 |  |  | iNaturalist |
| 12.467077 | -61.432267 |  |  | iNaturalist |
| 19.8599189889 | -87.5377308883 |  |  | iNaturalist |
| 18.3416749442 | -64.9767896529 |  |  | iNaturalist |
| 18.3493047207 | -64.9896055718 |  |  | iNaturalist |
| 18.3491399013 | -64.9913693501 |  |  | iNaturalist |
| 18.3466225618 | -64.7782299083 |  |  | iNaturalist |
| 18.3529389126 | -64.7597255606 |  |  | iNaturalist |
| 18.2785461196 | -64.8938697286 |  |  | iNaturalist |
| 18.3447566145 | -65.0149506509 |  |  | iNaturalist |
| 18.386765 | -64.512985 |  |  | iNaturalist |
| 18.3866666667 | -64.5127777778 |  |  | iNaturalist |
| 18.4997945887 | -64.358210372 |  |  | iNaturalist |
| 18.5035850096 | -64.3616435996 |  |  | iNaturalist |
| 18.4454637226 | -64.6495262372 |  |  | iNaturalist |
| 18.4448060039 | -64.6507360246 |  |  | iNaturalist |
| 18.4423887148 | -64.7658857209 |  |  | iNaturalist |
| 18.4418458107 | -64.7649132602 |  |  | iNaturalist |
| 18.3544444444 | -64.5688888889 |  |  | iNaturalist |
| 18.4237011912 | -64.6588216835 |  |  | iNaturalist |
| 12.476284507 | -61.480169613 |  |  | iNaturalist |
| 12.4571098094 | -61.4832081779 |  |  | iNaturalist |
| 12.4853530882 | -61.4569107927 |  |  | iNaturalist |
| 21.5176003016 | -97.3542221379 |  |  | iNaturalist |
| 19.328134111 | -69.4563526002 |  |  | iNaturalist |
| 18.3395683333 | -64.9785033333 |  |  | iNaturalist |
| 18.33933 | -64.9787266667 |  |  | iNaturalist |
| 18.3543304196 | -64.6941224157 |  |  | iNaturalist |
| 25.1257621103 | -80.4048619785 |  |  | iNaturalist |
| 19.3348029045 | -90.7837921015 |  |  | iNaturalist |
| 19.323658 | -69.48674 |  |  | iNaturalist |
| 19.325133 | -69.48246 |  |  | iNaturalist |
| 27.511815319 | -82.7229313481 |  |  | iNaturalist |
| 29.312241 | -94.7670549722 |  |  | iNaturalist |
| 17.9553959086 | -66.2184492086 |  |  | iNaturalist |
| 29.7050242625 | -84.777998887 |  |  | iNaturalist |
| 19.5063 | -16.42793333 |  |  | iNaturalist |

Table S6 – Eight loci used for microsatellite amplification (Larkin et al., 2012; 2017)

| **Locus** | **Genbank accession number** | **Primer sequence (5'-3')** | **Fluorescent dyes** |
| --- | --- | --- | --- |
| Hw180 | JN614999 | F: GTGGAGGCCGAACTGTATCT | FAM |
|  |  | R: CGACCTTCATCCTAATCATCG |  |
| Hw188 | JN615000 | F: ACCTTCATAAATGGCAACTTG | ATTO550 |
|  |  | R: CAACTTGGTTCTGGTAGTCATC |  |
| Hw190 | JN615001 | F: ATGACGAATCCCGAGGTAT | HEX |
|  |  | R: CCAGGTATTGTCGCTTTCA |  |
| Hw190b | KT002048 | F: ATGACGAATCCCGAGGTAT | HEX |
|  |  | R: GTTTCACCCACGTTAAAGCACAAT |  |
| Hw196 | JN615002 | F: ACAACCTAGATCATCCTCACAC | HEX |
|  |  | R: AGCAGGAAGTCAAGAGATAGG |  |
| Hw200 | JN615003 | F: TTATGGGATCTATTTGTGGTCT | ATTO565 |
|  |  | R: TTTTGCTTTGTAGTCTTGGTG |  |
| Hw212 | JN615005 | F: ATGGATGTTCATTGAGTTTGAC | FAM |
|  |  | R: CAAGGCTAAGGTAGTGGACC |  |
| Hw228 | KT002050 | F: AAGACGGCATTGGAAAATAAG | ATTO550 |
|  |  | R: GTTTGGTATCATCGGAAGCACTGT |  |


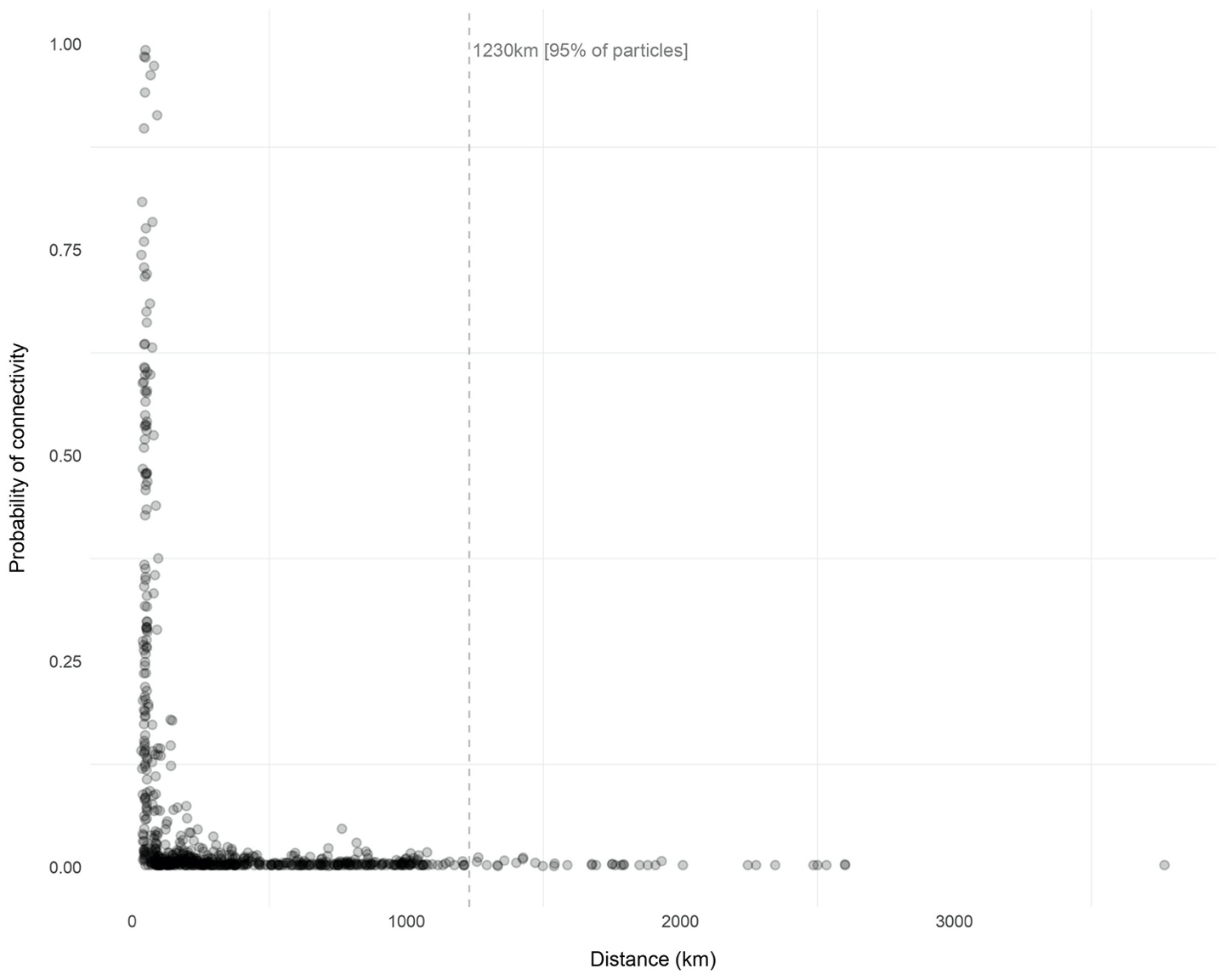


Figure S1 – Probability of connectivity of the dispersal units according to distance (km).


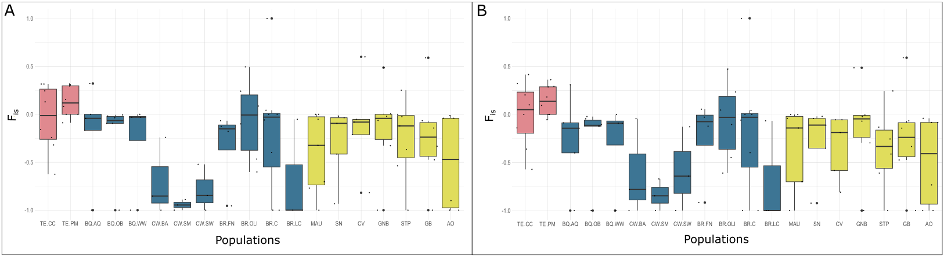


Figure S2 – Inbreeding coefficient (F_IS_) by location, a) calculated for the ramet-level data set and b) calculated for the genet-level data set. Each point represents the F_IS_ value for one locus per location.


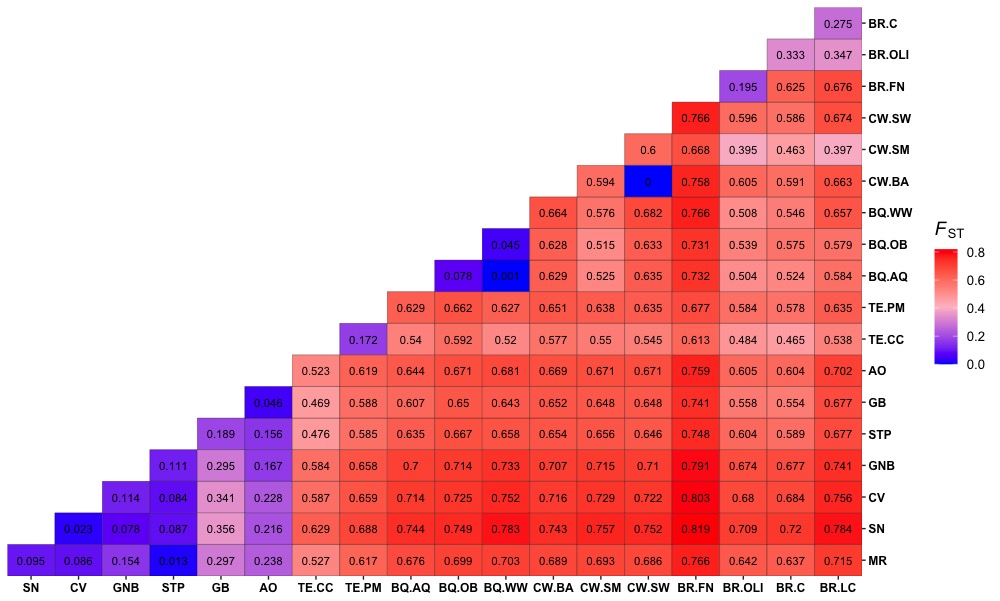


Figure S3 – Population pairwise differentiation FST between *Halodule wrightii* populations.


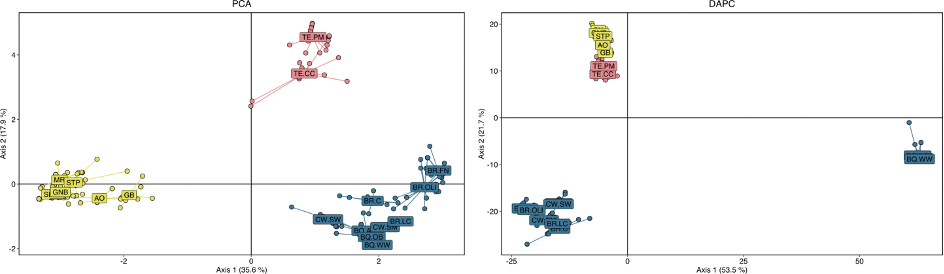


Figure S4 – Principal Components Analysis (PCA) and discriminant Analysis of Principal Components (DAPC) among *Halodule wrightii* populations.
